# Synthesis, Characterization, and Functionalization of 1‐Boraphenalenes

**DOI:** 10.1002/anie.201803180

**Published:** 2018-06-06

**Authors:** Rachel J. Kahan, Daniel L. Crossley, Jessica Cid, James E. Radcliffe, Michael J. Ingleson

**Affiliations:** ^1^ School of Chemistry University of Manchester Manchester M13 9PL UK

**Keywords:** aromaticity, boron, borylation, phenalenyl, polycyclic aromatic hydrocarbons

## Abstract

1‐Boraphenalenes have been synthesized by reaction of BBr_3_ with 1‐(aryl‐ethynyl)naphthalenes, 1‐ethynylnaphthalene, and 1‐(pent‐1‐yn‐1‐yl)naphthalene and they can be selectively functionalized at boron or carbon to form bench‐stable products. All of these 1‐boraphenalenes have LUMOs localized on the planar C_12_B core that are closely comparable in character to isoelectronic phenalenyl cations. In contrast to the comparable LUMOs, the aromatic stabilization of the C_5_B ring in 1‐boraphenalenes is dramatically lower than the C_6_ rings in phenalenyl cations. This is due to the occupied orbitals of π symmetry being less delocalised in the 1‐boraphenalenes.

Phenalenyl (**1**) is an open‐shell polyaromatic hydrocarbon (PAH) containing 13 carbon atoms and 13 π electrons.^1^ Since Haddon's seminal report in 1975,^2^ **1**, and derivatives, have been of considerable interest for studying fundamental bonding phenomena (multi‐centre bonding/σ Vs. π dimerisation),^2^, ^3^ and for a range of applications (organic semiconductors, spin memory, electrode materials).^4^ The non‐bonding SOMO of phenalenyl is key to its unique properties (Figure 1), and phenalenyls display amphoteric redox behaviour, with oxidation furnishing a 12 π electron cation and reduction a 14 π electron anion.^1^ The key properties of **1** can be modulated by functionalisation of the periphery or by incorporation of heteroatoms.^5^, ^6^ While the incorporation of N, O, and S into phenalenyls is well‐documented,^1^, ^5^, ^7^ there are only two reports incorporating boron to the best of our knowledge, and in both boron is co‐doped with nitrogen (for example, **2** and **3**; Figure 1).^8^, ^9^, ^10^ However, computational studies on boraphenalenes have indicated potentially interesting molecular and bulk properties.^11^

**Figure 1:**
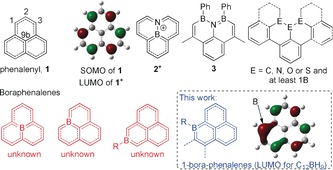
Top: phenalenyl, **1**, and reported B,N‐phenalenes/ extended B‐E phenalenes.^8^, ^9^, ^12^ Bottom: boraphenalene isomers. The LUMOs are calculated at the B3LYP/6–311G(d,p) level (0.05 isovalue).

While notable work on di‐ and tetrabenzophenalenes containing boron (with and without co‐doping with N/O/S) has been reported,^12^ these more extended compounds have distinct electronic structures and thus are not directly comparable to the phenalenyls. Even **3** which has a tricyclic core isoelectronic to **1^+^** has some LUMO character located on the exocyclic aromatic groups and is not completely planar (Supporting Information, Figure S8), and therefore is distinct to **1^+^**. To generate a boron‐doped PAH more comparable to the phenalenyl cation, the analogue should be planar, be isoelectronic to **1^+^**, and have a LUMO that is closely comparable in character to **1^+^**. Notably our calculations indicate this is the case for the 1‐boraphenalenes (for example, Figure 1, bottom right).

Recently, a number of routes have been developed to synthesize B‐doped PAHs.^6^, ^13^ In this area the combination of alkyne borylative cyclisation^14^ and intramolecular boron‐Friedel Crafts enabled the formation of boracycles (Scheme 1, top).^15^ Compound **B** and derivatives (Scheme 1, top right) do contain a 1‐boraphenalene (C_12_B) subunit. However, the additional fused rings present in **B** leads to non‐planarity within the C_12_B subunit and LUMOs that are delocalised beyond the tricyclic subunit. Herein we report the serendipitous synthesis of a planar 1‐boraphenalene containing no additional annulation. This enabled the subsequent development of a simple route to 1‐boraphenalenes, which can be readily functionalized at varying positions. Calculations revealed comparable LUMOs for these 1‐boraphenalenes and isoelectronic **1^+^**, however, the occupied π orbitals of the 1‐boraphenalenes are distinct in character to **1^+^**, leading to lower aromatic stabilization of the C_5_B ring.

**Scheme 1:**
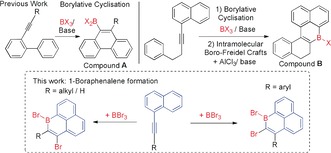
Top: the combined borylative cyclisation/intramolecular S_E_Ar reaction. Bottom: this work, to form 1,2‐ and 1,3‐dibromo‐1‐boraphenalenes.

Previously, the cyclisation of 2‐(phenylethynyl)‐1,1′‐biphenyl led to the 9‐borylated phenanthrene, compound **A** (Scheme 1, top left).^14^ However, on attempting the borylative cyclisation of **4 a** the expected compound, **C** (Scheme 2, left), was not observed. Instead the addition of BBr_3_ in *ortho*‐dichlorobenzene (*o*‐DCB) and heating to 140 °C led on workup to the 1‐hydroxy‐1‐boraphenalene, **6 a** (Scheme 2, right). Along with **6 a**, the formation of products from HBr addition to the triple bond of **4 a** were observed (HBr is the by‐product from the S_E_Ar reaction). Repeating the reaction in the presence of 2,4,6‐tri‐*tert*‐butylpyridine (TBP) and using excess BBr_3_ (as [H‐TBP][BBr_4_] is now the ultimate by‐product from S_E_Ar) prevents this side reaction and leads to good yields of **6 a**. Analogous reactivity is observed when terphenyl is replaced by *p*‐tolyl and **6 c** also was isolated as a bench stable solid. In contrast, the formation of **5 b** was complicated by the 6‐*endo*‐dig cyclisation to form the 9‐borylated phenanthrene (analogous to **C**) which is competitive in this case, with a 1:1 ratio of products formed (see the Supporting Information). For **4 a**, the 6‐*endo*‐dig cyclisation presumably is disfavoured because of the greater steric bulk around the alkyne giving rise exclusively to **5 a** (while the 6‐*endo*‐dig cyclisation is not possible with **4 c**). The ^1^H NMR spectrum of **6 a** has a characteristic broad singlet at *δ*=5.75 ppm for the B−OH, which is at 5.97 ppm for **6 b** and 6.11 ppm for **6 c**. The ^11^B NMR resonances for **6 a** −**6 c** are typical for boracyclic borinic acids (δ_11B_=37–38 ppm).

**Scheme 2:**
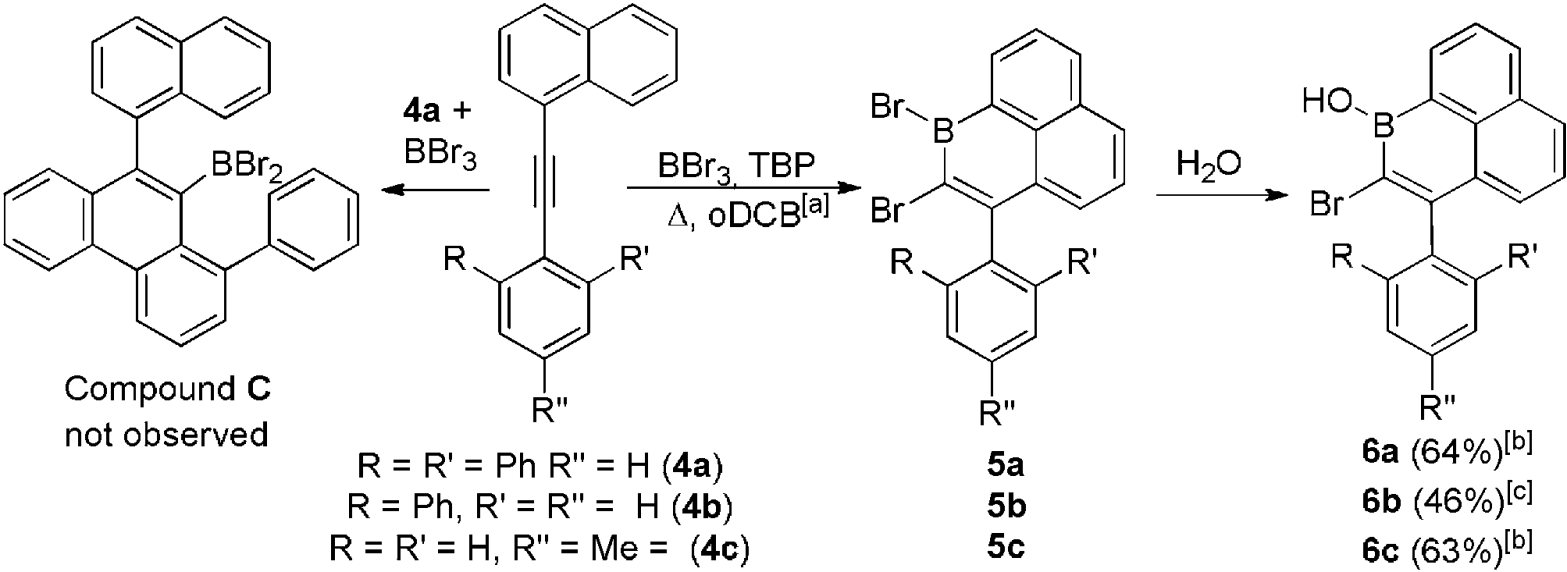
1‐Boraphenalene formation. [a] Heated to 140 °C for **4 a**; 120 °C for **4 b**; and 70 °C for **4 c**. [b] Yield of isolated product. [c] In situ conversion.

Single‐crystal X‐ray diffraction studies on **6 a** and **6 c** (Figure 2) revealed a trigonal planar geometry around boron, effectively orthogonal exocyclic aryls and planar 1‐boraphenalene units (max. deviation from the C_12_B mean plane 0.08 Å). Key metrics include short C11−C12 distances (1.350(4) Å for **6 a** and 1.348–1.356 Å for **6 c**, there are three molecules in the asymmetric unit (asu) for **6 c**) and much longer B−C1 bonds (1.540(4) Å for **6 a** and 1.533 −1.550 Å for **6 c**) and C8−C12 single bonds (1.478(4) and 1.481–1.485 Å). The short B−O distances (**6 a** 1.362(4) and **6 c** 1.351–1.369 Å) indicate π donation from the hydroxy group to boron. These distances suggest minimal endocyclic π delocalisation in the boracycle (ring C). In contrast the isoelectronic phenalenyl cations have a much smaller C−C bond distance range (1.392–1.416 Å for the ^t^Bu_3_ substituted phenalenyl cation),3a indicating significant π delocalisation throughout all rings in the all carbon analogues.

**Figure 2:**
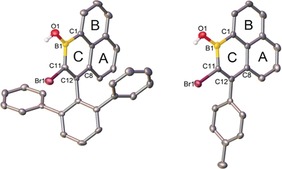
Molecular structures of **6 a** and **6 c** with ellipsoids set at 50 % probability; hydrogen atoms (except B−OH) have been omitted for clarity.^20^

The differing reactivity observed for **4 a**–**c** compared with 2‐(phenylethynyl)‐1,1′‐biphenyl (which forms **A**) presumably arises because the naphthyl moiety can intercept the vinyl carbocation in a 5‐*endo*‐dig cyclisation (step B, Scheme 3). A plausible mechanism involving tautomerisation and B−C bond cleavage can be proposed (step C) followed by a 1,2‐ migration of bromide. Lastly, an intramolecular S_E_Ar of the proximal naphthalene moiety can occur (step E) to form the six membered boracycle of the 1,2‐dibromo‐1‐boraphenalenes **5 a**–**c**. A related trapping of a vinyl cation by a proximal naphthalene during the borylative cyclisation of 1,2‐bis(1‐naphthylalkynyl)benzene with B(C_6_F_5_)_3_ has been reported.^16^

**Scheme 3:**
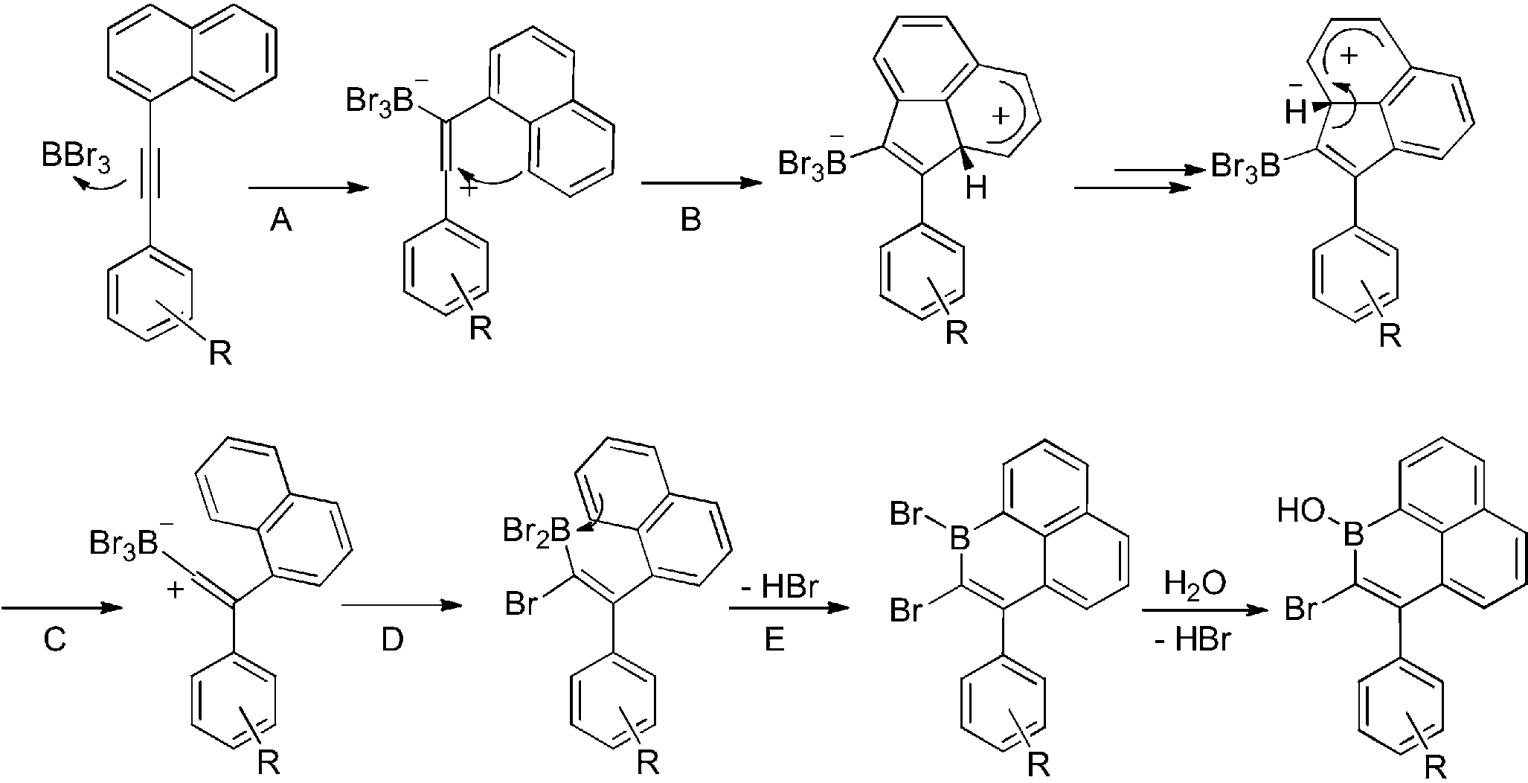
Proposed mechanism for the formation of **6 a**–**c**.

The proposed mechanism has an aryl group to stabilise the vinyl cation formed after step A. Replacement of this aryl with an alkyl or hydrogen would disfavour the formation of this vinyl cation. Therefore we anticipated that reaction of the terminal alkyne, 1‐ethynylnaphthalene, **7 a**, with more than 1 equiv BBr_3_ would instead result in *trans*‐haloboration^17^ to form **8** (Scheme 4) which positions a vinylBBr_2_ group for intramolecular S_E_Ar (akin to step E, Scheme 3) to form 1,3‐dibromo‐1‐boraphenalene (**9**). Thus **7 a** and excess BBr_3_ were combined and NMR spectroscopy indicated the quantitative formation of the haloborated product **8** within minutes of BBr_3_ addition (δ_11B_=49.9 ppm). In solution, **8** slowly transforms to **9** over 48 h at 20 °C. Compound **9** forms quantitatively (by in situ NMR spectroscopy), and crystallises from the *o*‐DCB solvent during the reaction. The solid‐state structure of **9** (Scheme 4, bottom right) has positional disorder of B1 and C2, and a mirror plane along the C1‐C4‐C5 axis, precluding detailed discussion of any metrics.

**Scheme 4:**
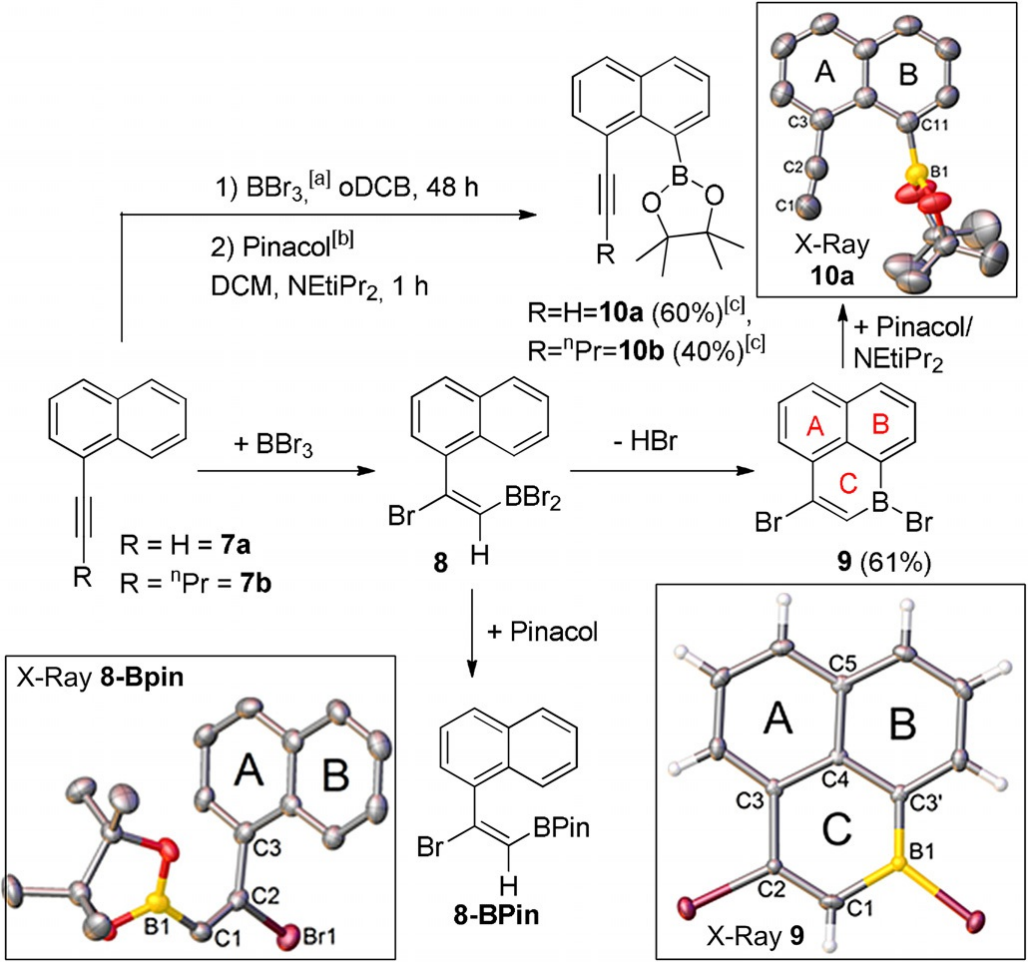
The formation of compounds **8**, **9** and **10 x**. [a] 1.2 equiv BBr_3_ for **8 a**. [b] 1.4 equiv for **8 a**. [c] Yield of isolated product. Inset: Molecular structures of **8‐BPin**, **9**, and **10 a** with ellipsoids set at 50 % probability. Hydrogen atoms have been omitted for clarity for **8‐BPin** and **10 a**.^20^

Notably, on exposure to wet solvent, the borinic acid derived from **9** is not observed; instead protodeboronation occurs. This can be used to transform **9** into **10 a** by addition of Hünigs base/pinacol (Scheme 4, top right), with **10 a** forming via protodeboronation and an E2 elimination from the haloalkene. Comparable reactivity was observed for 1‐(pent‐1‐yn‐1‐yl)naphthalene (**7 b**) to furnish **10 b**. The identification of **10 a** was confirmed by X‐ray diffraction studies, which revealed distorted C1‐C2‐C3 angles (174.1(3)°), C2‐C3‐CtA (where CtA=centroid of ring A, 175.60(17)°) and B1‐C11‐CtB (168.33(16)°). **10 a/b** can be synthesised directly from **7 a/b** with no isolation of intermediates, and are the first reported 8‐borylated‐1‐alkynyl naphthalenes to the best of our knowledge. To confirm the formation of **9** proceeds via **8**, a solution of pinacol was added after 20 minutes to the mixture derived from **7 a**/BBr_3_ to form **8‐BPin**. This led to quantitative conversion to **8‐BPin** but it was isolated in only 30 % yield by crystallisation (**8‐BPin** decomposes under basic conditions to furnish **7 a** and was unstable on silica).

1‐Boraphenalene derivatives that are bench stable and contain exocyclic boron substituents that do not π donate to boron were next targeted. **6 c** reacts with MesMgBr to form two species in a circa 9:1 ratio (Scheme 5, top). The major product (**11**) is functionalised at carbon, leaving the borinic acid moiety intact, as indicated by a resonance at 6.04 ppm in the ^1^H NMR spectrum for the B−OH. The minor product (**12**) is functionalised at boron, leaving the vinyl bromide group intact. In contrast, functionalisation of the bromo congener **5 c** with MesMgBr results predominantly in the formation of **12** along with minor unidentified species, with the formation of **11** not observed. **12** is bench stable and can be isolated in 55 % yield with a δ_11B_ of 58.0 ppm. Increasing the ratio of MesMgBr: **5 c**/**6 c** to more than 10:1 did not result in any significant double arylation of either compound at 20 °C or at raised temperatures. Furthermore, the reaction of **12** in a sealed tube with excess MesMgBr at 100 °C resulted in the formation of the di‐arylated compound **13** as only the minor product with **14** the major product (Scheme 5, middle). The formation of **14** presumably occurs by Grignard metathesis generating the Grignard reagent derived from **12** which upon aqueous work up is hydrolysed to **14**. As observed for **5 c**, **9** can be readily functionalised at boron, but in this case superior yields were obtained using ZnMes_2_, which afforded bench stable **15** in 88 % yield. Despite repeated attempts, only compound **12** was amenable to crystallisation in our hands. The solid‐state structure of **12** revealed a planar 1‐boraphenalene unit and effectively orthogonal aryl groups. The bond metrics in the boracycle were closely comparable to those found in **6 a** and **6 c**, including a short C11−C12 distance of 1.360(4) Å.

**Scheme 5:**
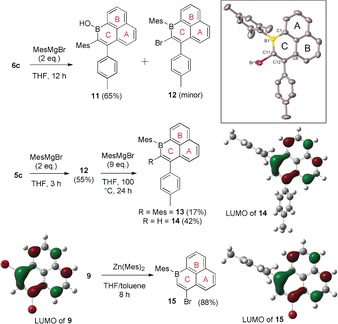
Functionalization of **5 c** and **6 c**,. Top right: solid‐state structure of **12** (hydrogens omitted for clarity). Middle right: the LUMO for **14** (isovalue=0.05, the LUMO for **11**, **12**, and **13** are closely comparable). Bottom: the formation of **15** from **9** and the LUMO of **9** and **15** (isovalue=0.05).

Compounds **5 c**, **9**, and **12**–**15** were calculated at the B3LYP/6–311G(d,p) level. All six calculated structures have C=C distances for the C1−C2 unit (numbering as per Scheme 4, bottom right) between 1.35–1.37 Å comparable to those found in the solid‐state structures of **6 a, 6 c**, and **12**. The C_12_B cores are planar in each case with the Mes moieties oriented effectively orthogonal in each compound. Most notably, the LUMO for each compound is effectively identical (Supporting Information, Figure S7), being located on the C_12_B core and being predominantly non‐bonding in nature, with zero orbital coefficients on exocyclic groups in contrast to the LUMO of isoelectronic **3**. Thus the replacement of {C−H}^+^ in **1^+^** for {B−R} has minimal effect on the nature of this frontier orbital. This is notable as the non‐bonding character of this frontier orbital is crucial for the unique redox‐properties of phenalenyls.^1^ In contrast to the LUMO, the occupied π orbitals are distinct for the 1‐boraphenalenes compared to **1^+^**. For **5 c** and **9** the HOMO is principally located on rings A and C (Scheme 5; Supporting Information, Figure S7) with some contribution from the exocyclic bromines. For the B‐Mes substituted compounds the HOMO and HOMO−1 are both located on the mesityl group (Supporting Information, Figure S7), but the occupied π orbitals on the C_12_B core are also more localized than in in **1^+^** where the highest energy occupied π orbitals are delocalized throughout the phenalenyl C_13_ core (Supporting Information, Figure S6).

Nucleus‐independent chemical shifts (NICS) were determined for the reported compounds, the perprotio 1‐boraphenalene (C_12_BH_9_) and the isoelectronic analogue **1^+^** (Supporting Information, Table S3). For all the 1‐boraphenalenes the boracycle (ring C) is effectively non‐aromatic (NICS(1) values between −0.3 and −1.6) while the naphthyl unit has significant aromaticity (NICS(1) values for rings A and B are between −10.4 and −9.6). This is distinct to the more symmetric aromatic structure of *D*_3*h*_ **1^+^** (NICS(1) −7.8) and to **3** (which is dominated by a single highly aromatic Clar sextet). Therefore while the LUMO of the 1‐boraphenalenes and **1^+^** are closely comparable in character, the overall π electronic structures are different owing to the lower symmetry on incorporating boron and the higher energy of the B p_π_ orbital relative to the C p_π_ orbitals (Supporting Information, Figures S6, S7). To estimate the effect of this on the aromatic stabilization of the C_5_B ring in 1‐boraphenalenes relative to isoelectronic carbocations the isomerization method was used (calculations at the B3LYP/6–311G(d,p) level, Eq. (1) and (2) in Scheme 6).^18^ This revealed that this 1‐boraphenalene has a much lower aromatic stabilization energy than the isoelectronic carbocation congener. The lower aromatic stabilization energy for the 1‐boraphenalenes was supported by an isodesmic reaction (Eq. (3) in Scheme 6),^19^ which confirmed the greater aromatic stability of phenalenyl cations (>12 kcal mol^−1^). While it has been demonstrated numerous times that the LUMOs of B doped PAHs and carbocation analogues are often similar in nature,^6^ to fully understand the properties of these isoelectronic pairs consideration of the occupied π orbitals is also essential.

**Scheme 6:**
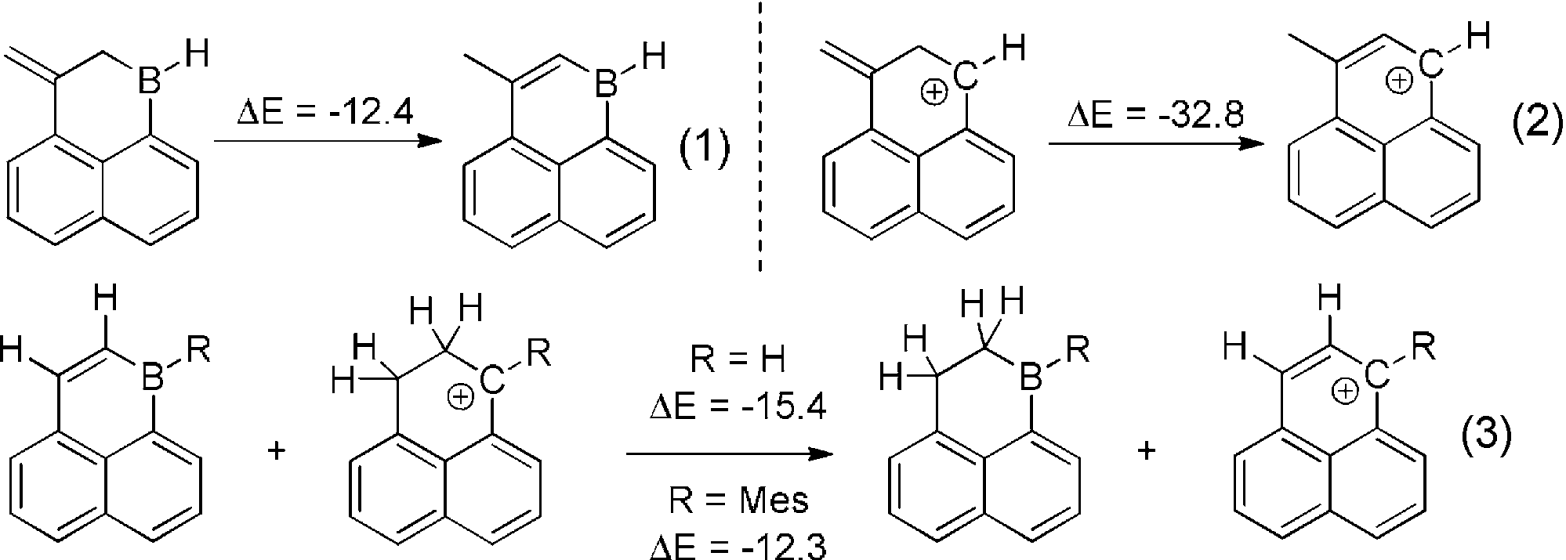
Electronic energies (kcal mol^−1^) for a range of isodesmic reactions.

With an understanding of the electronic structure of **12**–**15** in hand, the propensity of these to undergo redox was investigated. The first reduction wave is reversible (Table 1; Supporting Information, Figures S2–S5) and its potential mirrors the trend observed computationally with **12** and **15** containing one inductively withdrawing bromine substituent having a less negative reduction potential than **13** and **14**. For **12**–**14**, the second reduction event is significantly more negative than the first. This separation and the reversible nature of the first reduction wave indicates that 13 π electron radical anions should be accessible. However, attempts to date to chemically reduce **13** and **14** have led to either complex diamagnetic mixtures (with **14**) or NMR silent product(s) that have frustrated isolation (with **13**).

**Table 1:** Reduction potentials for 1‐boraphenalenes.^[a]^

	First reduction			Second reduction
	*E*_peak_ [V]	*E*_1/2_ [V]	LUMO [eV]	[V]^[b]^
**12**	−1.84	−1.75	−3.11	−2.71
**13**	−1.95	−1.89	−2.97	−2.72
**14**	−1.95	−1.89	−2.98	−2.68
**15**	−1.85	−1.74	−3.13	−2.11

[a] Measured in THF (1 mm) with [nBu_4_N][PF_6_] (0.1 m) as the supporting electrolyte at a scan rate of 50 mV s^−1^. Potentials are given relative to the Fc/Fc^+^ redox couple. LUMO energies from onset of reduction with the Fc/Fc^+^ redox couple which is taken to be 4.80 eV below vacuum. [b] Value at peak current.

In conclusion, the first boron‐only doped phenalenes are reported, that are available in one step from commercially available precursors (for **9**), or in two steps in all other cases. These can be selectively functionalized to provide compounds possessing good bench stability. Notably, the nature of the LUMO in these 1‐boraphenalenes is closely comparable to that in the extensively studied all carbon phenalenyl cation analogues. However, the 1‐boraphenalenes have significantly lower aromatic stabilization of the C_5_B ring than observed in each ring in the *D*_3*h*_ phenalenyl cations due to the less delocalized nature of the occupied orbitals of π symmetry in the 1‐boraphenalenes. For the boraphenalenes containing B−Mes, a reversible reduction wave is observed well separated from the second reduction process, indicating that the 13 π‐electron radical anion, analogous to the phenalenyl radical, is accessible. Further studies into generating 1‐boraphenalenenes, particularly examples enabling access to isolable 13 π‐electron radical anions, are currently ongoing.

## Conflict of interest

The authors declare no conflict of interest.

## Supporting information

As a service to our authors and readers, this journal provides supporting information supplied by the authors. Such materials are peer reviewed and may be re‐organized for online delivery, but are not copy‐edited or typeset. Technical support issues arising from supporting information (other than missing files) should be addressed to the authors.

Supplementary
