# Supplementary material for: Synthesis, Characterization, and Functionalization of 1‐Boraphenalenes
Source: Angew Chem Int Ed Engl. 2018 Jun 6;57(27):8084–8. doi: 10.1002/anie.201803180 (PMC6175385; doi:10.1002/anie.201803180)
Supplement: Supplementary file 1 — Supplementary [file ANIE-57-8084-s001.pdf]

Angewandte Chemie
Eine Zeitschrift der Gesellschaft Deutscher Chemiker

Supporting Information

Synthesis, Characterization, and Functionalization of 1-Boraphenalenes

*Rachel J. Kahan, Daniel L. Crossley, Jessica Cid, James E. Radcliffe, and Michael J. Ingleson**

anie_201803180_sm_miscellaneous_information.pdf

Table of Contents

General Considerations S2

Synthetic Procedures S3

NMR Spectra S13

Crystallographic Details and extended Packing Structures S39

Electrochemical Properties S41

DFT Calculations S44

Optimised Structure Coordinates S46

References S66

General Considerations

Unless otherwise stated, all manipulations were carried out using standard Schlenk techniques under argon, or in a MBraun UniLab glovebox, under an atmosphere of argon (< 0.1 ppm O2/H_2O). All solvents were distilled from appropriate drying agents: THF (potassium); toluene (potassium); hexane (NaK); triethylamine (CaH_2); and *ortho*-dichlorobenzene (CaH_2). All solvents were stored over 3 Å molecular sieves with the exception of toluene and hexane, which were stored over a potassium mirror. Unless otherwise stated all compounds were purchased from commercial sources and used as received. Solvents for column chromatography were of technical grade and used without further purification. Column chromatography was performed on silica gel (230-400 mesh).

NMR spectra were recorded on Bruker AvanceIII-400, Bruker AvanceII-500 or Bruker Ascend-400 spectrometers. Chemical shifts are reported as dimensionless δ values and are frequency referenced relative to residual protio impurities in the NMR solvents for ^{1}H and 13C{^{1}H}, while ^{11}B shifts are referenced relative to external BF_3-etherate. Coupling constants J are given in Hertz (Hz) as positive values regardless of their real individual signs. The multiplicity of the signals are indicated as "s", "d", "t", "q" "pent", "sept" or "m" for singlet, doublet, triplet, quartet, pentet, septet or multiplet, respectively. Broad features in the ^{11}B NMR spectra are due to boron present in borosilicate glass. Carbon atoms directly bonded to boron are not always observed in the $^{13}C\{^{1}H\}$ NMR spectra due to quadrupolar relaxation leading to signal broadening.

High resolution mass spectra (HRMS) were recorded on a Waters QTOF mass spectrometer. GCMS analysis was performed on an Agilent Technologies 7890A GC system equipped with an Agilent Technologies 5975C inertXL EI/CI MSD with triple axis detector. The column employed was an Agilent J&W HP-5ms ((5%-Phenyl)-methylpolysiloxane) of dimensions: length, 30 m; internal diameter, 0.250 mm; film, 0.25 µm. Microanalysis was performed by Mr Martin Jennings at the University of Manchester microanalytical service or by Mr Stephen Boyer at the London Metropolitan University elemental analysis service.

Cyclic voltammetry was performed using a CH-Instrument 1110C Electrochemical/Analyzer potentiostat under a nitrogen flow. Measurements were made using a 1 mM analyte solution with 0.1 M tetra^{n}butylammonium hexafluorophosphate (Fluka ≥99.0 %) as the supporting electrolyte in THF that had been dried and degassed prior to use and obtained from a dry solvent system. A glassy carbon electrode served as the working electrode and a platinum wire as the counter electrode. An Ag/$AgNO_3$ non-aqueous reference electrode was used. All scans were calibrated against the ferrocene/ferrocenium (Fc/Fc^+) redox couple, which in this work is taken to be 4.8 eV below vacuum.[1] The half-wave potential of the ferrocene/ferrocenium (Fc/Fc^+) redox couple ($E_{1/2,\ Fc,Fc+}$) was estimated from $E_{1/2,\ Fc,Fc+} = (E_{ap} + E_{cp})/2$, where E_{ap} and E_{cp} are the anodic and cathodic peak potentials, respectively.

Calculations were performed using the Gaussian09 suite of programmes.[2] Optimisations and NICS calculations were performed at the B3LYP/6-311G(d,p) level.[3] In all cases, structures were confirmed as minima by frequency analysis and the absence of imaginary frequencies. Full Cartesian coordinates are provided below.

Synthetic Procedures

General preparation for alkynes 4a – 4c and 7b

To a mixture of aryl bromide (1 eq.), alkyne (>1 eq.) and copper(I) iodide (0.0x eq.) was added dry THF and dry triethylamine. Under argon flow was added $Pd(PPh_3)_2Cl_2$ (0.0x eq.) The mixture forms a black solution within 15 minutes which heated to 60 °C for 48 hours. Upon cooling, the mixture was filtered through a plug of silica and the remaining solids were washed with petroleum ether. The washings were combined and dried *in vacuo*. The product was purified by flash column chromatography using a DCM:petrol eluent to yield the corresponding alkyne.

Synthesis of 1-([1,1':3',1''-terphenyl]-2'-ylethynyl)naphthalene (**4a**)

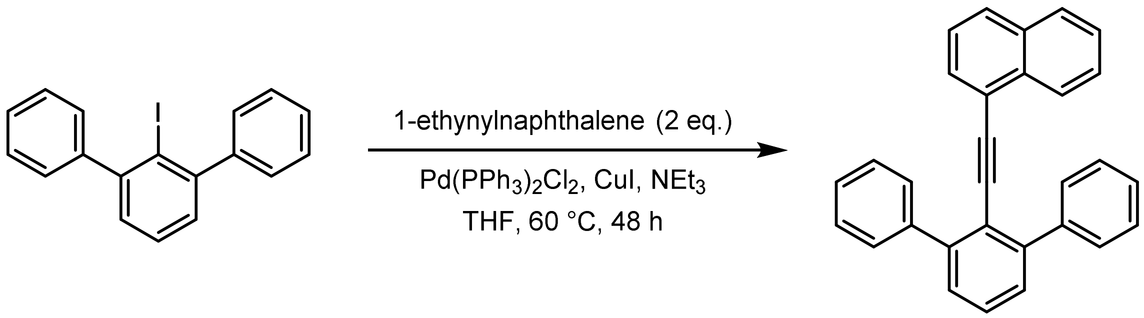

Prepared according to the general procedure. 2'-iodo-1,1':3',1''-terphenyl (0.500 g, 1.40 mmol, 1 eq.), 1-ethynylnaphthalene (0.447 g, 2.94 mmol, 2.1 eq.), copper(I) iodide (6 mg, 3.1×10^{-5} mol, 0.02 eq.), $Pd(PPh_3)_2Cl_2$ (29 mg, 4.0×10^{-5} mol, 0.03 eq.), THF (25 mL) and triethylamine (10 mL). The crude residue contains a mixture of **4a** and 1,4-di(naphthalen-1-yl)buta-1,3-diyne. The mixture was purified by flash column chromatography using a DCM:petrol eluent (1:6) to yield 1,4-di(naphthalen-1-yl)buta-1,3-diyne at Rf = 0.59 and the product at Rf = 0.43 (0.370 g, 69%).

^{1}H NMR (500MHz, CD_2Cl_2) δ = 7.76 (m, 1 H), 7.74 - 7.72 (m, 5 H), 7.55 - 7.41 (m, 10 H), 7.31 (dd, J = 8.2, 7.2 Hz, 1 H), 7.28 - 7.23 (m, 2 H), 7.18 (dd, J = 6.9, 1.3 Hz, 1 H). ^{13}C{^{1}H} NMR (126MHz, CD_2Cl_2) δ = 145.8, 142.0, 133.6, 133.5, 130.5, 130.3, 129.1, 129.0, 128.8, 128.7, 128.4, 128.1, 127.0, 126.9, 126.8, 125.7, 121.7, 121.2, 94.6, 94.3. HRMS (APCI) Calculated for $C_{30}H_{21}^+$ ($[M+H]^+$) 381.1643. Found 381.1633.

Synthesis of 1-([1,1′-biphenyl]-2-ylethynyl)naphthalene (**4b**)

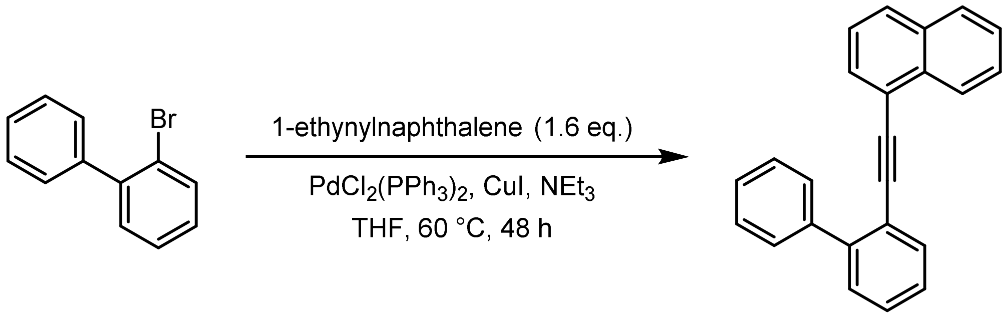

Prepared according to the general procedure. 2-bromobiphenyl (1.059 g, 4.54 mmol, 1 eq.), 1-ethynylnaphthalene (1.1256 g, 7.40 mmol, 1.6 eq.), copper(I) iodide (6.8 mg, 3.6×10^{-5} mol, 0.01 eq.), $Pd(PPh_3)_2Cl_2$ (53.5 mg, 7.6×10^{-5} mol, 0.02 eq.), THF (25 mL) and triethylamine (10 mL). The crude residue contains both starting materials. The mixture was purified by flash column chromatography using a DCM:petrol eluent (1:9) to yield the product **4b** at Rf = 0.19 (0.1961 g, 0.644 mmol, 14%).

^{1}H NMR (500MHz, CD_2Cl_2) δ = 7.88 – 7.76 (m, 4H), 7.72 – 7.67 (m, 2H), 7.65 – 7.60 (m, 1H), 7.53 – 7.44 (m, 6H), 7.44 – 7.38 (m, 3H). ^{13}C{^{1}H} NMR (126MHz, CD_2Cl_2) δ = 144.7, 141.5, 133.7, 133.6, 133.5, 130.8, 130.1, 130.0, 129.2, 129.2, 128.8, 128.6, 128.1, 127.8, 127.2, 126.9, 126.8, 125.8, 122.4, 121.5, 94.6, 90.9. HRMS (APCI) Calculated for $C_{24}H_{17}^+$ ($[M+H]^+$) 305.1325. Found 305.1323. The data for this compound is comparable to that previously reported.[4,5]

Synthesis of (1-(p-tolylethynyl)naphthalene) (**4c**)

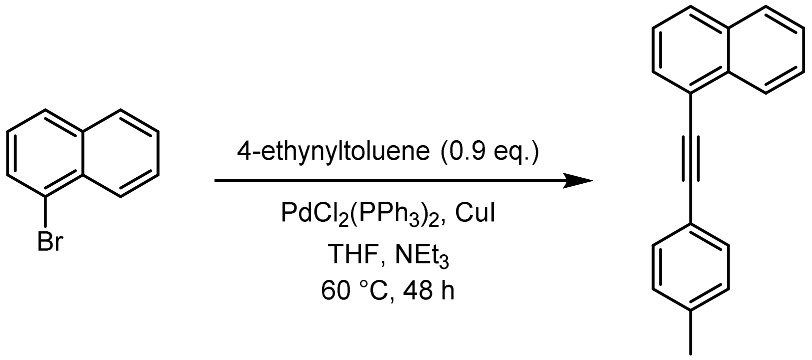

Prepared according to the general procedure. 1-bromonaphthalene (1.093 g, 5.46 mmol, 1 eq.), 4-ethynyltoluene (0.576 g, 4.96 mmol, 0.9 eq.), copper(I) iodide (10 mg, 5.2×10^{-5} mol, 0.01 eq.), $Pd(PPh_3)_2Cl_2$ (71 mg, 1.0×10^{-4} mol, 0.02 eq.), THF (25 mL) and triethylamine (10 mL). Analytically pure product **4c** eluted from a column (1:7 then 1:5 DCM:petrol) at Rf = 0.47 (0.832 g, 3.43 mmol, 68%).

^{1}H NMR (400MHz, CD_2Cl_2) δ = 8.52 (dd, J = 8.4, 1.1 Hz, 1H), 7.93 (d, 8.1 Hz, 1H), 7.89 (d, 8.3 Hz, 1H), 7.82 (dd, 7.1, 1.2 Hz, 1H), 7.66 (ddd, J = 8.3, 6.9, 1.4 Hz, 1H), 7.61 (m, 3H), 7.51 (dd, J = 8.3, 7.2 Hz, 1H), 7.27 (d, J = 7.9 Hz, 2H), 2.43 (s, 3H). ^{13}C{^{1}H} NMR (101MHz, CD_2Cl_2) δ = 139.4, 133.8, 133.7, 132.0, 130.8, 129.8, 129.2, 128.9, 127.4, 127.0, 126.7, 125.9, 121.6, 120.8, 95.1, 87.3, 21.8. HRMS (APCI) Calculated for $C_{19}H_{14}^+$ (M^+) 242.1090. Found 242.1082. The data for this compound is comparable to that previously reported.[6]

Synthesis of 1-(pent-1-yn-1-yl)naphthalene (**7b**)

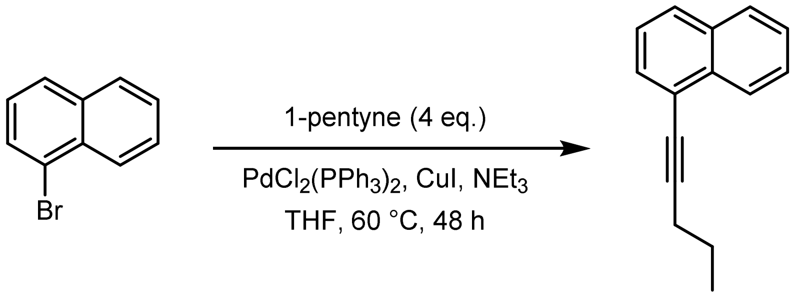

Prepared according to the general procedure. 1-bromonaphthalene (4.413 g, 20.0 mmol, 1 eq.), 1-pentyne (7.9 mL, 80.1 mmol, 4 eq.), copper(I) iodide (30 mg, 1.6 x 10^{-4} mol, 0.01 eq.), $Pd(PPh_3)_2Cl_2$ (238 mg, 3.4 x 10^{-4} mol, 0.02 eq.), THF (25 mL) and triethylamine (10 mL). Analytically pure product eluted from a column (1:9 DCM:petrol) at Rf = 0.58 (3.605 g, 18.6 mmol, 93%).

^{1}H NMR (400MHz, CD_2Cl_2) δ = 8.39 (d, J = 8.2 Hz, 1H), 7.88 (d, 7.9 Hz, 1H), 7.82 (d, 8.3 Hz, 1H), 7.65 (d, 7.1 Hz, 1H), 7.59 (m, 1H), 7.54 (ddd, J = 8.2, 6.8, 1.4 Hz, 1H), 7.43 (dd, J = 8.3, 7.1 Hz, 1H), 2.59 (t, J = 7.0 Hz, 2H), 1.77 (sept, J = 7.3 Hz, 2H), 1.17 (t, J = 7.4 Hz, 3H). ^{13}C{^{1}H} NMR (101MHz, CD_2Cl_2) δ = 134.0, 133.8, 130.5, 128.8, 128.4, 127.1, 126.8, 126.7, 125.8, 122.4, 96.1, 79.0, 23.0, 22.2, 14.0. HRMS (APCI) Calculated for $C_{15}H_{14}^+$ (M^+) 194.1090. Found 194.1091. The data for this compound is comparable to that previously reported.[7]

Synthesis of 3-([1,1':3',1''-terphenyl]-2'-yl)-2-bromo-1H-naphtho[1,8-bc]borinin-1-ol (**6a**)

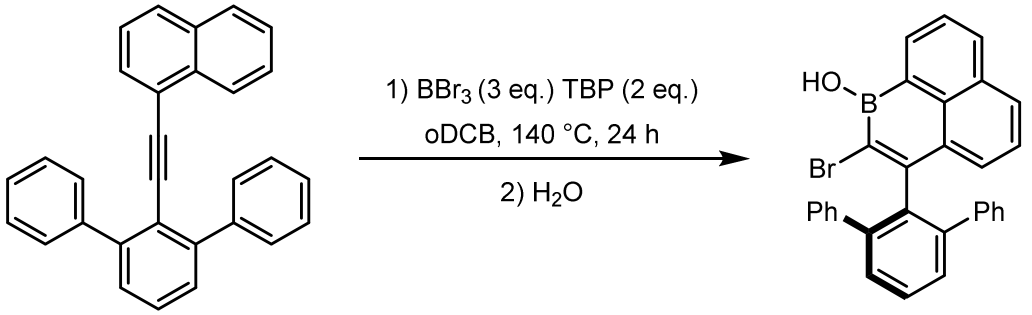

To a J. Young's valve topped NMR tube equipped with a DMSO-d_6 capillary was added under argon flow 2-(naphthalene-1-ylethynyl)-1,3-terphenyl (23 mg, 6.1 x 10^{-5} mol, 1 eq.), 2,4,6-tri-*tert*-butylpyridine (26 mg, 1.1 x 10^{-4} mol, 1.8 eq.), dry *ortho*-dichlorobenzene (0.4 mL) and BBr_3 (0.16 mL, 1.0 M in hexanes, 1.6 x 10^{-4} mol, 2.6 eq.). The mixture was heated to 140 °C for 24 hours. Upon cooling, all volatiles were removed *in vacuo*. The brown residue containing **5a** was dissolved in 2 mL DCM and washed with 1.0 M HCl then water and dried over $MgSO_4$. The yellow solution was filtered then purified by preparative TLC using a DCM:petrol eluent (1:2) to yield analytically pure product **6a** at Rf = 0.27 as a yellow powder (19 mg, 3.9 x 10^{-5} mol, 64%. This materials proved to be poorly soluble on purification in common organic solvents.

Elemental Analysis Expected C 73.96% H 4.14%, Found C 72.32% H 4.13%. ^{1}H NMR (400MHz, CD_2Cl_2) δ = 8.41 (dd, 6.9, 1.4 Hz, 1H), 8.06 (dd, 8.2, 1.4 Hz, 1H), 7.86 (dd, 8.1, 1.2 Hz, 1H), 7.63 (m, 2H), 7.51 (m, 3H), 7.37 (dd, 7.7, 7.7 Hz, 1H), 7.23 (m, 4H), 7.03 (m, 6H), 5.75 (broad, 1H, B–OH). ^{11}B NMR (128MHz, CD_2Cl_2) δ = 37.8 ($\nu_{½}$ = 946 Hz). ^{13}C{^{1}H} NMR (101MHz, CD_2Cl_2) δ = 157.0, 141.4, 141.0, 136.9, 135.8, 134.3, 133.4, 132.2, 132.0, 131.6, 131.2, 129.6, 128.8, 128.3, 127.4, 126.8, 125.8, 125.3.

Synthesis of 3-([1,1'-biphenyl]-2-yl)-2-bromo-1H-naphtho[1,8-bc]borinin-1-ol (**6b**) and 4,4,5,5-tetramethyl-2-(10-(naphthalen-1-yl)phenanthren-9-yl)-1,3,2-dioxaborolane (Compound **C**)

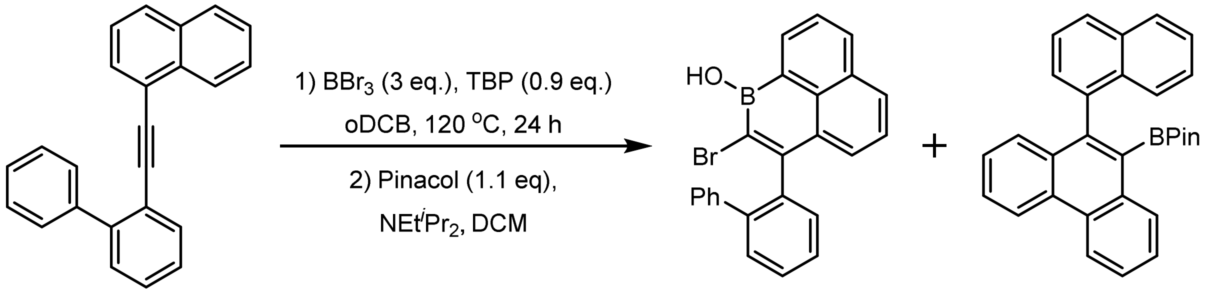

To an ampoule under argon flow was added 1-([1,1'-biphenyl]-2-ylethynyl)naphthalene (0.162 g, 0.540 mmol, 1 eq.), 2,4,6-tri-*tert*-butylpyridine (0.124 g, 0.503 mmol, 0.98 eq.) and dry *ortho*-dichlorobenzene (2 mL). To this was added BBr_3 (1.50 mL, 1.0 M in hexanes, 1.50 mmol, 2.8 eq.) to form a purple suspension. The mixture was heated to 120 °C for 24 hours. Upon cooling, all volatiles were removed *in vacuo*. The residue containing **5b** was dissolved in DCM (3 mL) to give a dark green solution and to this was added dropwise a solution of pinacol (72 mg, 0.61 mmol, 1.1 eq.) in DCM (3 mL) and Hünig's base (0.5 mL) to form a red/brown suspension. All volatiles were removed *in vacuo* to give a mixture of **6b** and Compound **C** (in equal ratio by ^{1}H NMR) and a small amount of the protodeboronated compound 9-(naphthalene-1-yl)phenanthrene (by comparison to the literature).[4] The mixture was purified by flash column chromatography using a DCM:petrol eluent (1:9 then 1:1) to yield 9-(naphthalene-1-yl)phenanthrene at Rf = 1.0, Compound **C** at Rf = 0.38 (61.8 mg, 1.44 x 10^{-4} mol, 27%) and **6b** at Rf = 0.32. (5.5 mg, 1.09 x 10^{-5} mol, 2%). The yield of 6b is low as it was found to decompose during column chromatography.

6b: ^{1}H NMR (400MHz, CD_2Cl_2) δ = 8.50 (dd, 6.9, 1.4 Hz, 1H), 8.15 (dd, 8.1, 1.4 Hz, 1H), 7.94 (dd, 6.7, 2.6 Hz, 1H), 7.70 (dd, J = 8.1, 6.9 Hz, 1H), 7.60 – 7.47 (m, 3H), 7.40 – 7.34 (m, 2H), 7.31 – 7.25 (m, 3H), 7.10 – 7.04 (m, 3H), 5.97 (s, br, 1H, B–OH).

Compound **C**: ^{1}H NMR (400MHz, CD_2Cl_2) δ = 8.84 – 8.78 (m, 2H), 8.03 (dd, 8.1, 1.5 Hz, 1H), 7.96 (m, 2H), 7.72 (ddd, 8.3, 6.9, 1.5 Hz, 1H), 7.68 – 7.58 (m, 3H), 7.52 (dd, 6.9, 1.3 Hz, 1H), 7.46 (ddd, 8.2, 6.6, 1.4 Hz, 1H), 7.37 – 7.31 (m, 2H), 7.29 – 7.23 (m, 2H), 0.95 (s, 6H), 0.79 (s, 6H). Due to hindered rotation on the NMR timescale the pinacol methyl resonances are inequivalent. ^{11}B NMR (128MHz, CD_2Cl_2) δ = 31.6 ($\nu_{½}$ = 567 Hz). $^{13}C\{^1H\}$ NMR (101MHz, CD_2Cl_2) δ = 142.6, 139.1, 134.1, 134.1, 134.1, 132.3, 131.2, 130.1, 129.3, 129.3, 128.5, 128.4, 128.0, 127.4, 127.4, 127.4, 127.0, 127.0, 126.4, 126.3, 125.8, 123.4, 123.1, 84.2, 25.0, 24.7. HRMS (APCI) Calculated for $C_{30}H_{28}BO_2^+$ ($[M+H]^+$) 431.2177. Found 431.2184.

Synthesis of 2-bromo-3-(p-tolyl)-1H-naphtho[1,8-bc]borinin-1-ol (**6c**)

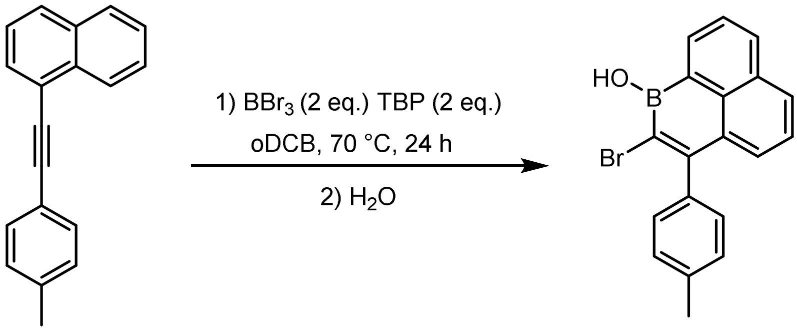

To an ampoule under argon flow was added (1-(p-tolylethynyl)naphthalene) (0.144 g, 0.593 mmol, 1 eq.), 2,4,6-tri-*tert*-butylpyridine (0.338 g, 1.37 mmol, 2 eq.) and dry *ortho*-dichlorobenzene (2 mL). To this was added BBr_3 (1.20 mL, 1.0 M in hexanes, 1.20 mmol) and a darkening of the solution ensued. The mixture was heated to 70 °C for 24 hours. Upon cooling, all volatiles were removed *in vacuo*. The brown residue containing **5c** was dissolved in 5 mL DCM and washed with 1.0 M HCl then water and dried over $MgSO_4$. The yellow solution was filtered then purified by flash column chromatography using a DCM:petrol eluent (1:1) to yield analytically pure product **6c** at Rf = 0.32 as a yellow powder (0.130 g, 3.73 x 10^{-4} mol, 63%).

Elemental Analysis Expected C 65.38% H 4.04%, Found C 65.21% H 4.01%. ^{1}H NMR (400MHz, CD_2Cl_2) δ = 8.59 (dd, 6.9, 1.4 Hz, 1H), 8.22 (dd, 8.1, 1.4 Hz, 1H), 8.0 (dd, 8.0, 1.3 Hz, 1H), 7.76 (dd, J = 8.1, 6.9 Hz, 1H), 7.41 (dd, 7.7, 7.7 Hz, 1H), 7.38 – 7.33 (m, 3H), 7.19 (d, 8.0 Hz, 2H), 6.11 (s, 1H), 2.49 (s, 3H). ^{11}B NMR (128MHz, CD_2Cl_2) δ = 37.4 ($\nu_{½}$ = 565 Hz). ^{13}C{^{1}H} NMR (101MHz, CD_2Cl_2) δ = 159.3, 138.3, 138.3, 136.7, 135.1, 134.0, 132.9, 132.8, 132.4, 132.1, 129.4, 129.4, 126.8, 126.1, 21.7.

(**E**)-2-(2-bromo-2-(naphthalen-1-yl)vinyl)-4,4,5,5-tetramethyl-1,3,2-dioxaborolane (**8-BPin**)

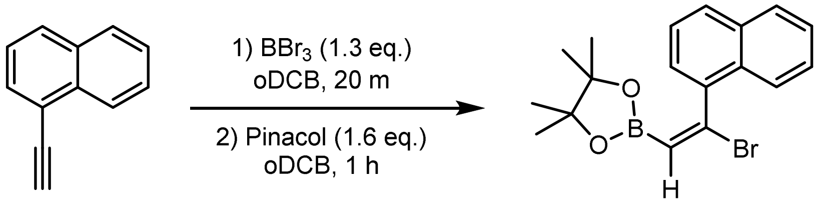

To an ampoule containing a solution of 1-ethynylnaphthalene (**7a**) (0.146 g, 0.96 mmol, 1 eq.) in *ortho*-dichlorobenzene (1 mL) was added dropwise a solution of boron tribromide (1.25 mL, 1.0 M in hexanes, 1.25 mmol, 1.3 eq.). The mixture instantly develops a deep blue colour and was stirred for 20 minutes. To the mixture containing **8** was added a solution of pinacol (0.183 g, 1.55 mmol) in dichloromethane (1 mL) dropwise at ambient temperature and a deep red colour develops. The mixture was stirred for 1 hour then all volatiles were removed *in vacuo*. The product was extracted in hexane and filtered to give a brown solution from which a brown oil and colourless crystals were obtained (crude yield: 0.259 g). Analytically pure product **8-BPin** was obtained by recrystallization of the mixture from hexane to give colourless crystals (0.104 g, 0.290 mmol, 30%).

^{1}H NMR (400MHz, CD_2Cl_2) δ = 8.09 (m, 1H), 7.89 – 7.82 (m, 2H), 7.54 (ddd, 8.4, 6.8, 1.6 Hz, 1H), 7.49 (ddd, 8.0, 7.1, 1.7 Hz, 1H), 7.44 – 7.41 (m, 2H), 6.5 (s, 1H), 0.9 (s, 6H), 0.82 (s, 6H). Due to hindered rotation on the NMR timescale the pinacol methyl resonances are inequivalent. ^{11}B NMR (128MHz, CD_2Cl_2) δ = 28.6 ($\nu_{½}$ = 286 Hz). ^{13}C{^{1}H} NMR (101MHz, CD_2Cl_2) δ = 139.6, 138.2, 134.0, `130.8, 129.6, 128.7, 126.8, 126.8, 126.6, 126.0, 125.4, 83.9, 24.7, 24.5. HRMS (APCI) Calculated for $C_{18}H_{21}BBrO_2^{+}$ ($[M+H]^{+}$) 359.0812. Found 359.0801.

Synthesis of 1,3-dibromo-1H-naphtho[1,8-*bc*]borinine (**9**)

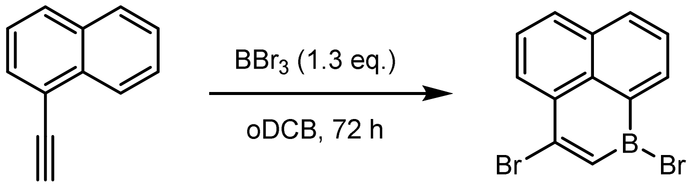

To an ampoule containing a solution of 1-ethynylnaphthalene (0.305 g, 2.00 mmol, 1 eq.) in *ortho*-dichlorobenzene (3 mL) was added dropwise a solution of boron tribromide (2.6 mL, 1.0 M in hexanes, 2.6 mmol, 1.3 eq.). The mixture instantly develops a deep blue colour and was stirred for 20 minutes. On standing **9** (0.395 g, 1.23 mmol, 61%) spontaneously crystallised from the crude reaction mixture over three days.

Elemental Analysis Expected C 44.79% H 2.19%, Found C 44.90% H 2.24%. ^{1}H NMR (400MHz, CD_2Cl_2) δ = 8.78 (dd, 7.0, 1.5 Hz, 1H), 8.61 (dd, 7.5, 1.2 Hz, 1H), 8.34 (dd, 8.1, 1.5 Hz, 1H), 8.18 (dd, 8.2, 1.2 Hz, 1H), 7.81 (dd, 8.1, 7.0 Hz, 1H), 7.71 (dd, 8.1, 7.5 Hz, 1H), 7.62 (s, 1H). ^{11}B NMR (128MHz, CD_2Cl_2) δ = 52.0 ($\nu_{½}$ = 311 Hz). $^{13}C\{^{1}H\}$ NMR (101MHz, CD_2Cl_2) δ = 147.8, 143.8, 137.9, 137.1, 135.2, 132.4, 131.6, 131.4, 127.5, 126.9. HRMS (APCI) Calculated for $C_{12}H_7BBr_2H_2O^+$ (M+H_2O) 336.9040. Found 336.9047.

Synthesis of 2-(8-ethynylnaphthalen-1-yl)-4,4,5,5-tetramethyl-1,3,2-dioxaborolane (**10a**)

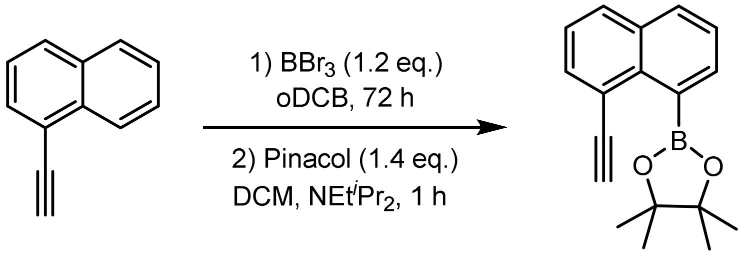

To an ampoule containing a solution of 1-ethynylnaphthalene (0.155 g, 1.02 mmol, 1 eq.) in *ortho*-dichlorobenzene (1.0 mL) was added dropwise a solution of boron tribromide (1.25 mL, 1.0 M in hexanes, 1.25 mmol). The mixture instantly develops a deep blue colour and was stirred for 3 days. All volatiles were removed *in vacuo* then the residue (containing **9**) was taken up in dichloromethane (4 mL). To this was added dropwise a solution of pinacol (0.166 g, 1.4 mmol, 1.4 eq.) and Hünig's base (0.5 mL) in dichloromethane (3 mL) at ambient temperature. The mixture develops a dark brown colour and was stirred for 1 hour. All volatiles were removed *in vacuo* and the product was extracted in pentane and filtered. **10a** was purified by filtration through a 2 inch silica plug in DCM to give a yellow solution. Crystals (0.170 g, 0.612 mmol, 60%) were obtained from slow evaporation of the solvent.

^{1}H NMR (400MHz, CD_2Cl_2) δ = 7.90 (dd, 8.1, 1.4 Hz, 1H), 7.87 (dd, 8.2, 1.3 Hz, 1H), 7.81 (dd, 7.2, 1.3 Hz, 1H), 7.74 (dd, 6.9, 1.3 Hz, 1H), 7.50 (dd, 8.2, 6.9 Hz, 1H), 7.44 (dd, 8.2, 7.2 Hz, 1H), 3.52 (s, 1H), 1.43 (s, 12H). ^{11}B NMR (128MHz, CD_2Cl_2) δ = 31.9 ($\nu_{½}$ = 216 Hz). $^{13}C\{^{1}H\}$ NMR (101MHz, CD_2Cl_2) δ = 135.7, 134.3, 134.3, 133.9, 130.9, 130.8, 126.0, 125.6, 120.9, 84.9, 84.7, 84.6, 25.7. HRMS (APCI) Calculated for $C_{18}H_{20}BO_2^+$ ($[M+H]^+$) 279.1551. Found 279.1553.

Synthesis of 4,4,5,5-tetramethyl-2-(8-(pent-1-yn-1-yl)naphthalene-1-yl)-1,3,2-dioxaborolane (**10b**)

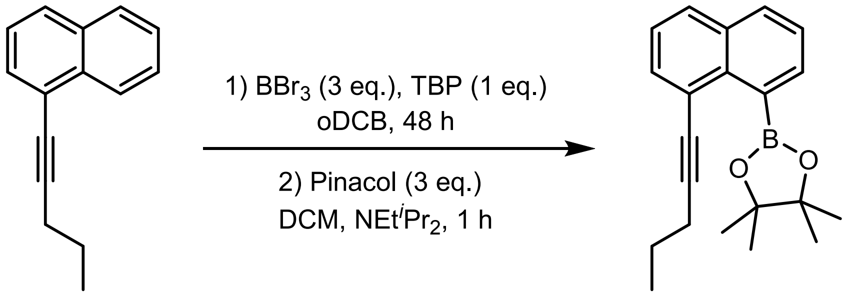

To an ampoule containing a solution of 1-(pent-1-yn-1-yl)naphthalene (0.150 g, 0.772 mmol, 1 eq.) and 2,4,6-tri-*tert*-butylpyridine (0.164 g, 0.663 mmol, 0.9 eq.) in *ortho*-dichlorobenzene (3 mL) was added dropwise a solution of boron tribromide (2.1 mL, 1.0 M in hexanes 2.1 mmol, 3 eq.). The mixture instantly develops a deep purple colour and was stirred for 2 days at ambient temperature during which time a green colour developed. All volatiles were removed *in vacuo* then the residue was taken up in dichloromethane (7 mL). To this was added dropwise a solution of pinacol (0.298 g, 2.52 mmol, 3 eq.) and Hünig's base (3 mL) in dichloromethane (3 mL). A bright orange suspension formed which was stirred for 1 hour. All volatiles were removed *in vacuo* and the product was extracted in pentane and filtered to give an orange solution. The product was purified by flash column chromatography using a DCM:petrol eluent (3:7) to yield analytically pure product **10b** at Rf = 0.28 as a yellow oil (98.8 mg, 0.308 mmol, 40%).

^{1}H NMR (400MHz, CD_2Cl_2) δ = 7.87 (d, 8.2 Hz, 1H), 7.80 (d, 8.3 Hz, 1H), 7.73 (m, 2H), 7.51 – 7.40 (m, 2H), 2.05 (td, 7.2, 2.5 Hz, 2H), 1.74 (qd, 7.3, 2.4 Hz, 2H), 1.43 (d, 2.3 Hz, 12H), 1.11 (td, 7.2, 2.9 Hz, 3H). ^{11}B NMR (128MHz, CD_2Cl_2) δ = 32.0 ($\nu_{½}$ = 369 Hz). ^{13}C{^{1}H} NMR (101MHz, CD_2Cl_2) δ = 135.4, 134.0, 133.9, 133.8, 130.8, 129.2, 125.8, 125.7, 123.4, 97.6, 84.3, 82.3, 25.7, 23.3, 23.0, 14.1. HRMS (APCI) Calculated for $C_{21}H_{26}BO_2^+$ ($[M+H]^+$) 321.2016. Found 321.2020.

Synthesis of 2-mesityl-3-(p-tolyl)-1H-naphtho[1,8-bc]borinin-1-ol (**11**)

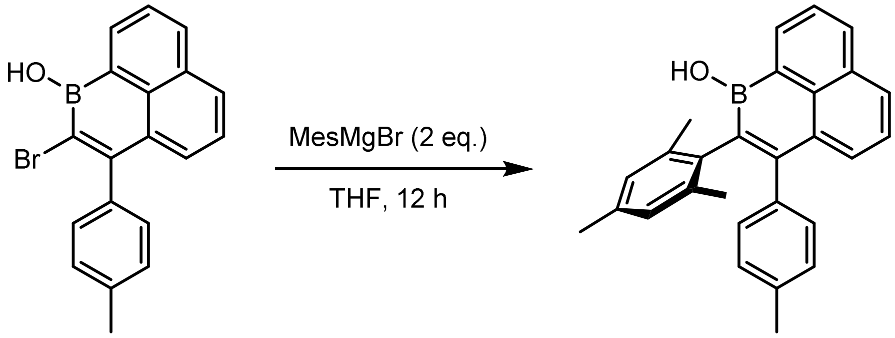

In an ampoule under argon flow **6c** (116.8 mg, 3.35 x 10^{-4} mol, 1 eq.) was dissolved in dry THF (5 mL). To the solution was added dropwise a solution of MesMgBr in THF (1.0 M, 0.8 mL, 8.0 x 10^{-4} mol). The mixture was stirred at ambient temperature overnight then all volatiles were removed *in vacuo*. The product was extracted in pentane to give a bright yellow solution and purified by flash column chromatography using DCM:petrol eluent (3:7 then 1:1) to yield analytically pure product at Rf = 0.31 as yellow crystals (84 mg, 2.2 x 10^{-4} mol, 65%). Compound **12** was obtained as a minor product (<10%) at Rf = 0.71.

Elemental Analysis Expected C 86.61% H 6.49%, Found C 86.33% H 6.49%. ^{1}H NMR (400MHz, CD_2Cl_2) δ = 7.94 (d, 7.8 Hz, 1H), 7.77 (d, 8.2 Hz, 1H), 7.67 – 7.59 (m, 2H), 7.48 (d, 7.7 Hz, 2H), 7.41 (dd, 8.2,

6.9 Hz, 1H), 7.27 (d, 7.6 Hz, 2H), 7.19 (d, 7.0 Hz, 1H), 6.80 (s, 2H), 6.04 (s, 1H), 2.43 (s, 3H), 2.31 (s, 3H), 2.25 (s, 6H). ^{11}B NMR (128MHz, CD_2Cl_2) δ = 47.3 ($\nu_{½}$ = 654 Hz). ^{13}C{^{1}H} NMR (101MHz, CD_2Cl_2) δ = 156.2, 143.6, 141.4, 139.5, 138.9, 138.4, 133.6, 130.0, 129.9, 129.7, 129.0, 128.1, 127.7, 126.9, 126.2, 125.7, 22.4, 21.6, 21.6.

Synthesis of 2-bromo-1-mesityl-3-(p-tolyl)-1H-naphtho[1,8-bc]borinine (**12**)

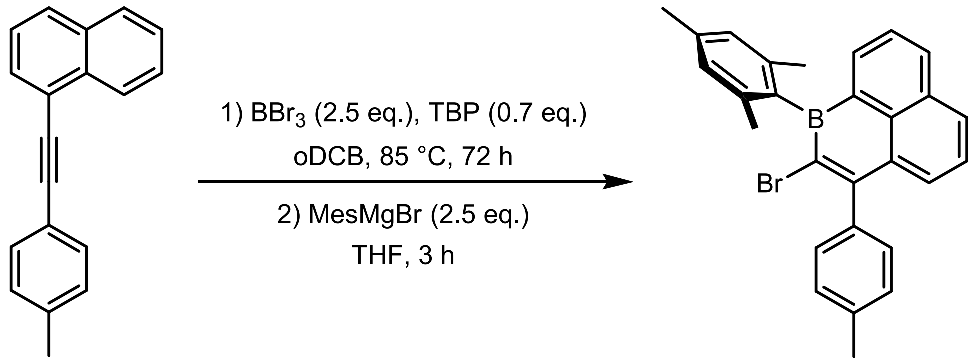

To an ampoule under argon flow was added **4c** (0.248 g, 1.02 mmol, 1 eq.), 2,4,6-tri-*tert*-butylpyridine (0.175 g, 0.707 mmol, 0.7 eq.) and dry *ortho*-dichlorobenzene (4 mL). To this was added BBr_3 (2.50 mL, 1.0 M in hexanes, 2.50 mmol, 2.5 eq.) and a darkening of the solution ensued. The mixture was heated to 85 °C for 72 hours. Upon cooling, all volatiles were removed *in vacuo*. The crude boraphenalene **5c** was extracted in dry pentane (300 mL) and filtered to give an orange solution. All volatiles were removed *in vacuo* and the residue (0.388 g) was taken up in dry THF (5 mL). To the solution was added dropwise 2-mesitylmagnesium bromide (2.5 mL, 1.0 M in THF, 2.5 mmol, 2.5 eq. assuming all the residue was **5c**). The mixture was stirred under ambient conditions for three hours then all volatiles were removed *in vacuo*. The product was extracted in dichloromethane to give a yellow solution which was purified by flash column chromatography using a DCM:petrol eluent (15:85) to yield analytically pure product **12** at Rf = 0.35 as a yellow oil (0.252 g, 0.56 mmol, 55% with respect to **4c**).

^{1}H NMR (400MHz, CD_2Cl_2) δ = 8.34 (dd, 8.0, 1.4 Hz, 1H), 8.18 (dd, 6.9, 1.3 Hz, 1H), 8.12 (dd, 8.0, 1.2 Hz, 1H), 7.72 (dd, 8.0, 7.0 Hz, 1H), 7.62 (dd, 7.5, 1.2 Hz, 1H), 7.54 (m, 1H), 7.37 (d, 7.8 Hz, 2H), 7.27 (d, 7.7 Hz, 2H), 6.92 (s, 2H), 2.49 (s, 3H), 2.37 (s, 3H), 2.09 (s, 6H). ^{11}B NMR (128MHz, CD_2Cl_2) δ = 58.0 ($\nu_{½}$ = 828 Hz). ^{13}C{^{1}H} NMR (101MHz, CD_2Cl_2) δ = 159.9, 144.5, 140.8, 138.5, 138.2, 137.6, 137.1, 137.0, 136.2, 135.0, 134.8, 134.1, 132.8, 131.9, 129.6, 129.1, 128.7, 126.9, 126.8, 126.0, 22.8, 21.2, 21.0. HRMS (APCI) Calculated for $C_{28}H_{24}BBr^+$ (M^+) 450.1149. Found 450.1148.

Synthesis of 1,2-dimesityl-3-(p-tolyl)-1H-naphtho[1,8-*bc*]borinine (**13**) and 1-mesityl-3-(p-tolyl)-1H-naphtho[1,8-bc]borinine (**14**)

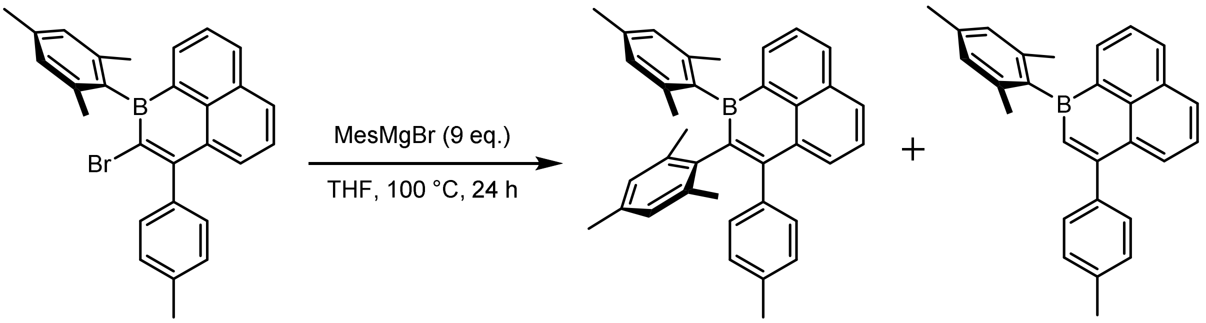

To an ampoule under argon flow was added **12** (78.1 mg, 0.173 mmol, 1 eq.) and 2-mesitylmagnesium bromide (1.3 mL, 1.0 M in THF, 1.3 mmol, 9 eq.).* The mixture was heated to 100 °C for 24 hours then all volatiles were removed *in vacuo*. The products were extracted in wet dichloromethane and filtered to give an orange solution containing **13** and **14** (*ca*. 1:3 ratio). Both products were purified by flash column chromatography using a DCM:petrol eluent (1:9 then 1:1) to yield **14** at Rf = 0.30 (27.4 mg, 7.4 x 10^{-5} mol, 42%) and **13** at Rf = 0.20 (14.4 mg, 2.9 x 10^{-5} mol, 17%).

*The reaction only proceeds significantly using isolated **12** after purification by column chromatography.

13: ^{1}H NMR (400MHz, CD_2Cl_2) δ = 8.29 (dd, 8.0, 1.4 Hz, 1H), 8.07 (dd, 7.1, 2.3 Hz, 1H), 7.95 (dd, 6.9, 1.4 Hz, 1H), 7.68 (dd, 8.0, 6.9 Hz, 1H), 7.58 – 7.50 (m, 2H), 7.13 (d, 8.0 Hz, 2H), 7.05 (d, 7.8 Hz, 2H), 6.69 (s, 2H), 6.55 (s, 2H), 2.32 (s, 3H), 2.25 (s, 3H), 2.10 (s, 3H), 2.02 (s, 6H), 1.85 (s, 6H). ^{11}B NMR (128MHz, CD_2Cl_2) δ = 59.9 ($\nu_{½}$ = 776 Hz). ^{13}C{^{1}H} NMR (101MHz, CD_2Cl_2) δ = 156.8, 142.2, 140.2, 138.9, 137.9, 136.5, 136.3, 135.7, 134.7, 134.3, 134.1, 133.8, 131.7, 131.6, 129.8, 129.4, 127.5, 127.4, 126.7, 126.5, 125.6, 23.5, 21.3, 20.9, 20.8, 20.5. HRMS (APCI) Calculated for $C_{37}H_{36}B^{+}$ ($[M+H]^{+}$) 491.2905. Found 491.2905.

14: ^{1}H NMR (400MHz, CD_2Cl_2) δ =8.21 (dd, 8.0, 1.5 Hz, 1H), 8.06 (dd, 6.9, 1.5 Hz, 1H), 8.03 (dd, 8.2, 1.2 Hz, 1H), 7.87 (dd, 7.4, 1.2 Hz, 1H), 7.63 (dd, 8.1, 6.9 Hz, 1H), 7.53 (dd, 8.0, 7.4 Hz, 1H), 7.34 (d, 8.0 Hz, 2H), 7.22 (d, 7.8 Hz, 2H), 6.97 (s, 1H), 6.81 (s, 2H), 2.37 (s, 3H), 2.25 (s, 3H), 1.99 (s, 6H).). ^{11}B NMR (128MHz, CD_2Cl_2) δ = 57.6 ($\nu_{½}$ = 667 Hz). ^{13}C{^{1}H} NMR (101MHz, CD_2Cl_2) δ =162.0, 143.2, 140.9, 139.0, 138.1, 137.1, 136.5, 134.3, 133.4, 132.9, 132.7, 131.5, 130.1, 129.2, 127.4, 127.0, 126.1, 23.7, 21.5, 21.5.# HRMS (APCI) Calculated for $C_{28}H_{26}B^{+}$ ($[M+H]^{+}$) 373.2122. Found 373.2121.

#Full assignment of the ^{13}C{^{1}H} NMR are presented on page S36.

Synthesis of 3-bromo-1-mesityl-1H-naphtho[1,8-bc]borinine (**15**)

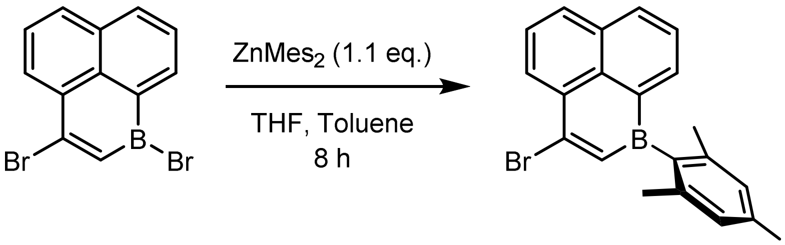

To an ampoule containing **9** (46 mg, 1.43 x 10^{-4} mol, 1 eq.) and $ZnMes_2$ (45 mg, 1.48 x 10^{-4} mol, 1.1 eq.) was added toluene (0.7 mL) and 50 µL THF to give a dark green suspension.* The mixture

becomes red/brown within 15 minutes and was stirred for 16 hours. All volatiles were removed *in vacuo* to give a brown residue. The product **15** was extracted in hexane and filtered through glass wool then dried *in vacuo* to give an oily solid that was pure by NMR spectroscopy (45.6 mg, 1.3×10^{-4} mol, 88%).

***15** can also be prepared by reaction of **9** with MesMgBr (1.0 M in THF). However this reaction generates multiple side products which limit the yield to 22%. THF is required for the reaction with $ZnMes_2$ to take place, possibly to disrupt the extended π-stacking of **9** by coordination of THF to boron.

^{1}H NMR (400MHz, $CDCl_3$) δ = 8.64 (dd, 7.4, 1.0 Hz, 1H), 8.25 (dd, 8.1, 1.1 Hz, 1H), 8.19 – 8.11 (m, 2H), 7.74 (dd, 8.1, 7.4 Hz, 1H), 7.70 (s, 1H), 7.67 (ddd, 8.0, 7.0, 0.8 Hz, 1H), 6.92 (s, 2H), 2.37 (s, 3H), 2.08 (s, 6H). ^{11}B NMR (128MHz, $CDCl_3$) δ = 56.6 ($\nu_{½}$ = 922 Hz). ^{13}C{^{1}H} NMR (101MHz, $CDCl_3$) δ = 146.3, 143.9, 141.6, 138.5, 137.2, 136.2, 135.5, 133.6, 131.8, 131.6, 131.4, 129.4, 128.3, 127.0, 126.7, 125.9, 23.4, 21.2. HRMS (APCI) Calculated for $C_{21}H_{19}BBr^+$ ($[M+H]^+$) 361.0750. Found 361.0746.

NMR Spectra

Crude residue of **6b** and Compound **C**

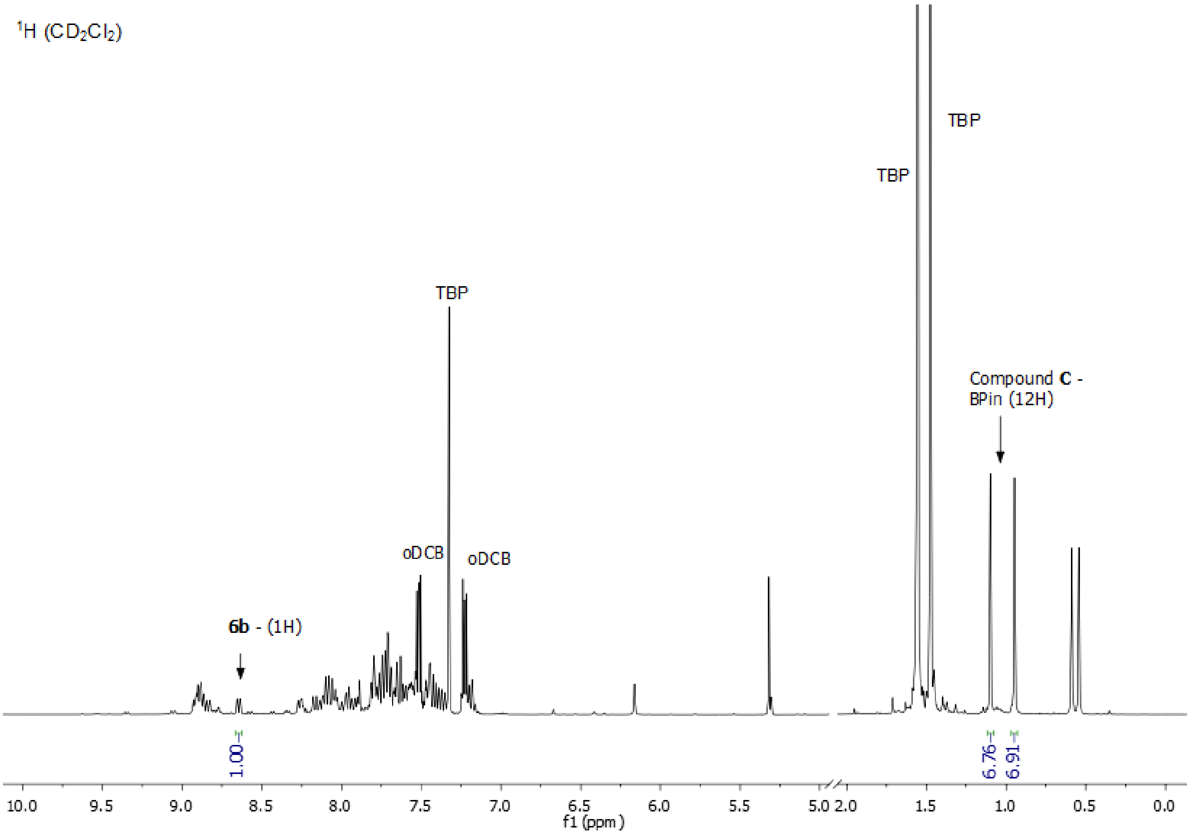

9-(naphthalene-1-yl)phenanthrene

The data for this compound is comparable to that previously reported.[4]

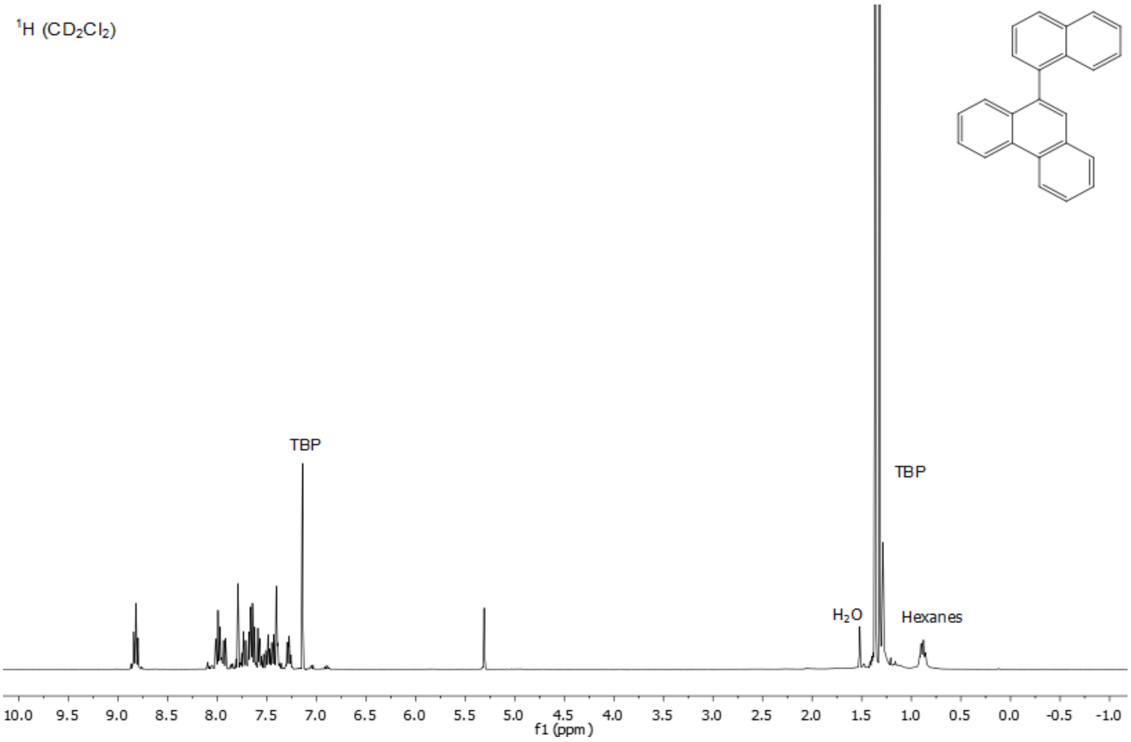

Comparative Crude Spectra of Compounds **6a** – **6c**

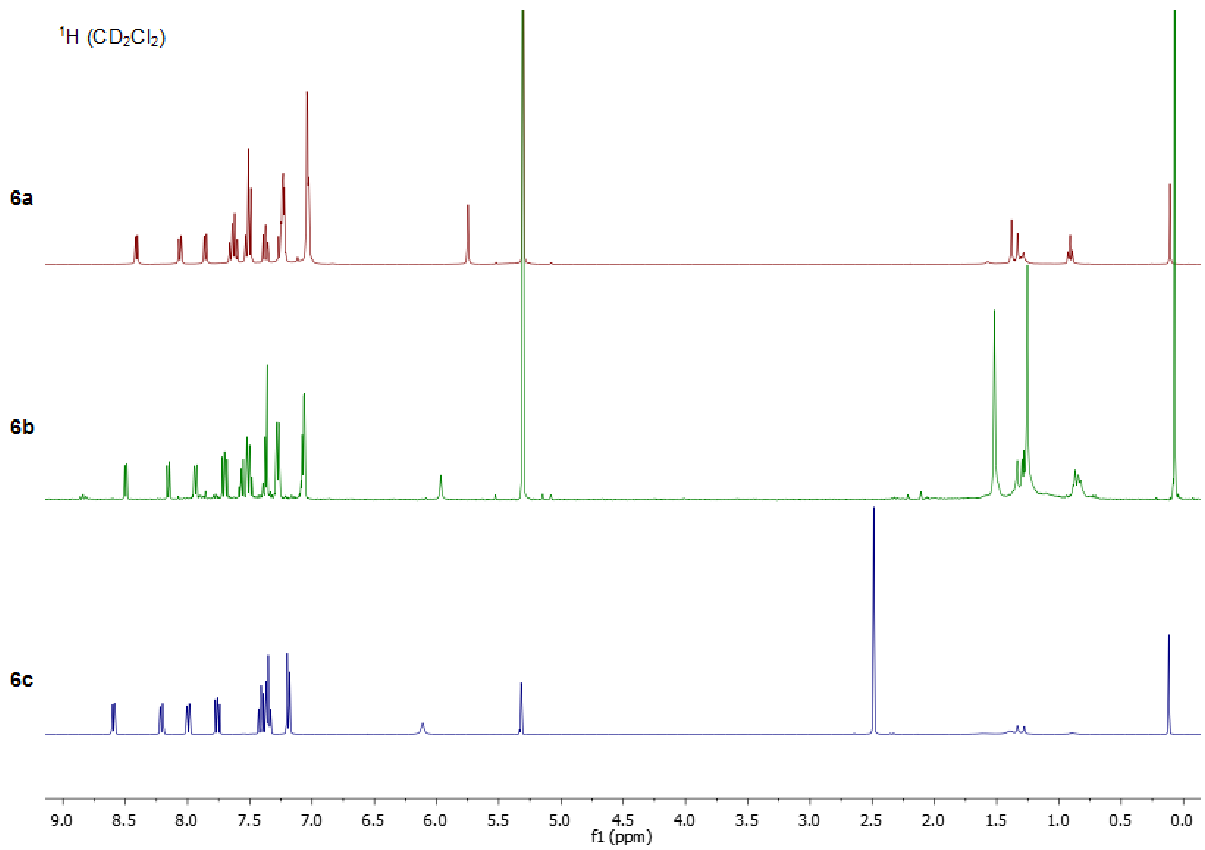

1-([1,1':3',1''-terphenyl]-2'-ylethynyl)naphthalene (**4a**)

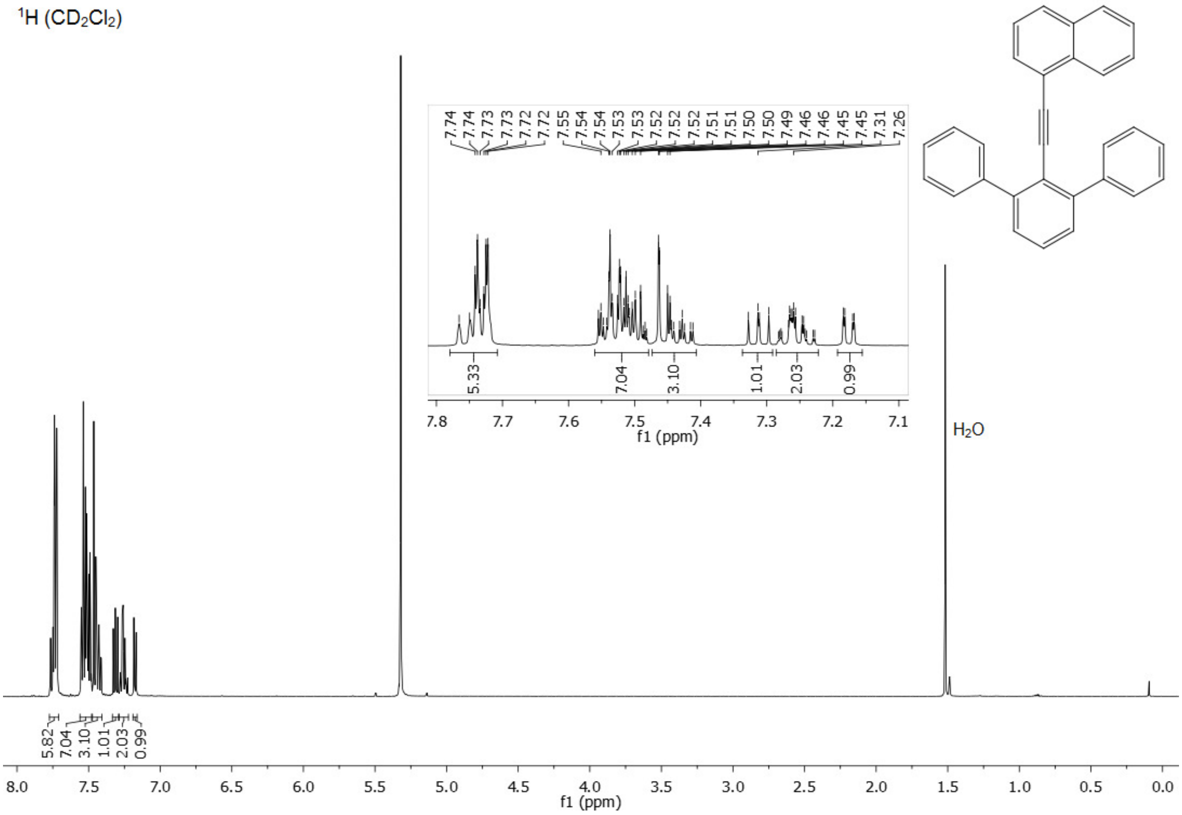

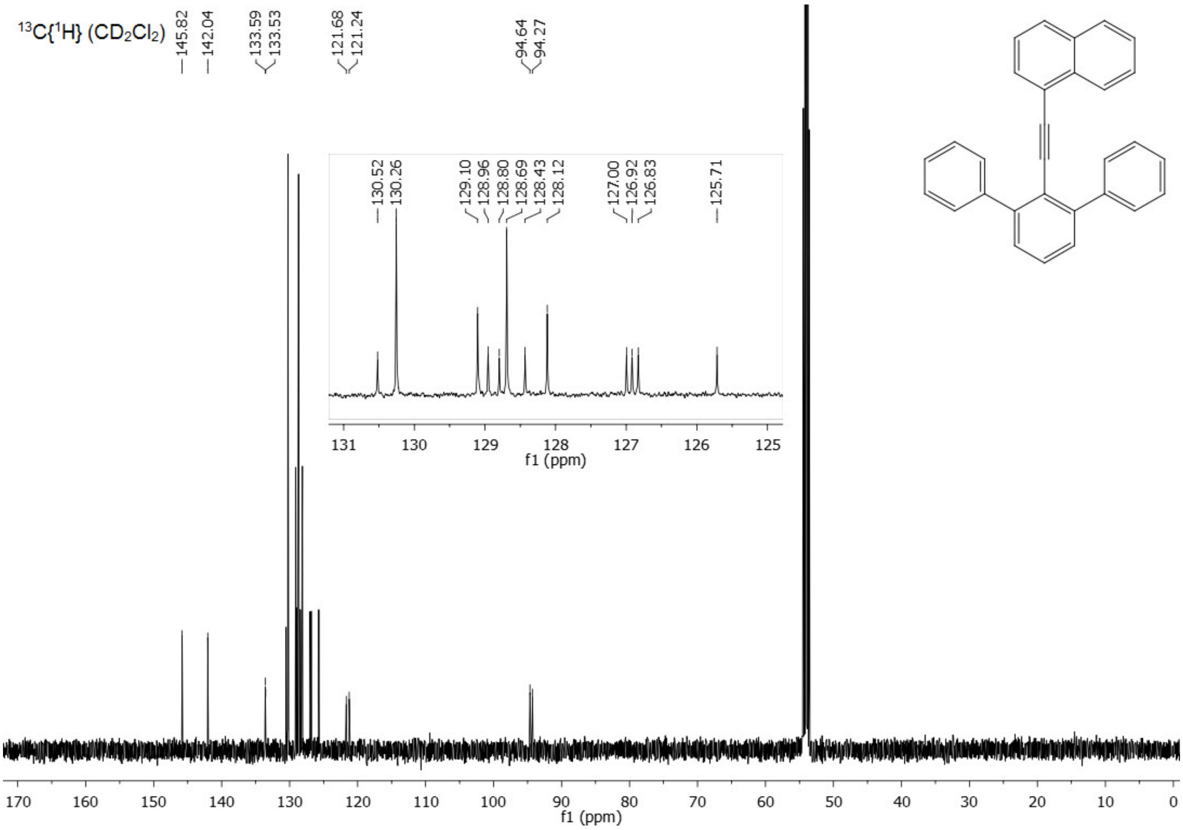

1-([1,1′-biphenyl]-2-ylethynyl)naphthalene (**4b**)

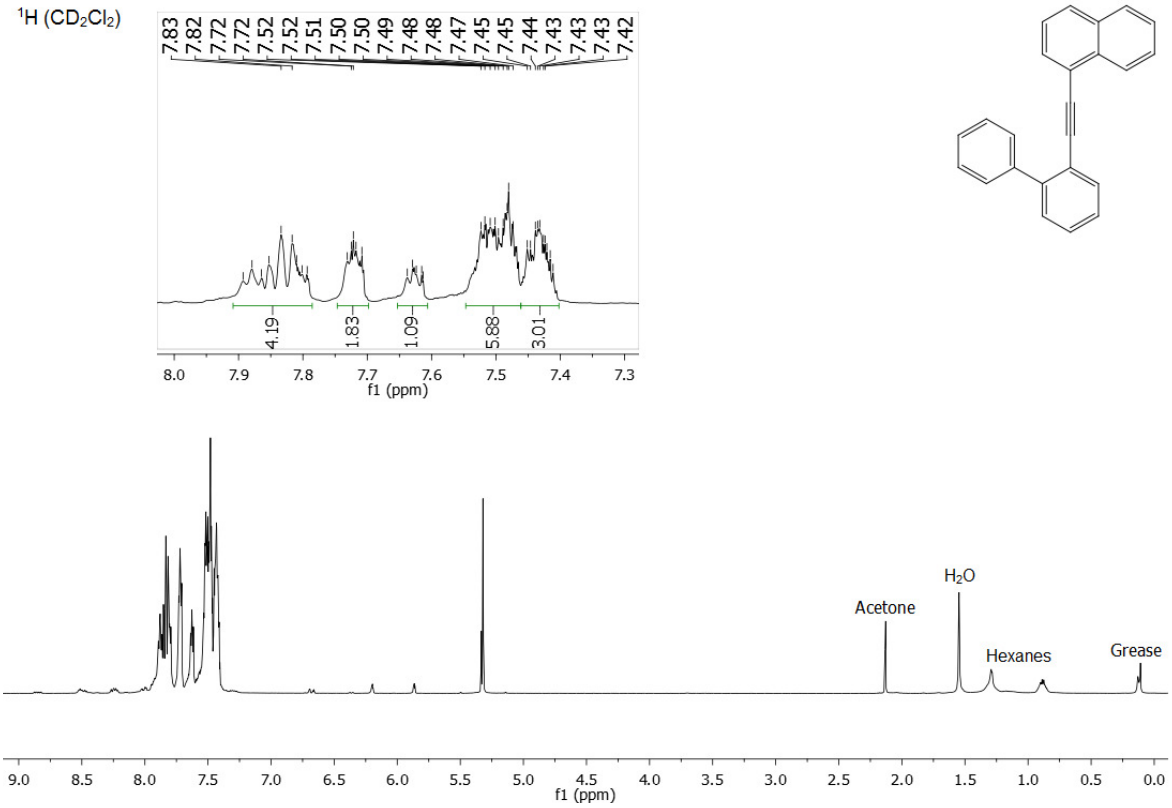

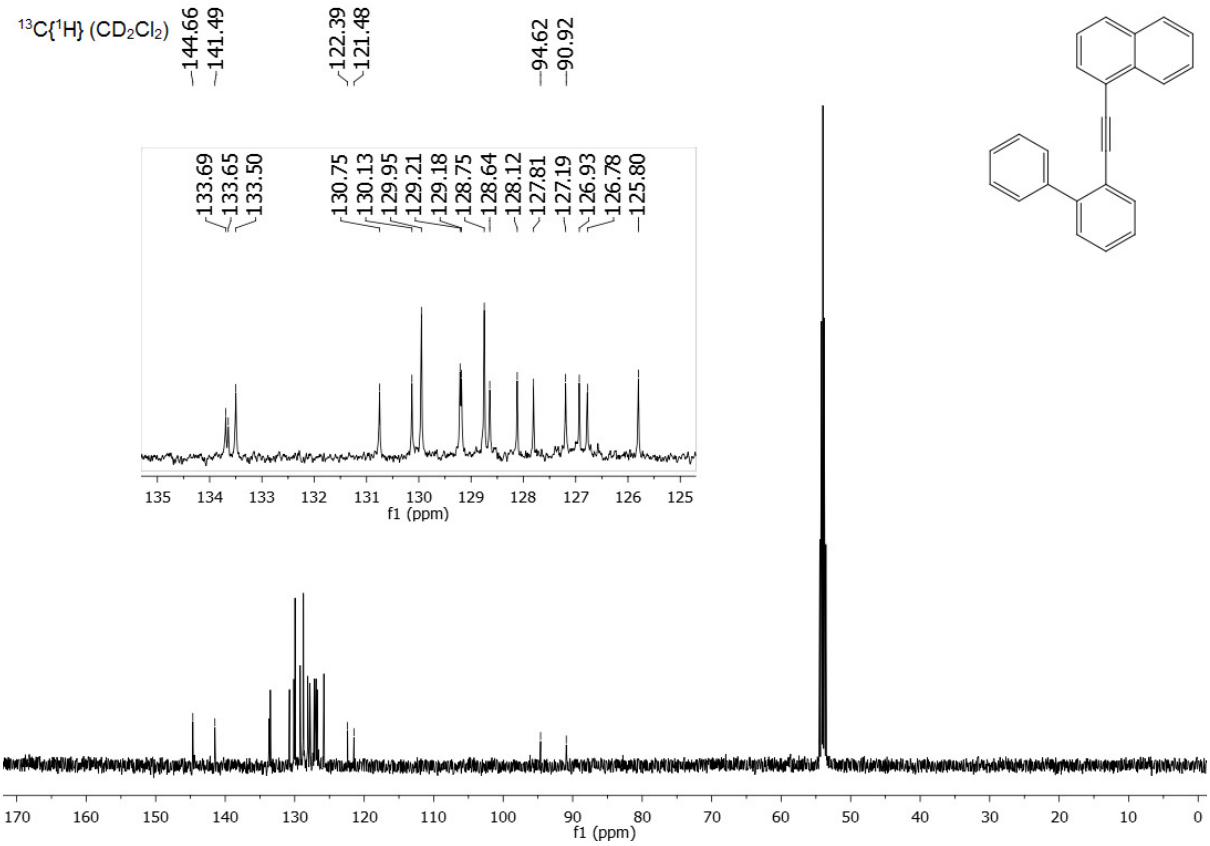

<u>(1-(p-tolylethynyl)naphthalene) (**4c**)</u>

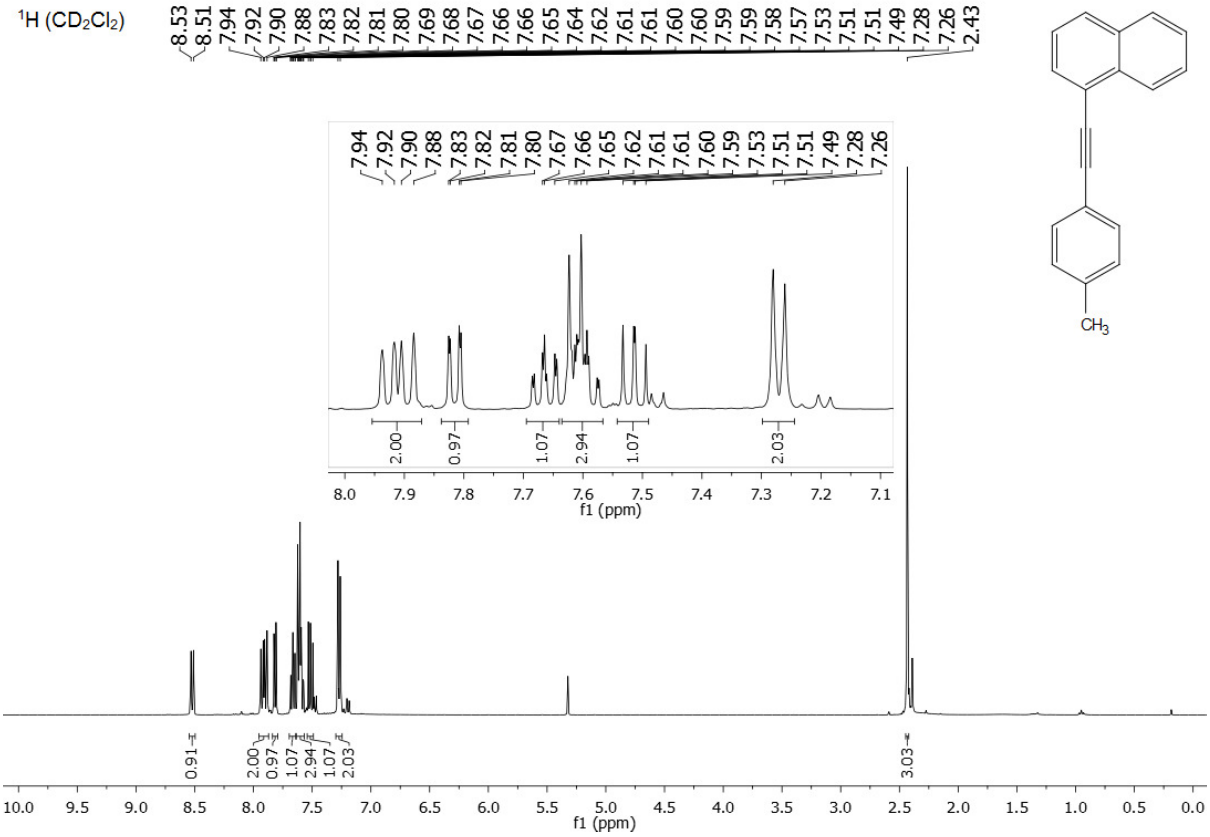

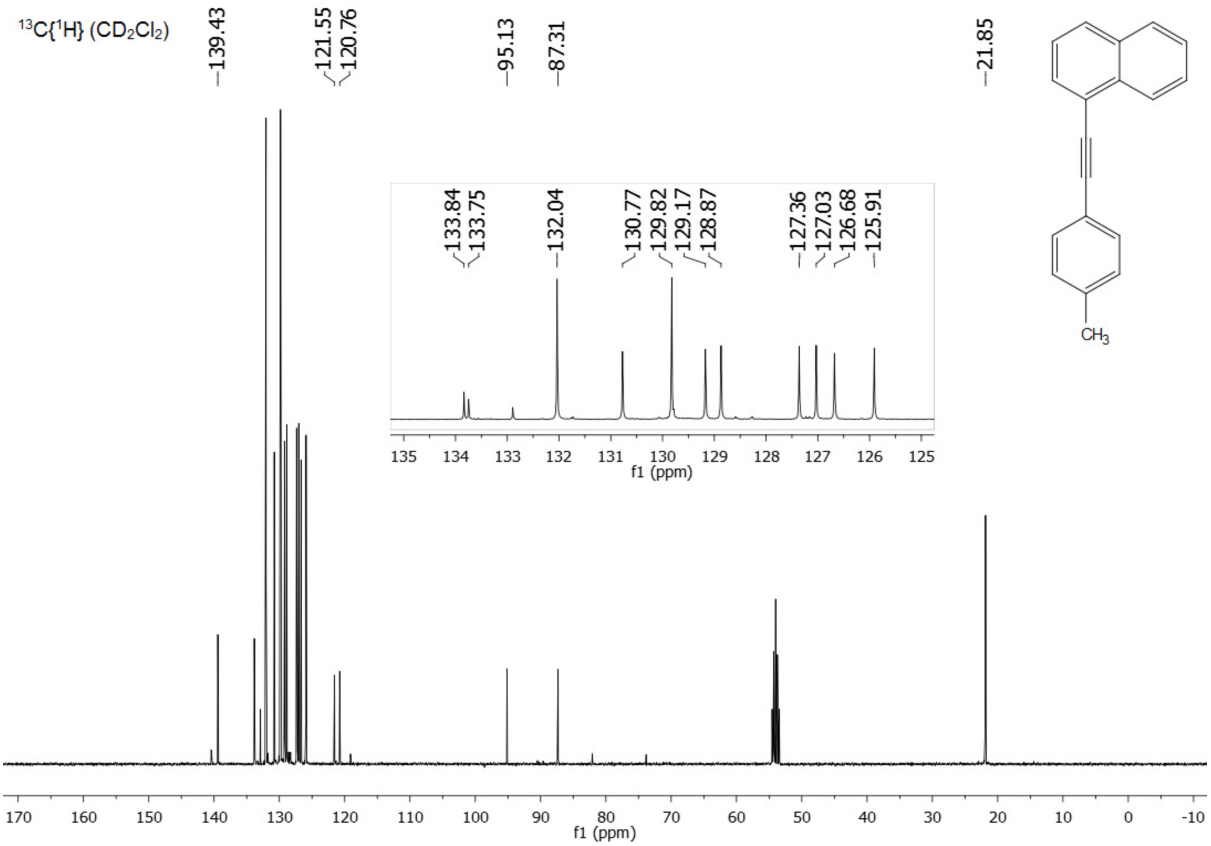

1-(pent-1-yn-1-yl)naphthalene (**7b**)

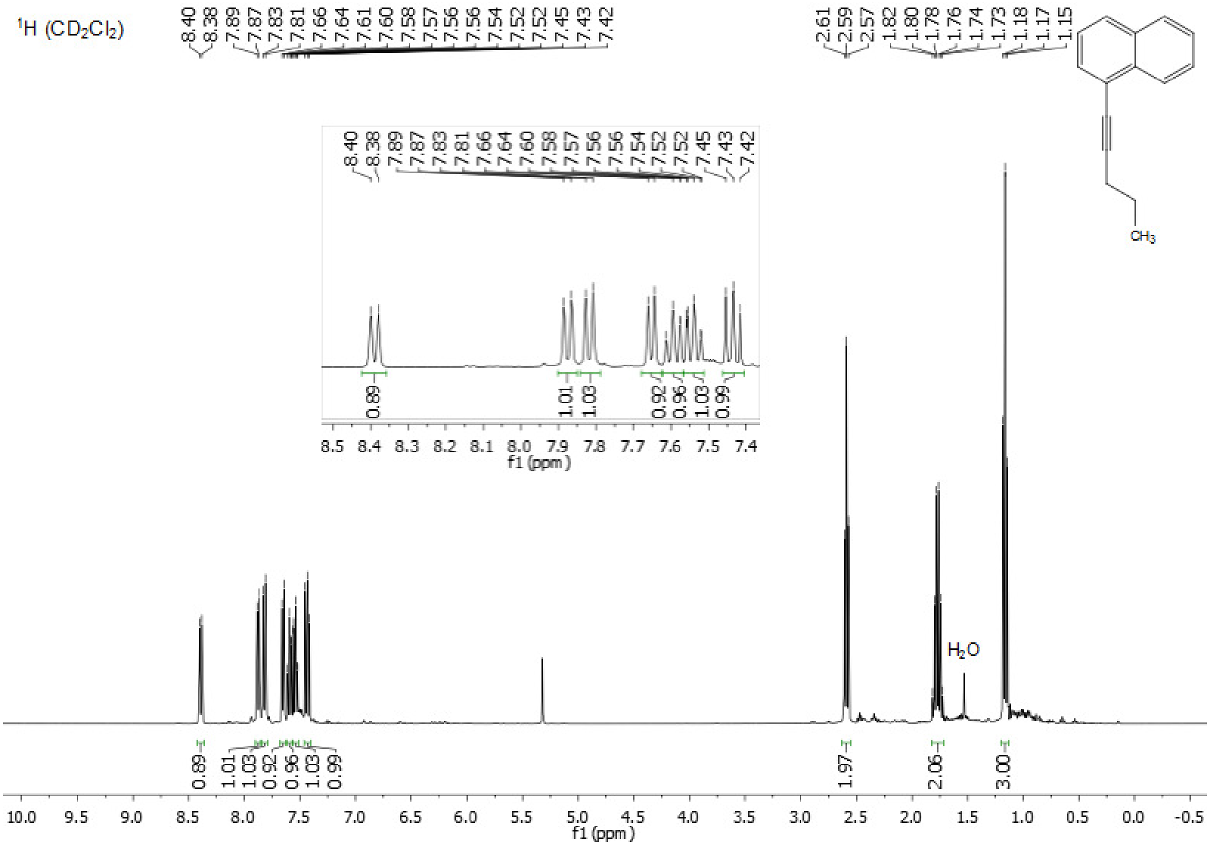

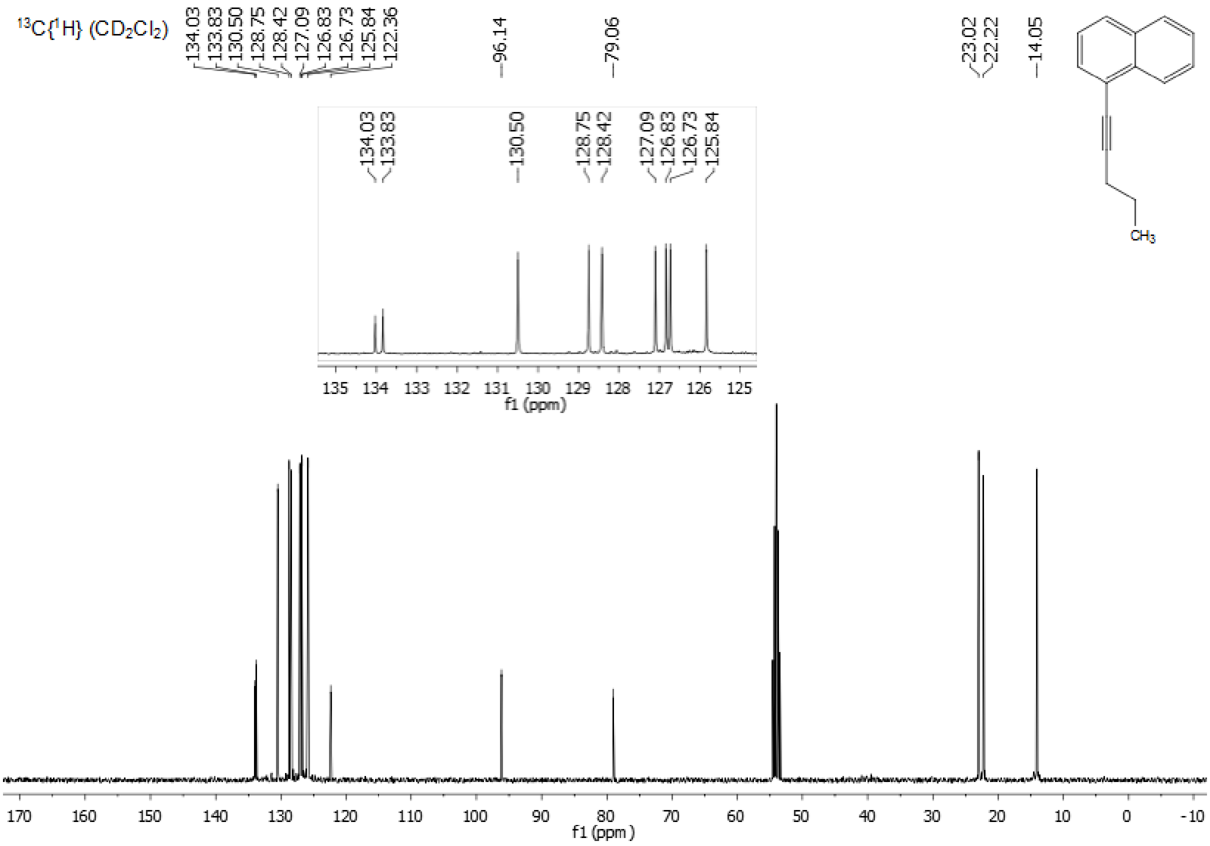

3-([1,1':3',1''-terphenyl]-2'-yl)-2-bromo-1H-naphtho[1,8-bc]borinin-1-ol (**6a**)

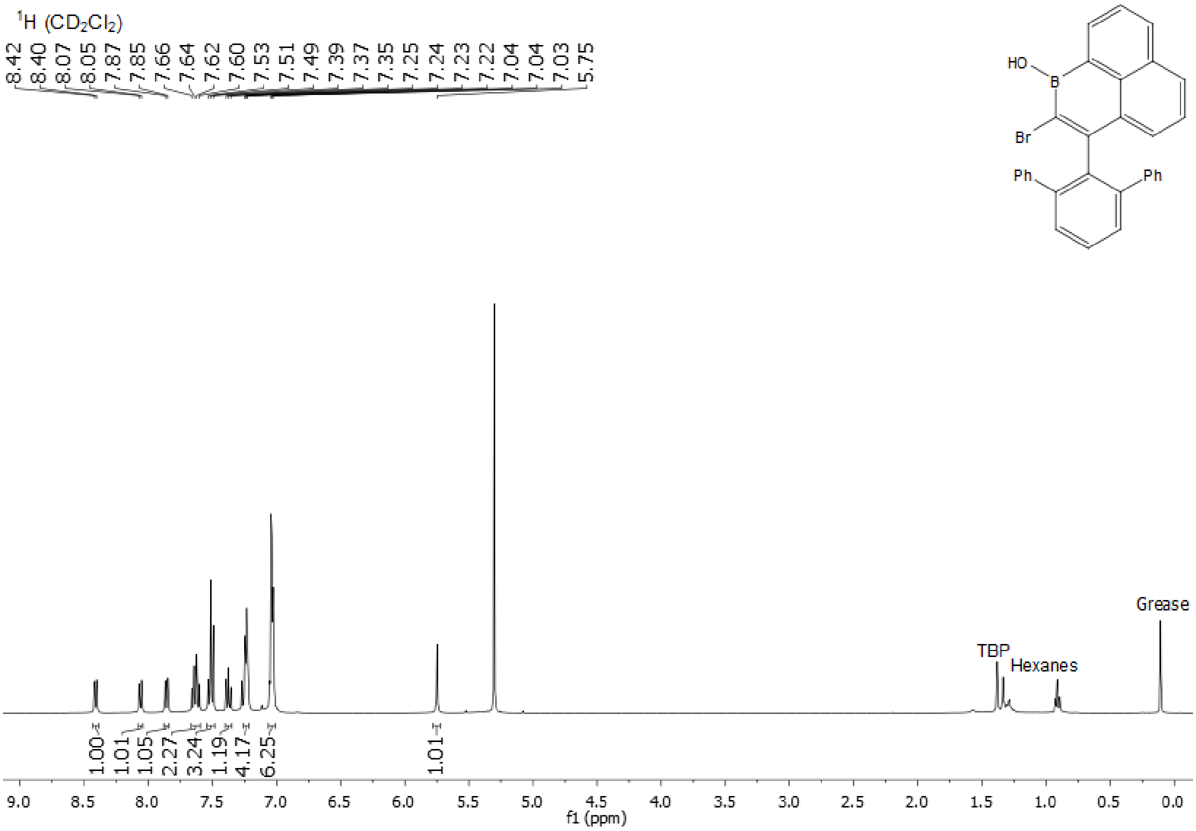

Note this is a crude spectrum prior to column purification. Post purification the compound has extremely low solubility in common NMR solvents (e.g. $CDCl_3$, CD_2Cl_2). We presume the presence of

pyridyl (TBP) helps solubility by hydrogen bonding to the O-H moiety. Below is a ^{1}H NMR spectrum of the purified sample (that microanalysis was performed on), this is of a saturated solution in $CDCl_3$ (containing a little TMS), demonstrating its poorer solubility post chromatography.

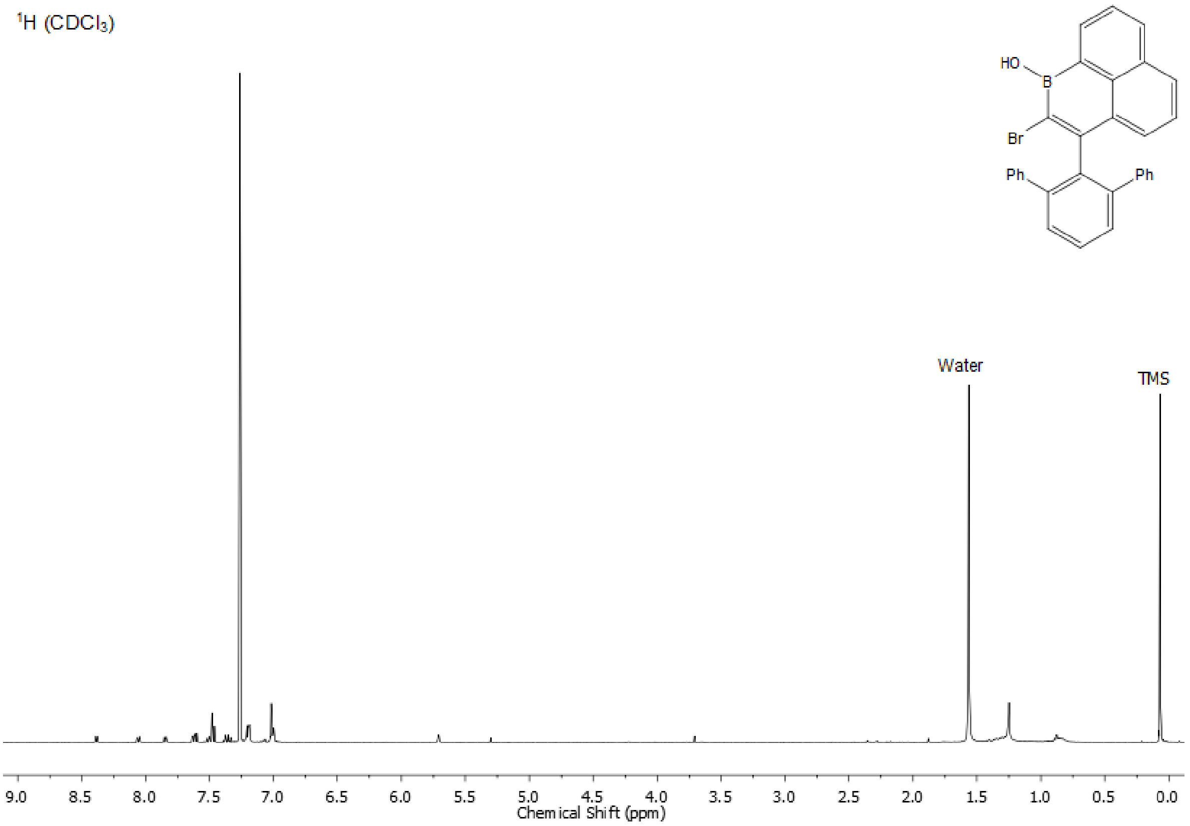

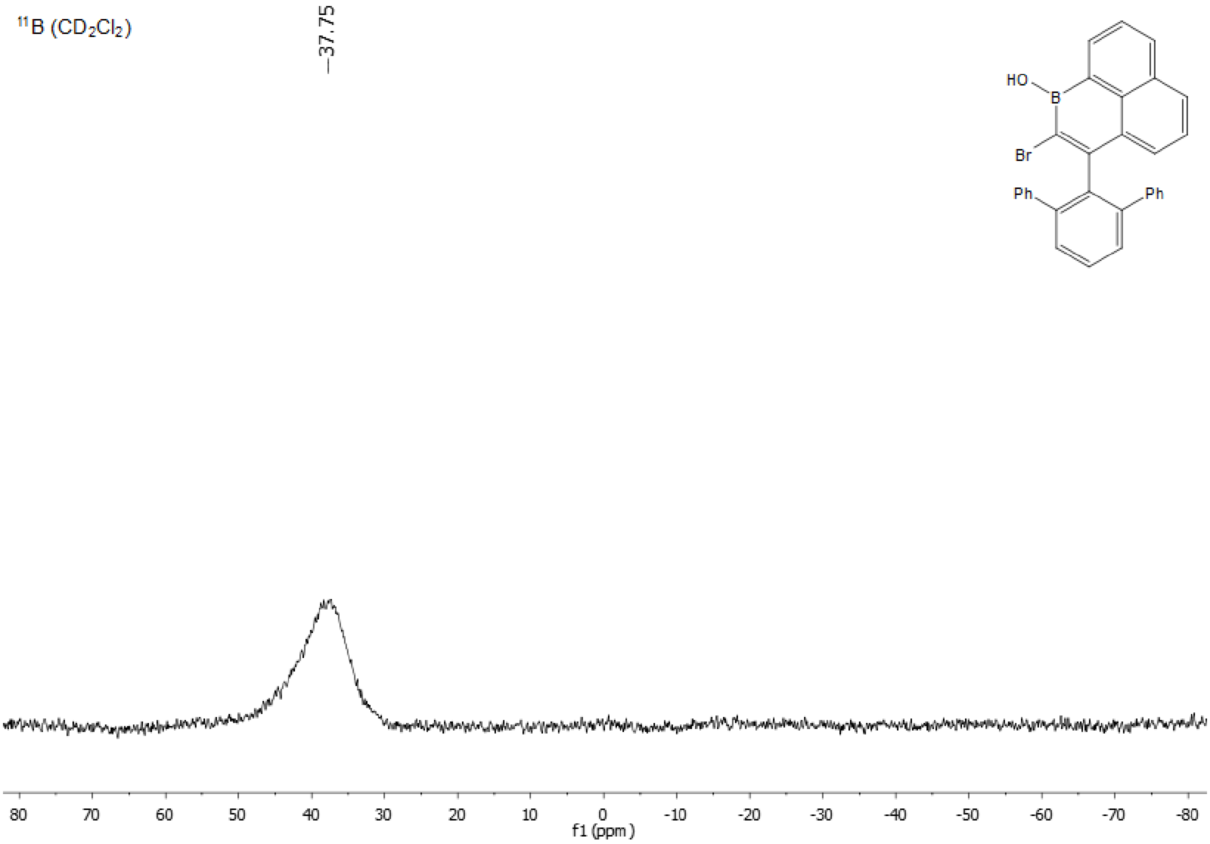

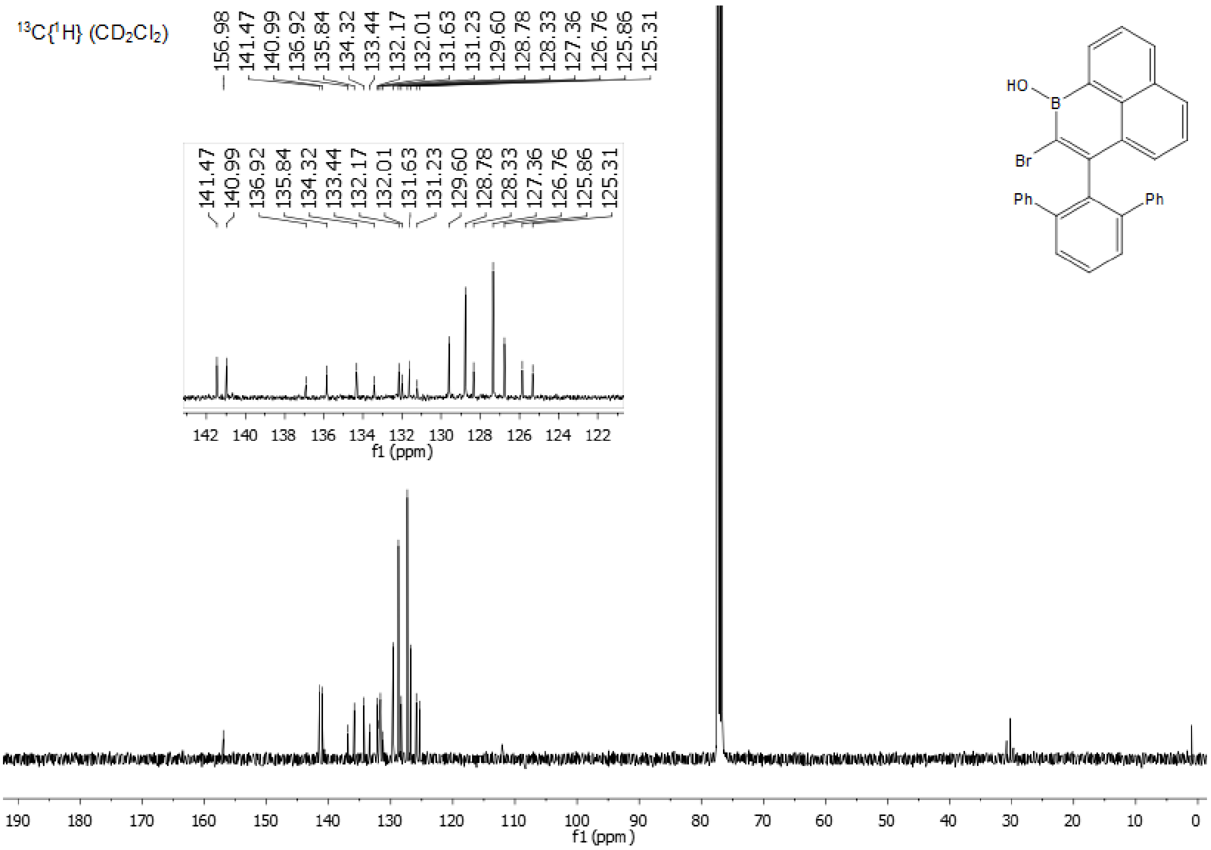

3-([1,1'-biphenyl]-2-yl)-2-bromo-1H-naphtho[1,8-bc]borinin-1-ol (**6b**)

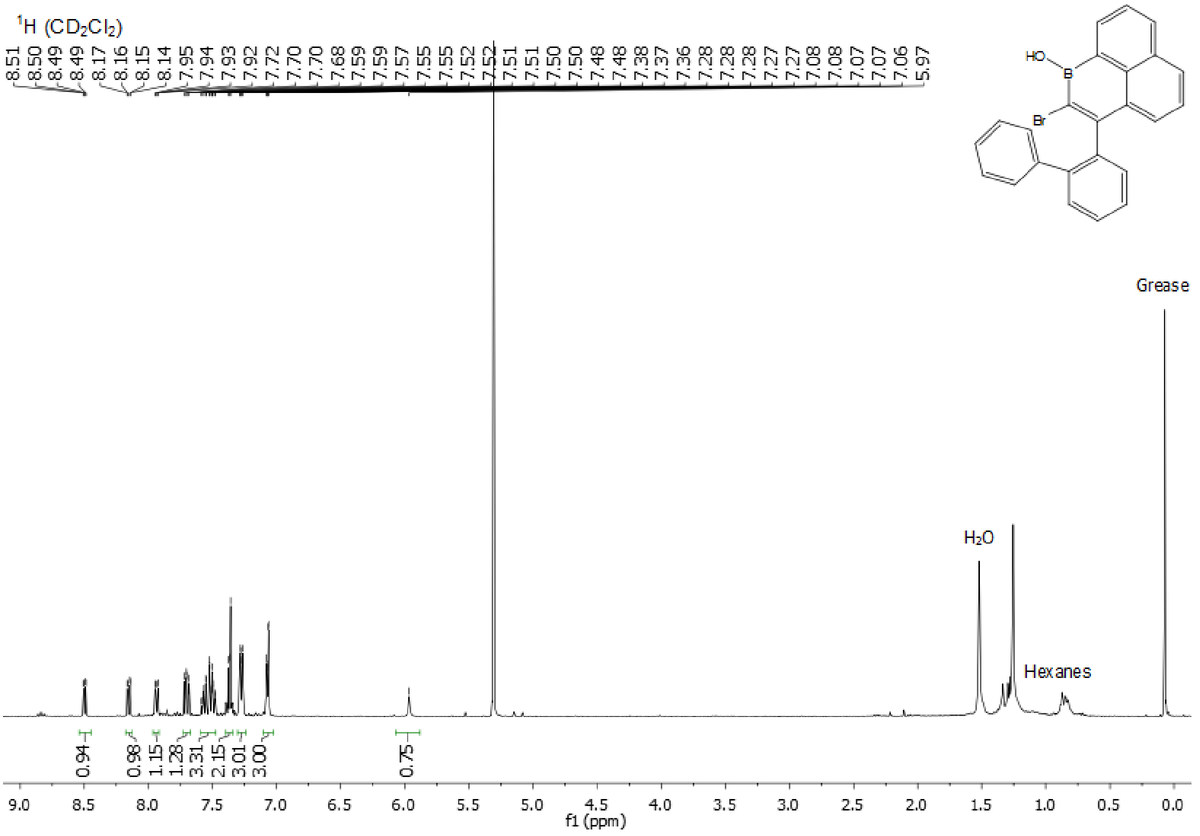

4,4,5,5-tetramethyl-2-(10-(naphthalen-1-yl)phenanthren-9-yl)-1,3,2-dioxaborolane (Compound **C**)

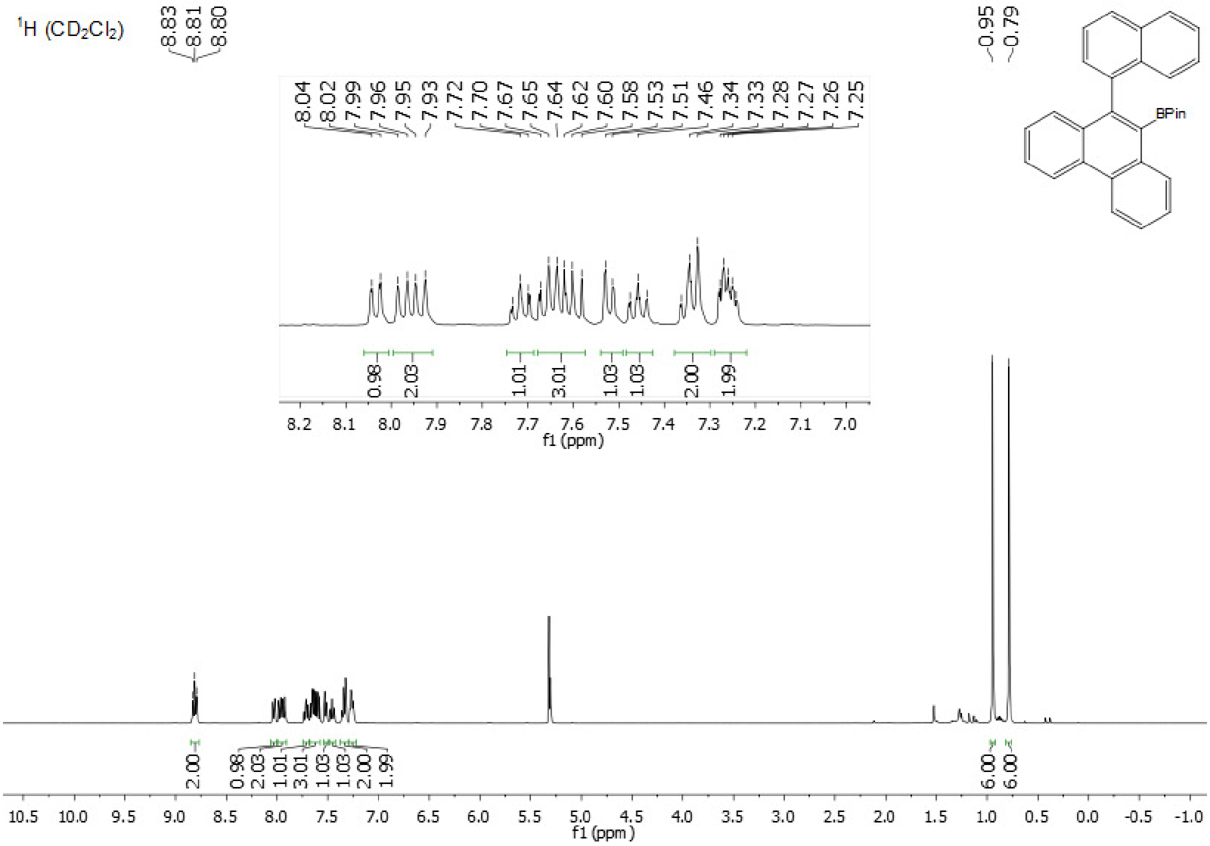

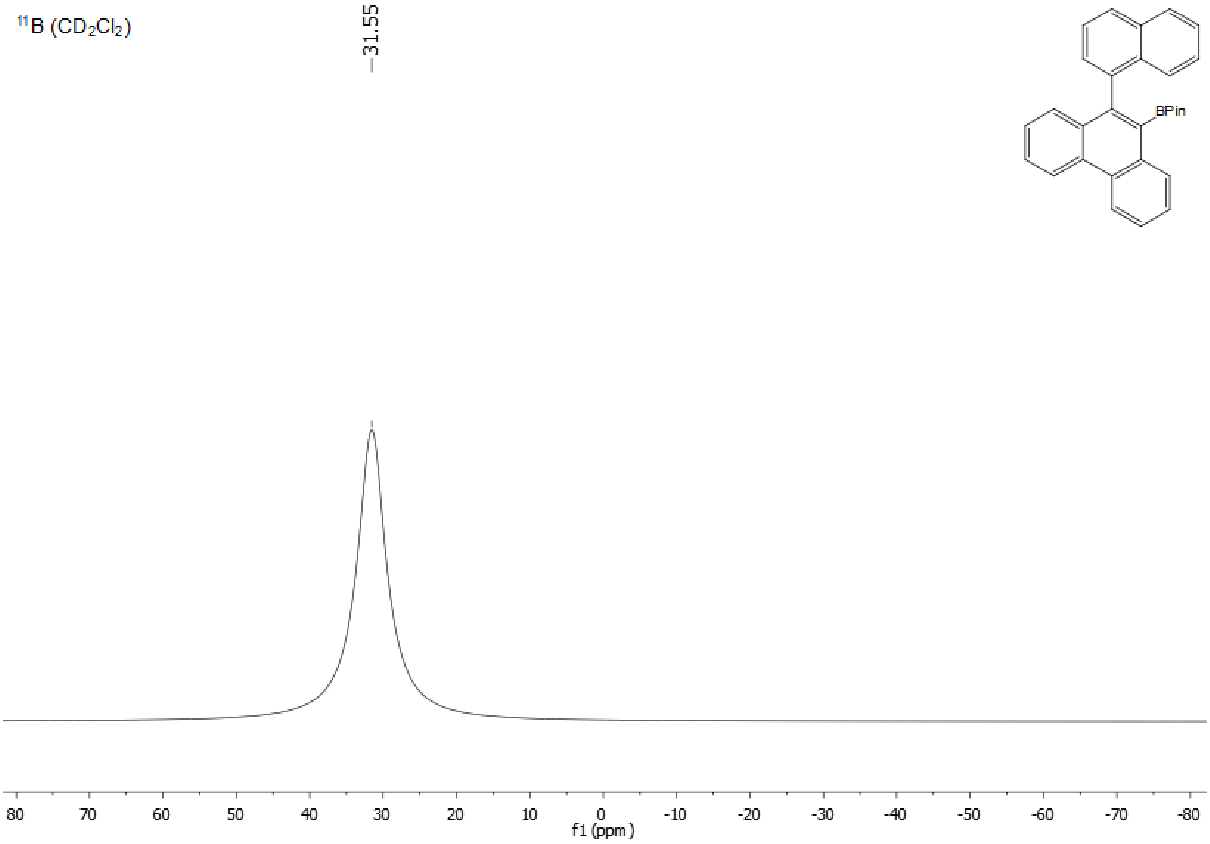

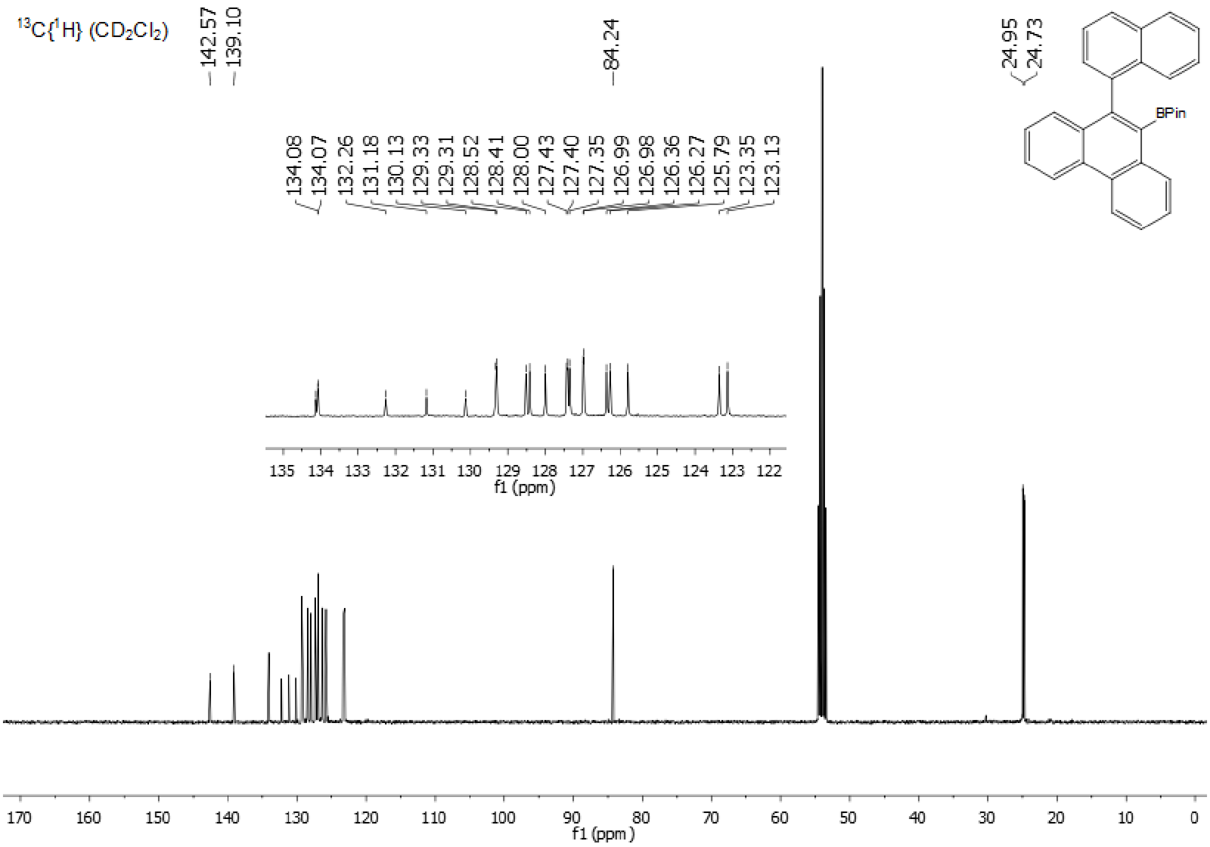

2-bromo-3-(p-tolyl)-1H-naphtho[1,8-bc]borinin-1-ol (**6c**)

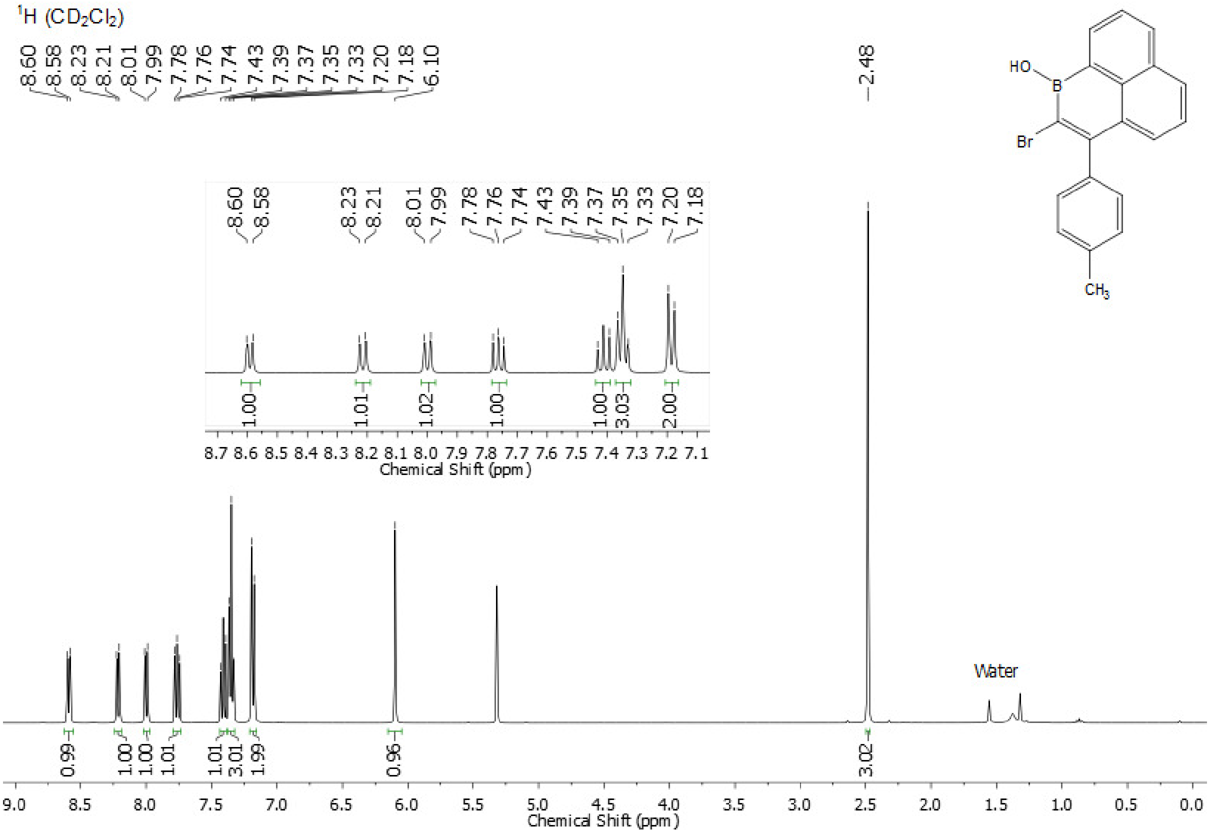

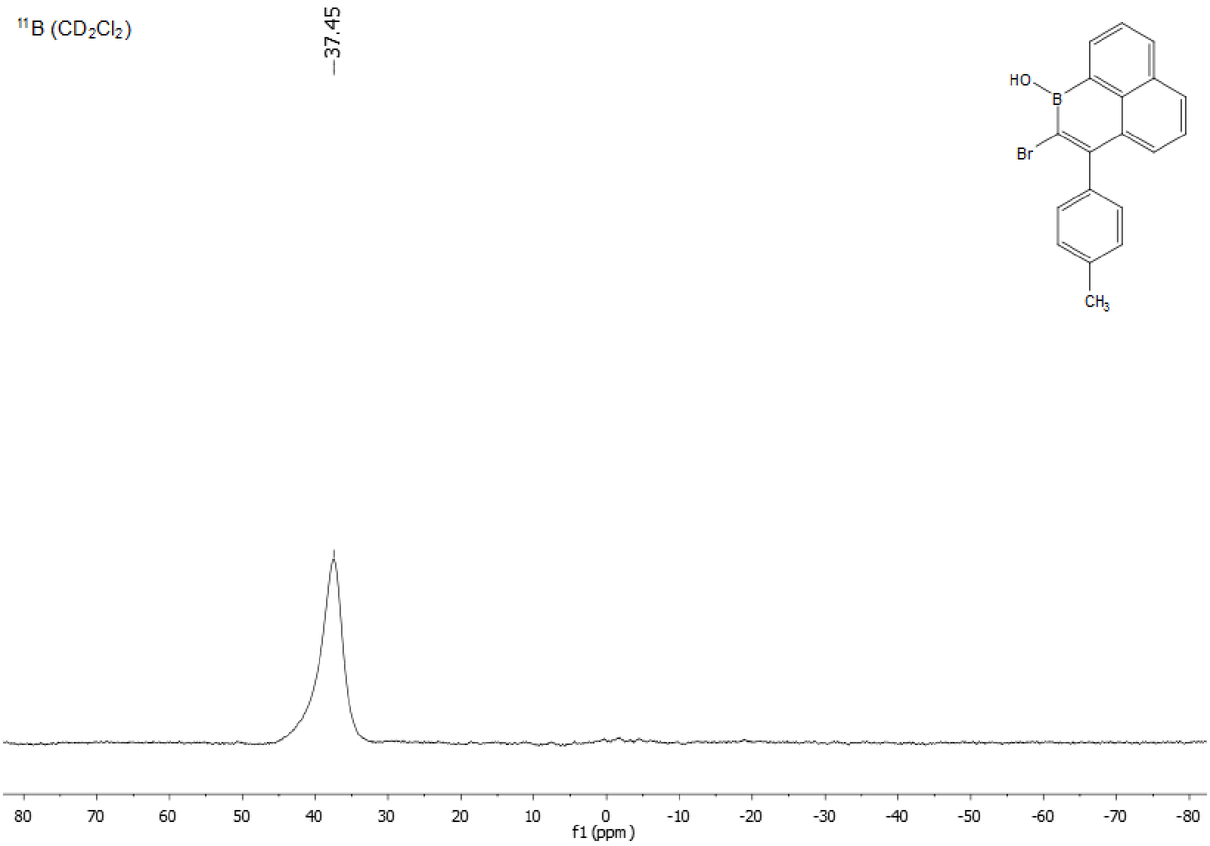

^{11}B (CD_2Cl_2)
37.45
HO
B
Br
CH_3
f1 (ppm)

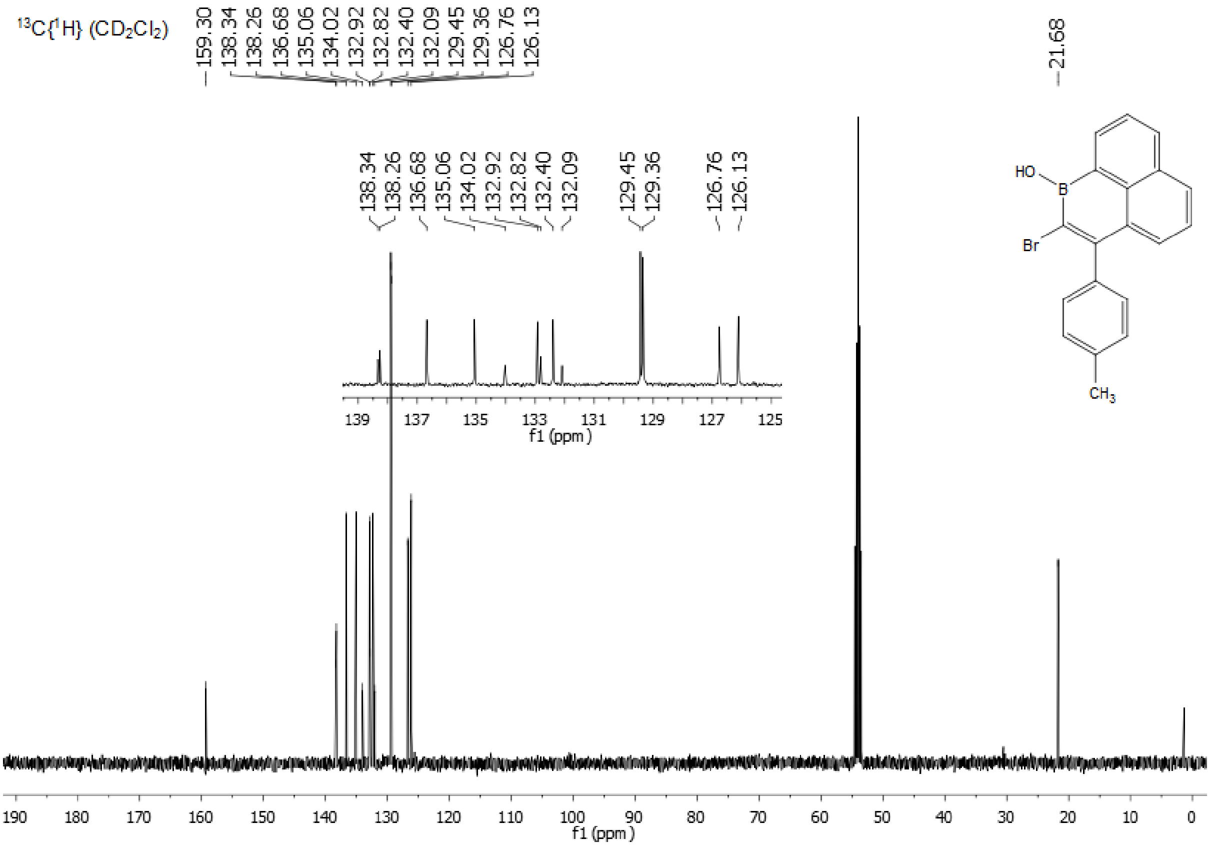

$^{13}C\{^1H\}$ (CD_2Cl_2)
159.30
138.34
138.26
136.68
135.06
134.02
132.92
132.82
132.40
132.09
129.45
129.36
126.76
126.13
21.68
HO
B
Br
CH_3
f1 (ppm)

(E)-2-(2-bromo-2-(naphthalen-1-yl)vinyl)-4,4,5,5-tetramethyl-1,3,2-dioxaborolane (**8-BPin**)

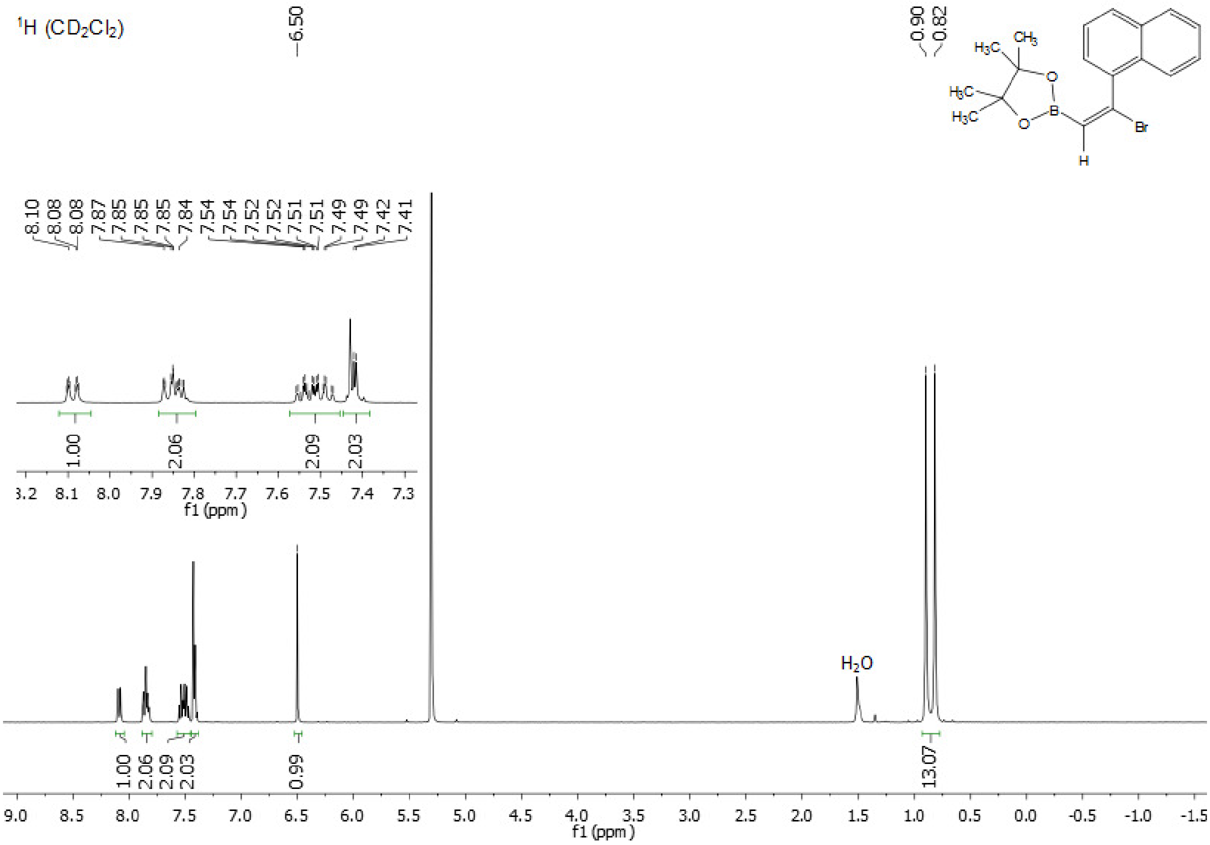

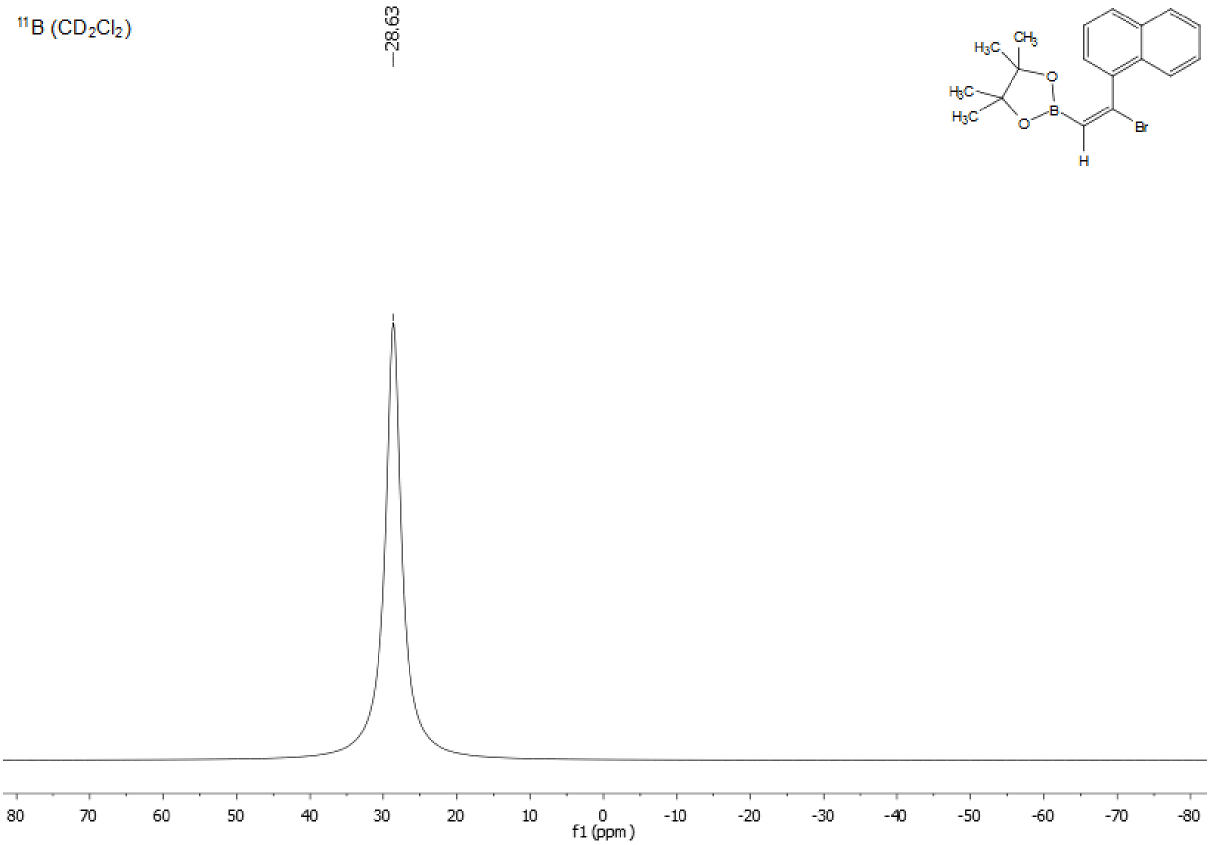

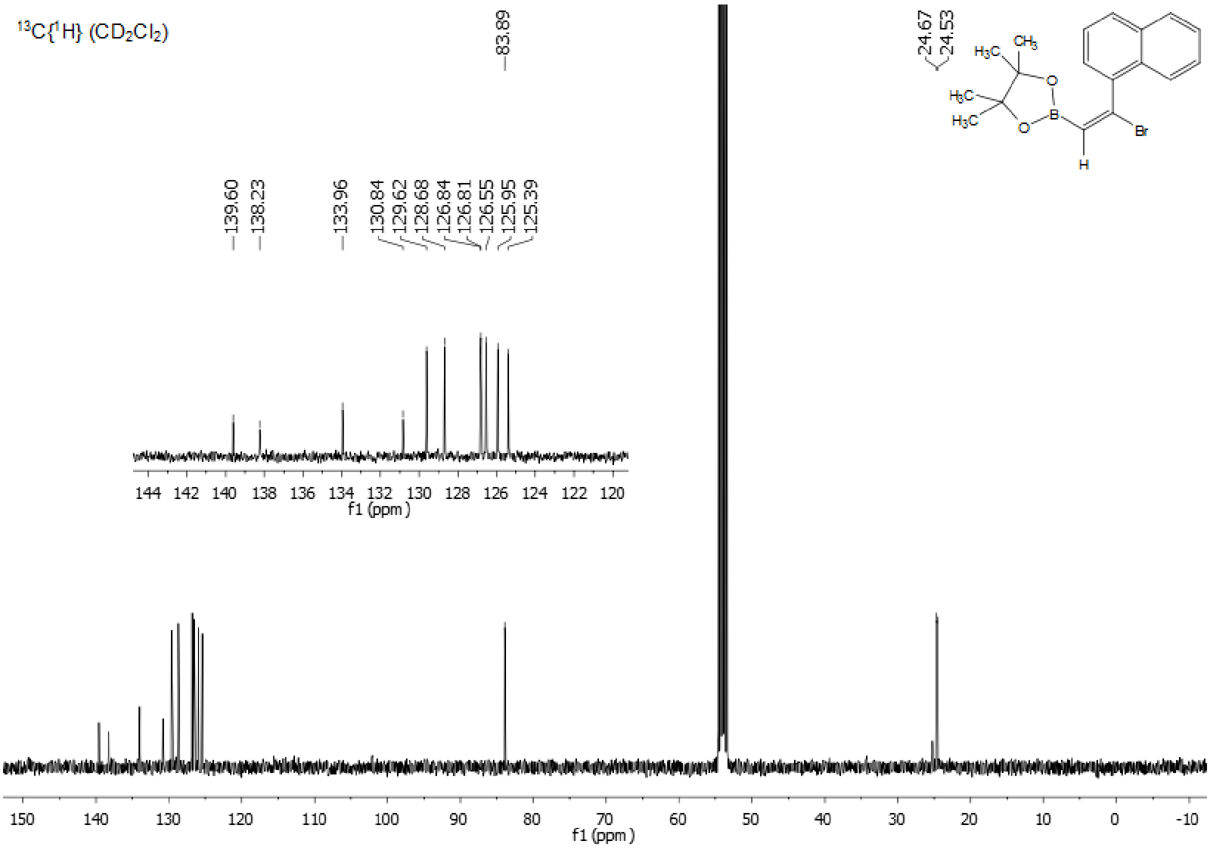
13C{1H} (CD2Cl2)
83.89
24.67
24.53
139.60
138.23
133.96
130.84
129.62
128.68
126.84
126.81
126.55
125.95
125.39
f1 (ppm)
H3C
CH3
H3C
H3C
O
O
B
Br
H

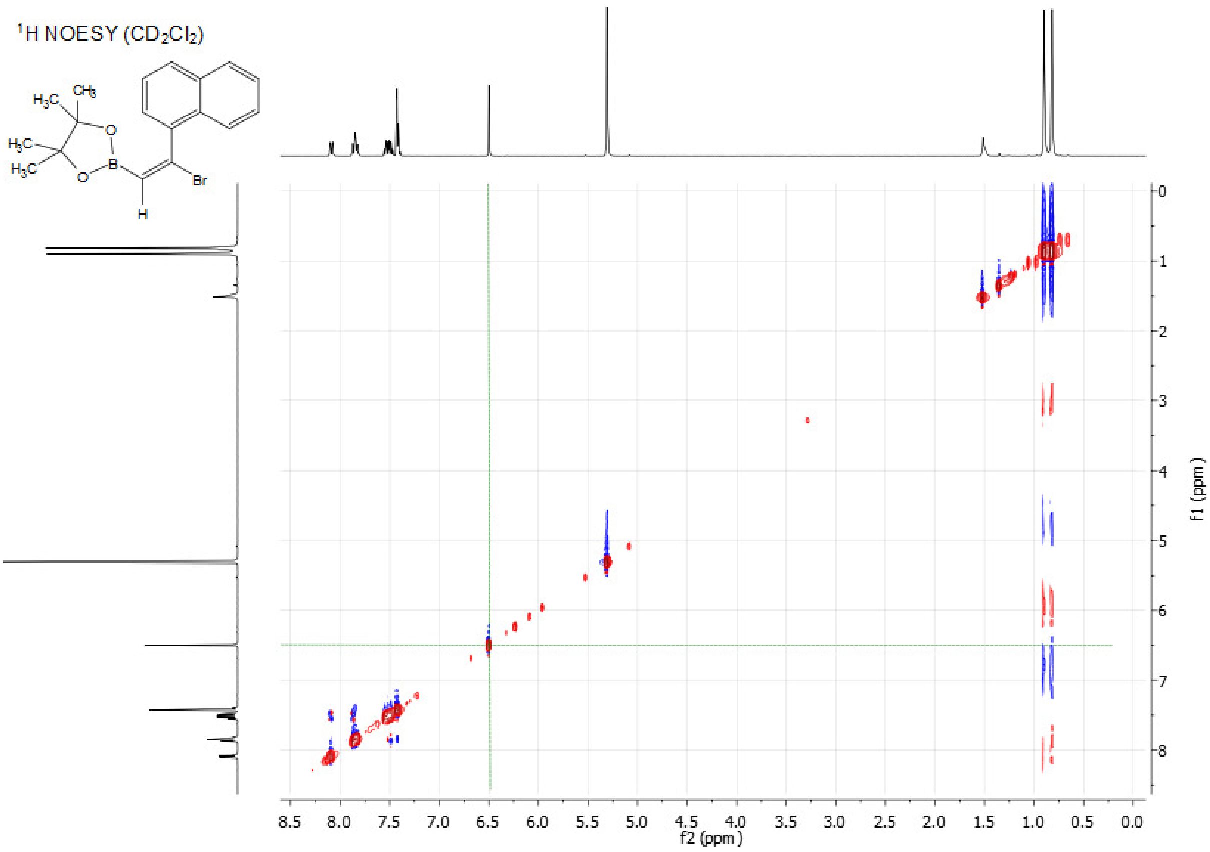
1H NOESY (CD2Cl2)
H3C
CH3
H3C
H3C
O
O
B
Br
H
f2 (ppm)
f1 (ppm)

1,3-dibromo-1H-naphtho[1,8-*bc*]borinine (**9**)

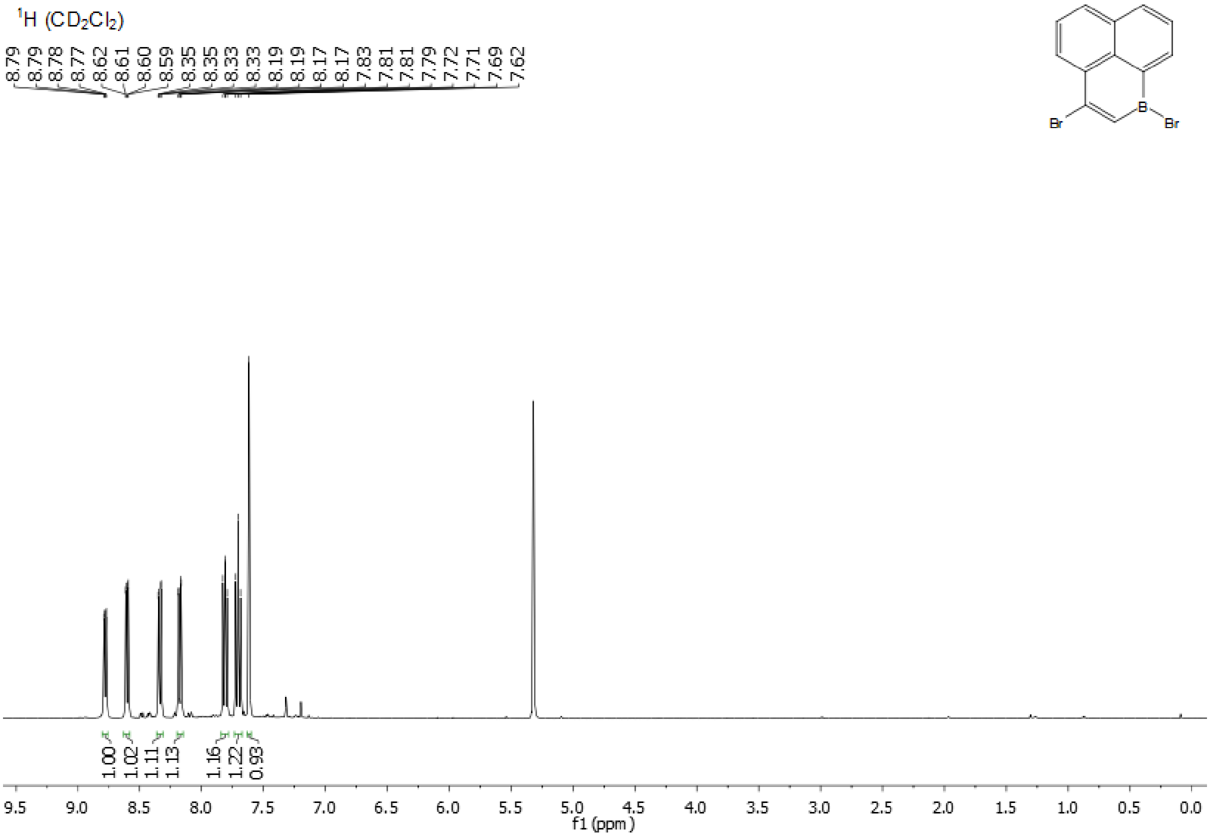

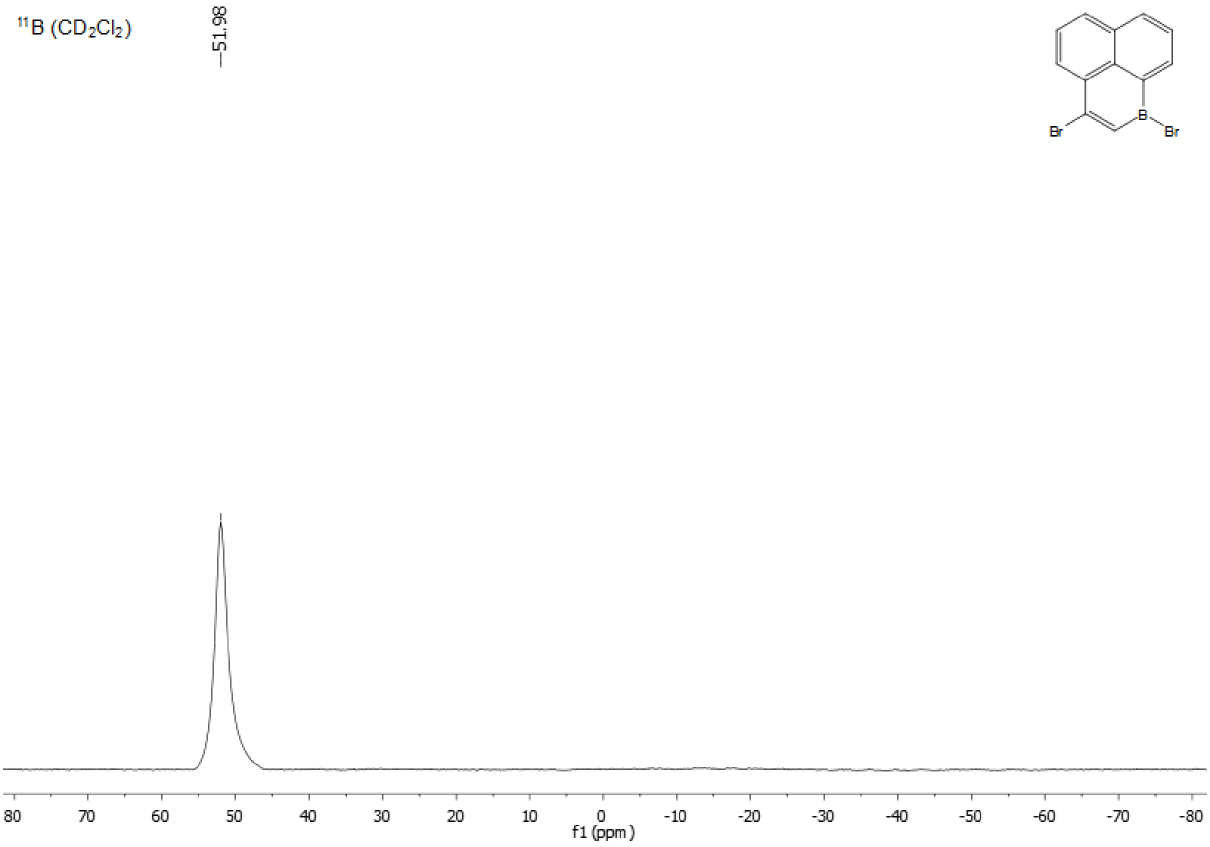

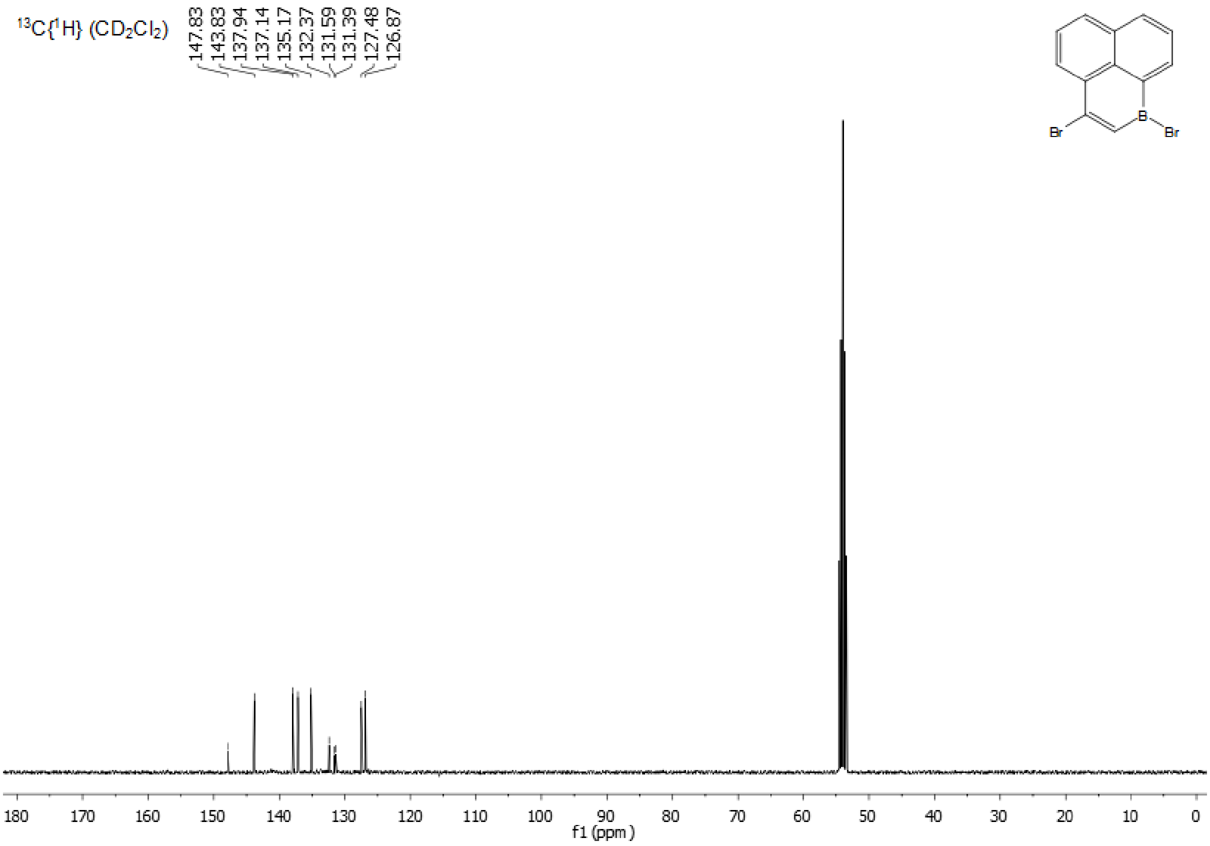

2-(8-ethynylnaphthalen-1-yl)-4,4,5,5-tetramethyl-1,3,2-dioxaborolane (**10a**)

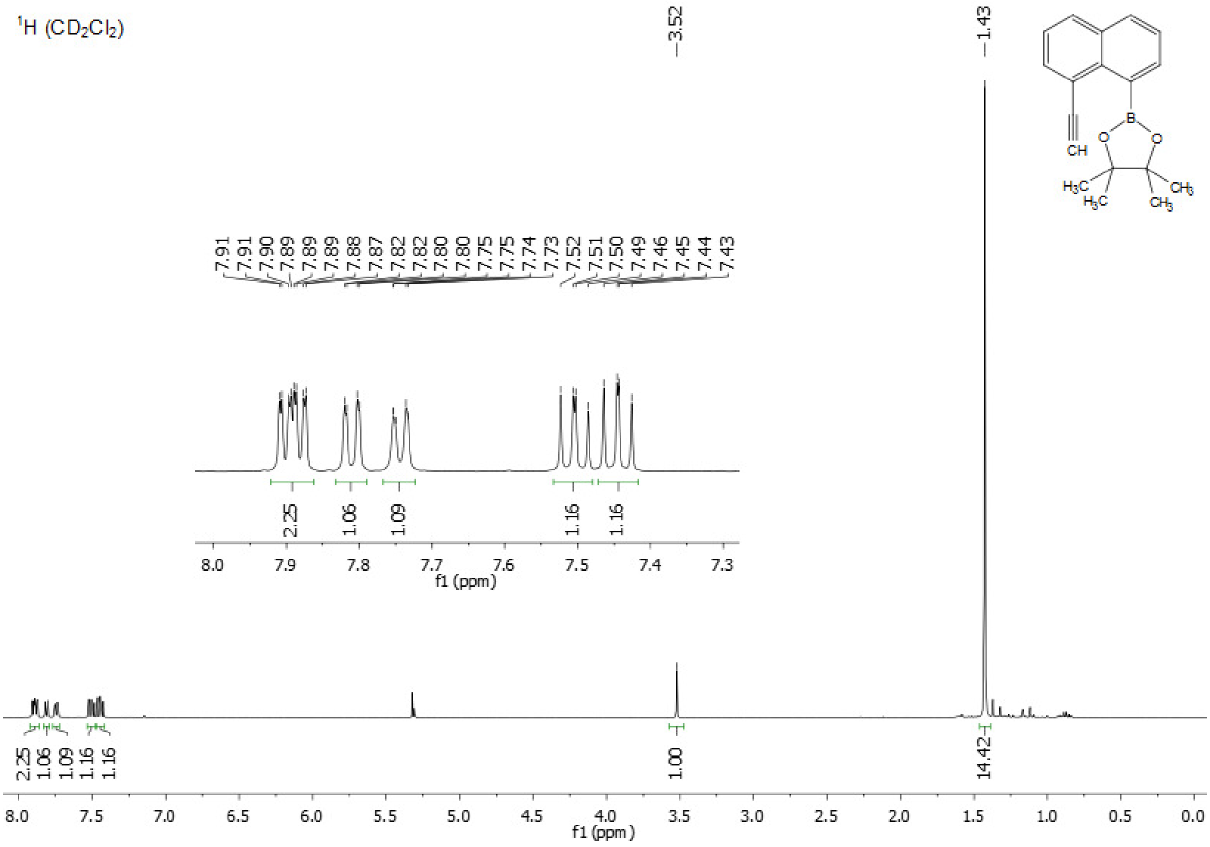

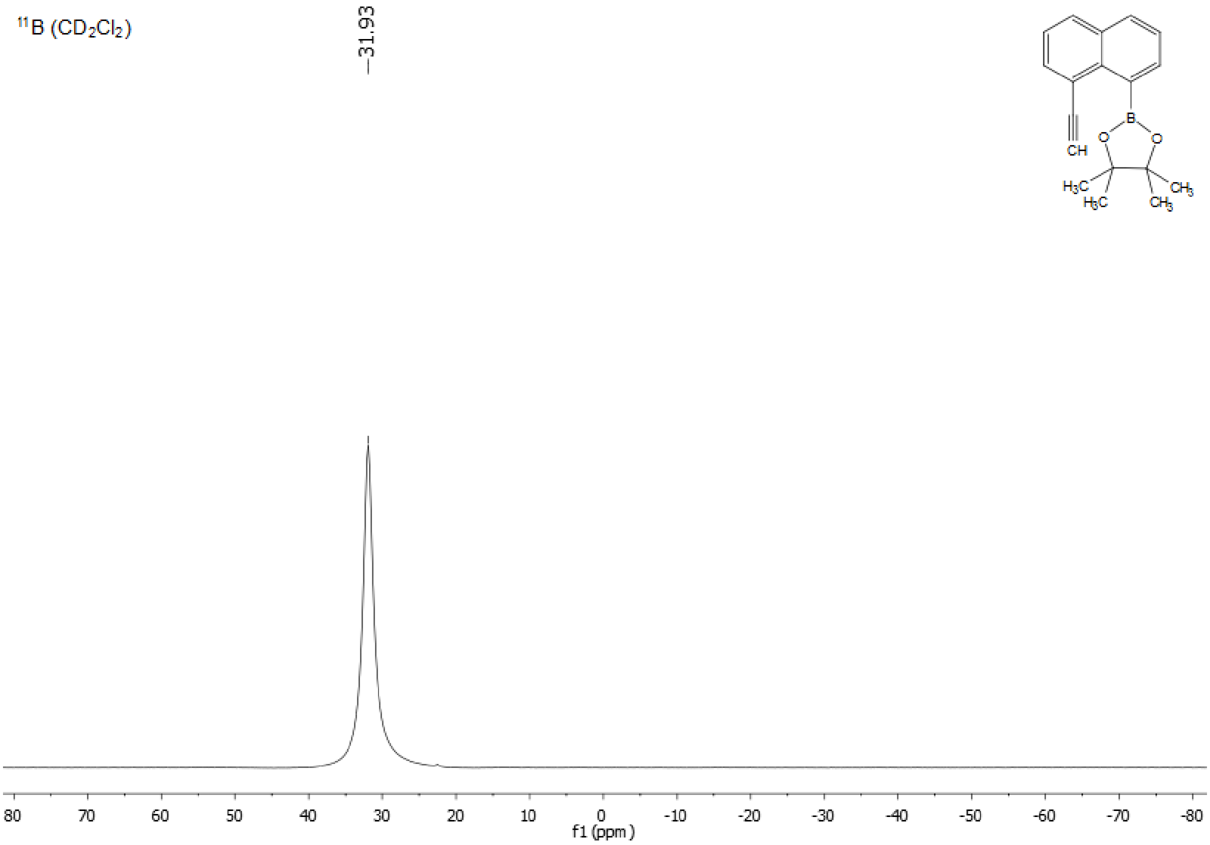

^{11}B (CD_2Cl_2)
31.93
80 70 60 50 40 30 20 10 0 -10 -20 -30 -40 -50 -60 -70 -80
f1 (ppm)
CH
B
O
O
H_3C
H_3C
CH_3
CH_3

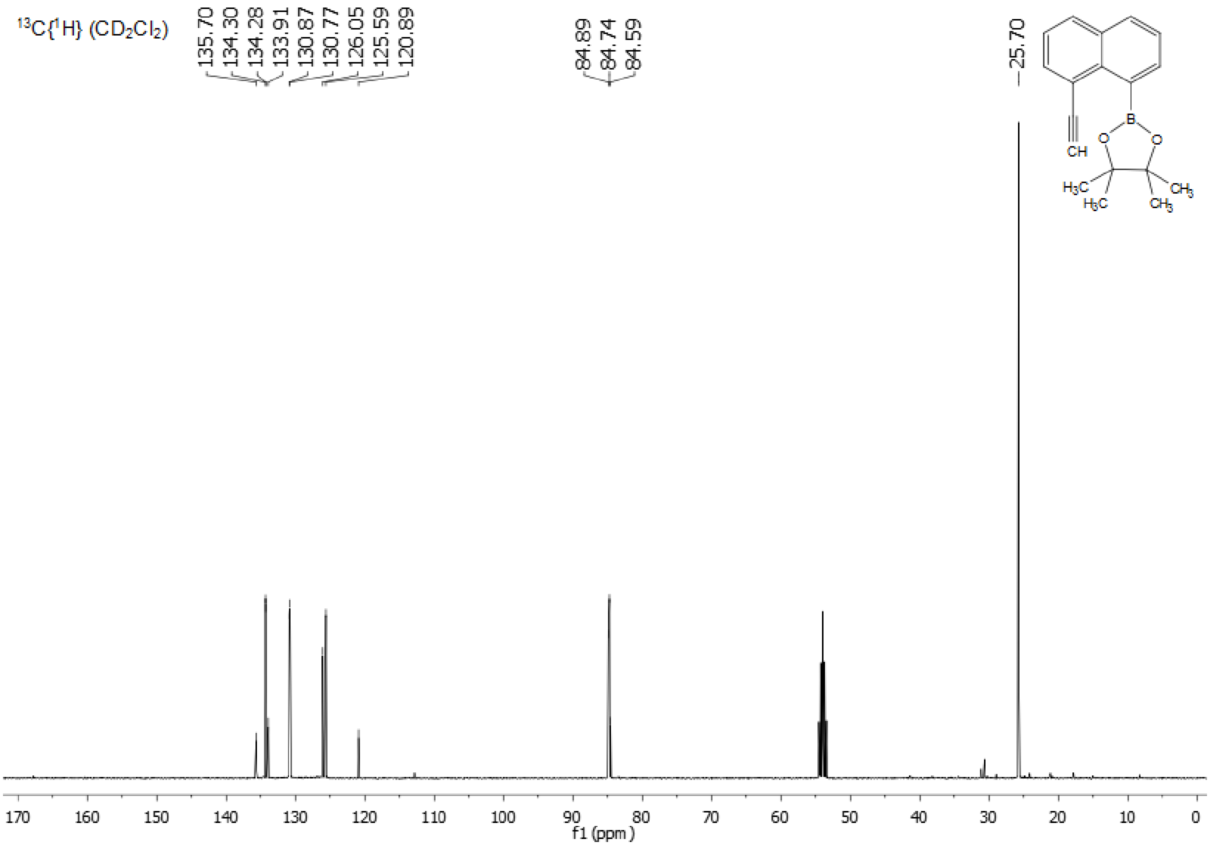

^{13}C{^{1}H} (CD_2Cl_2)
135.70
134.30
134.28
133.91
130.87
130.77
126.05
125.59
120.89
84.89
84.74
84.59
25.70
170 160 150 140 130 120 110 100 90 80 70 60 50 40 30 20 10 0
f1 (ppm)
CH
B
O
O
H_3C
H_3C
CH_3
CH_3

4,4,5,5-tetramethyl-2-(8-(pent-1-yn-1-yl)naphthalene-1-yl)-1,3,2-dioxaborolane (**10b**)

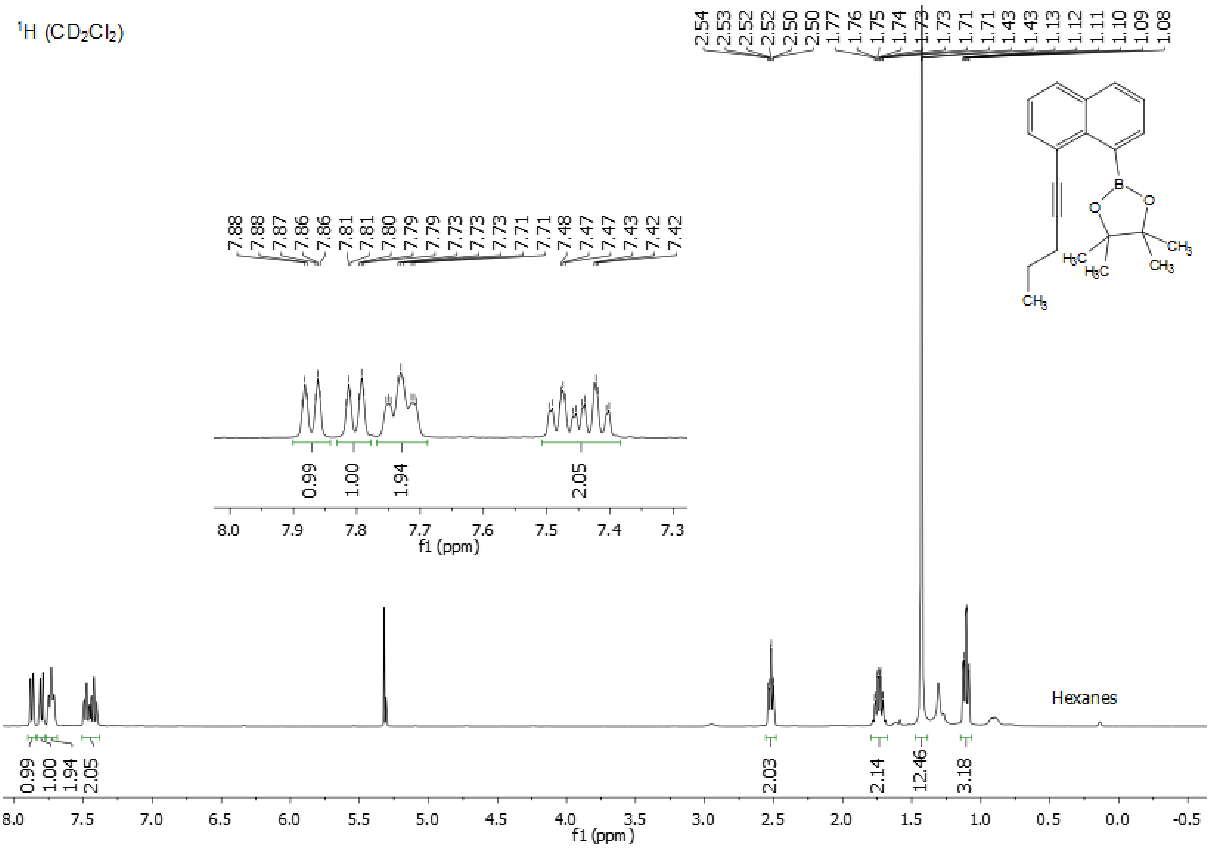

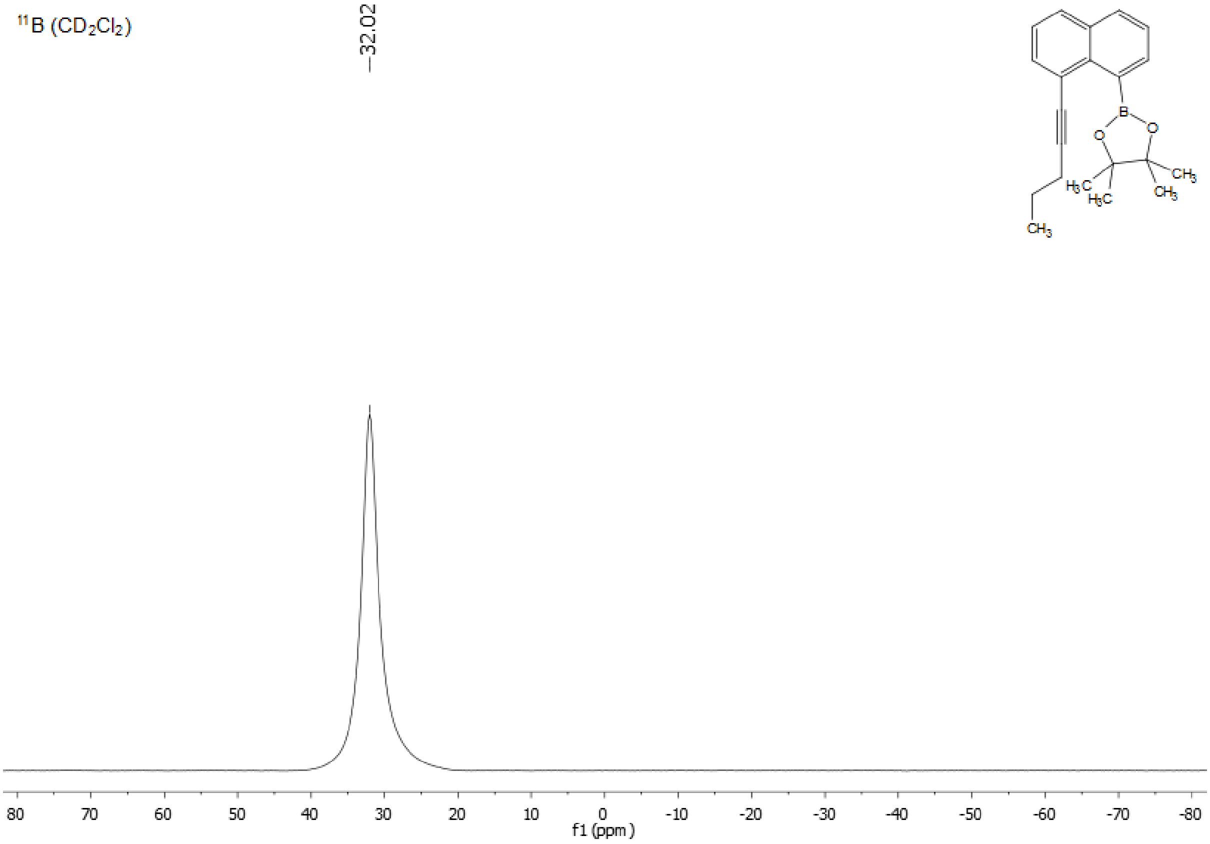

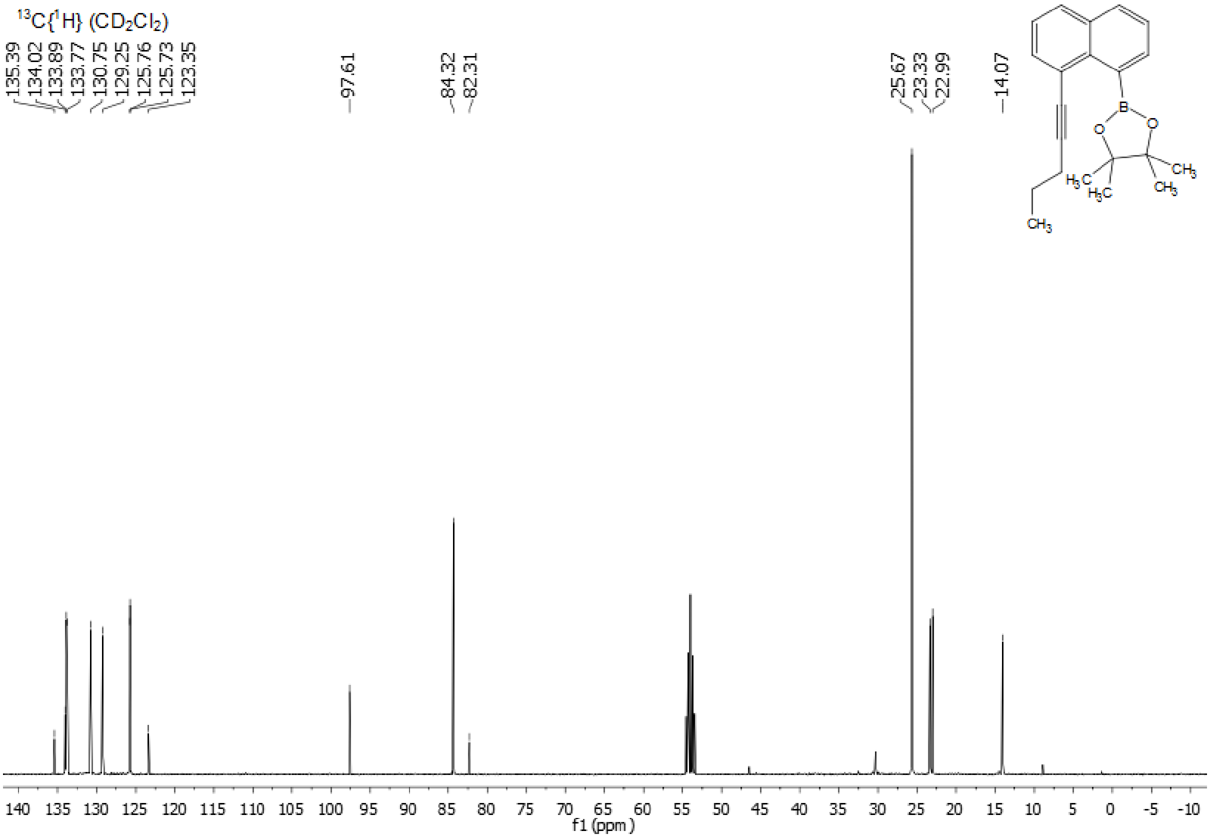

2-mesityl-3-(p-tolyl)-1H-naphtho[1,8-bc]borinin-1-ol (**11**)

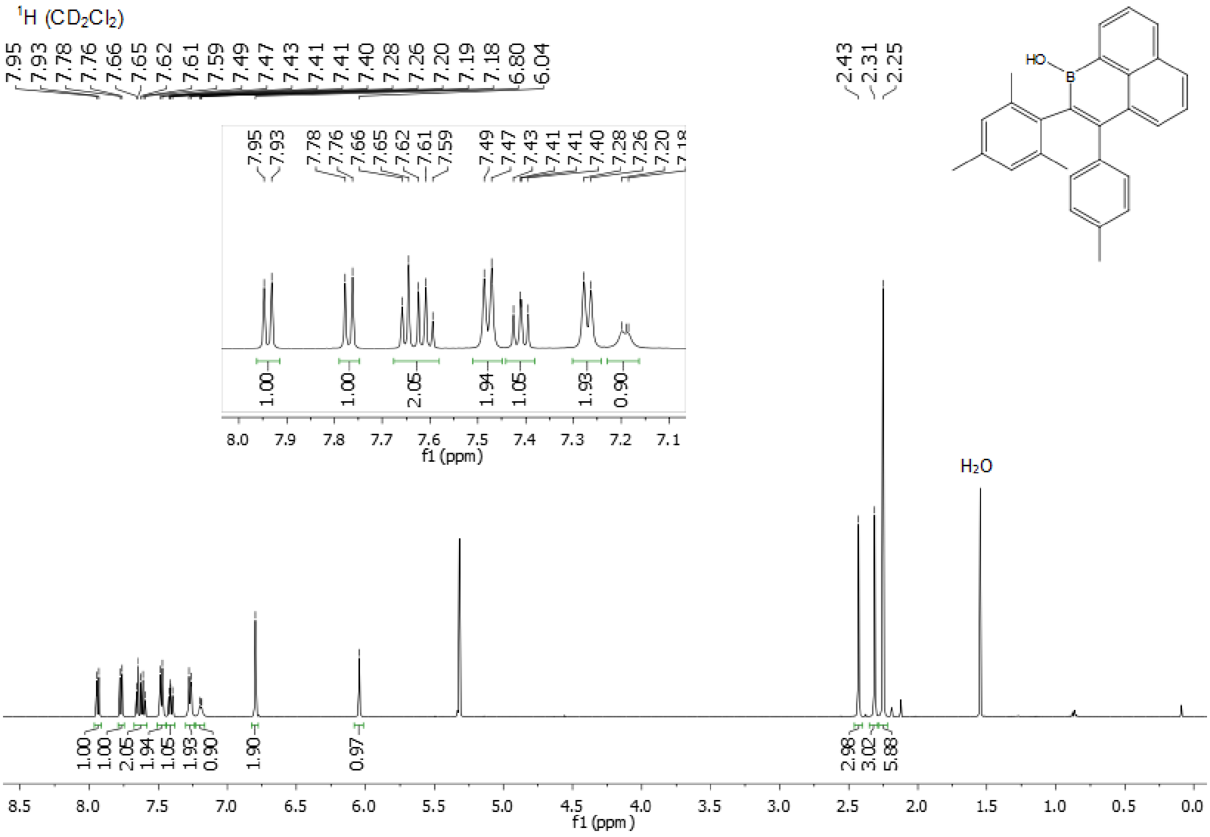

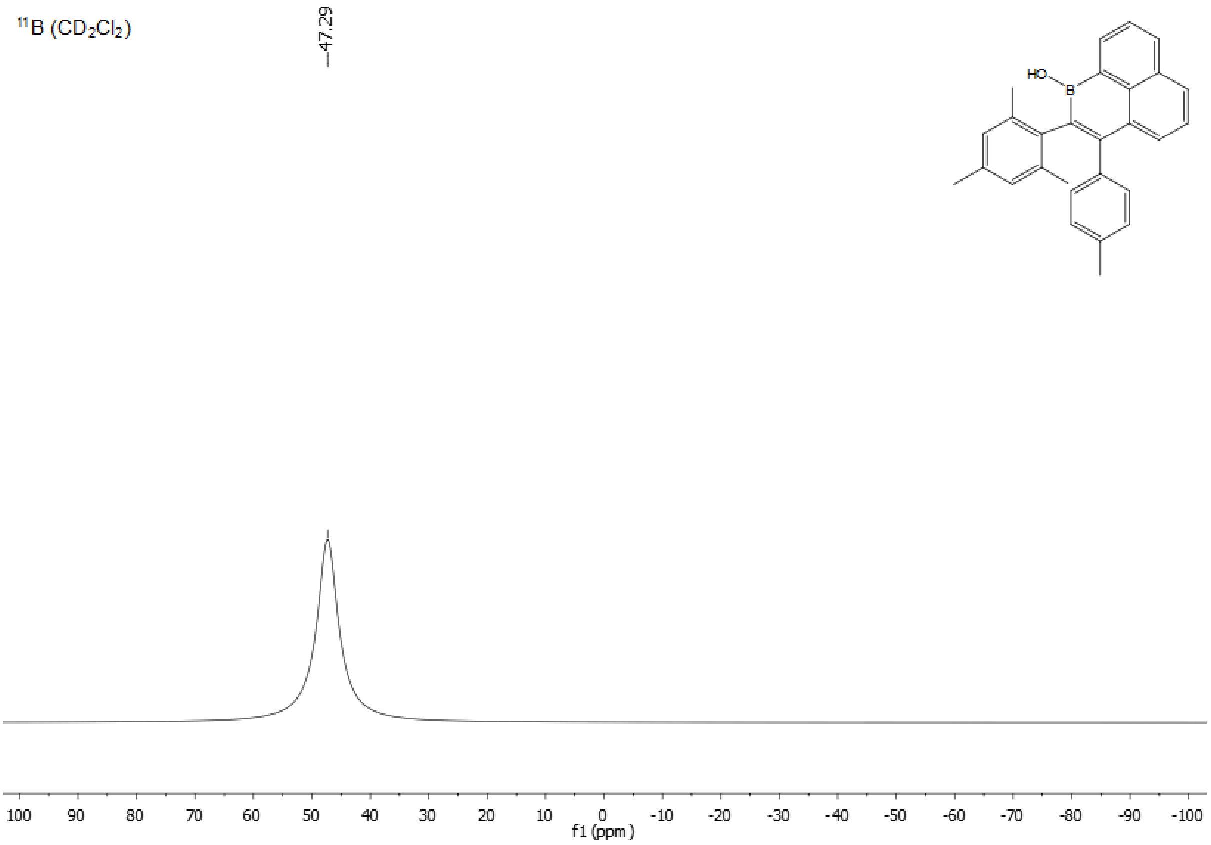
11B (CD2Cl2)
47.29
HO
B
100 90 80 70 60 50 40 30 20 10 0 -10 -20 -30 -40 -50 -60 -70 -80 -90 -100
f1 (ppm)

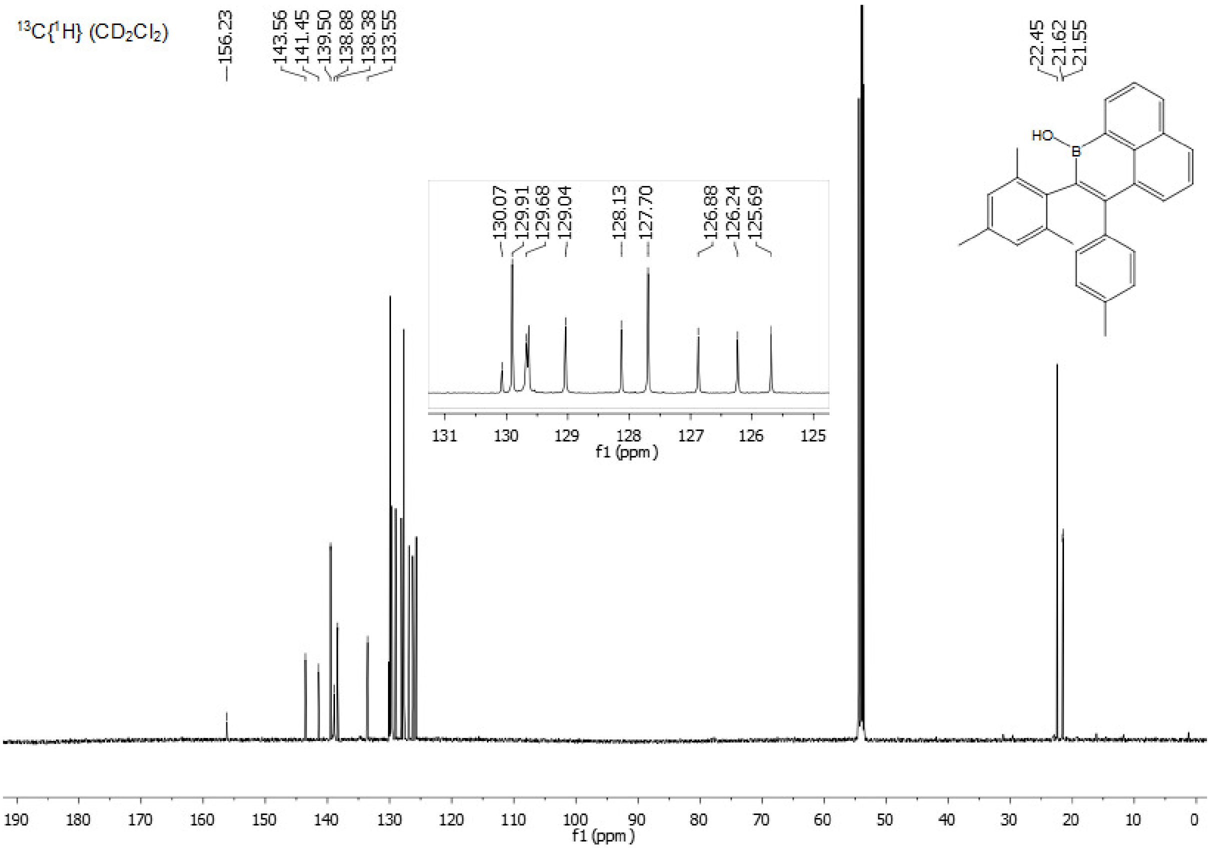
13C{1H} (CD2Cl2)
156.23
143.56
141.45
139.50
138.88
138.38
133.55
22.45
21.62
21.55
130.07
129.91
129.68
129.04
128.13
127.70
126.88
126.24
125.69
131 130 129 128 127 126 125
f1 (ppm)
HO
B
190 180 170 160 150 140 130 120 110 100 90 80 70 60 50 40 30 20 10 0
f1 (ppm)

2-bromo-1-mesityl-3-(p-tolyl)-1H-naphtho[1,8-bc]borinine (**12**)

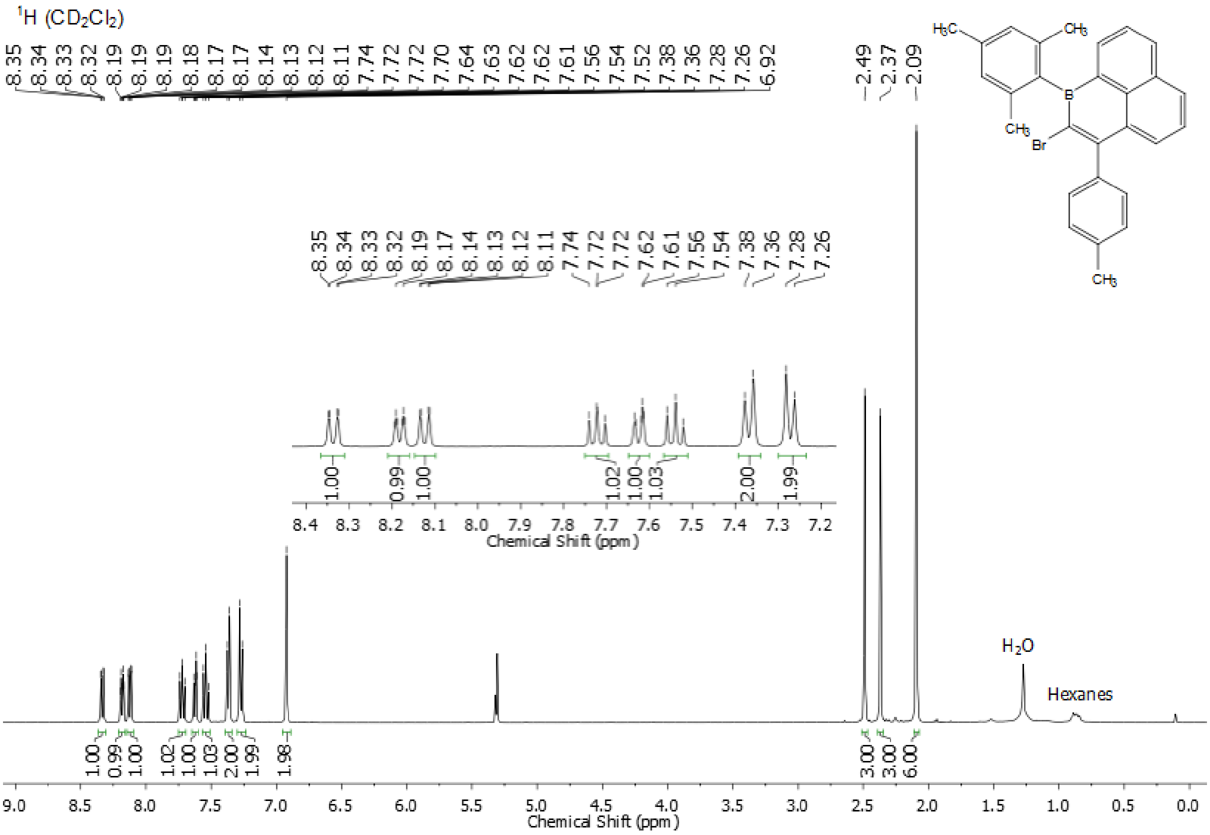

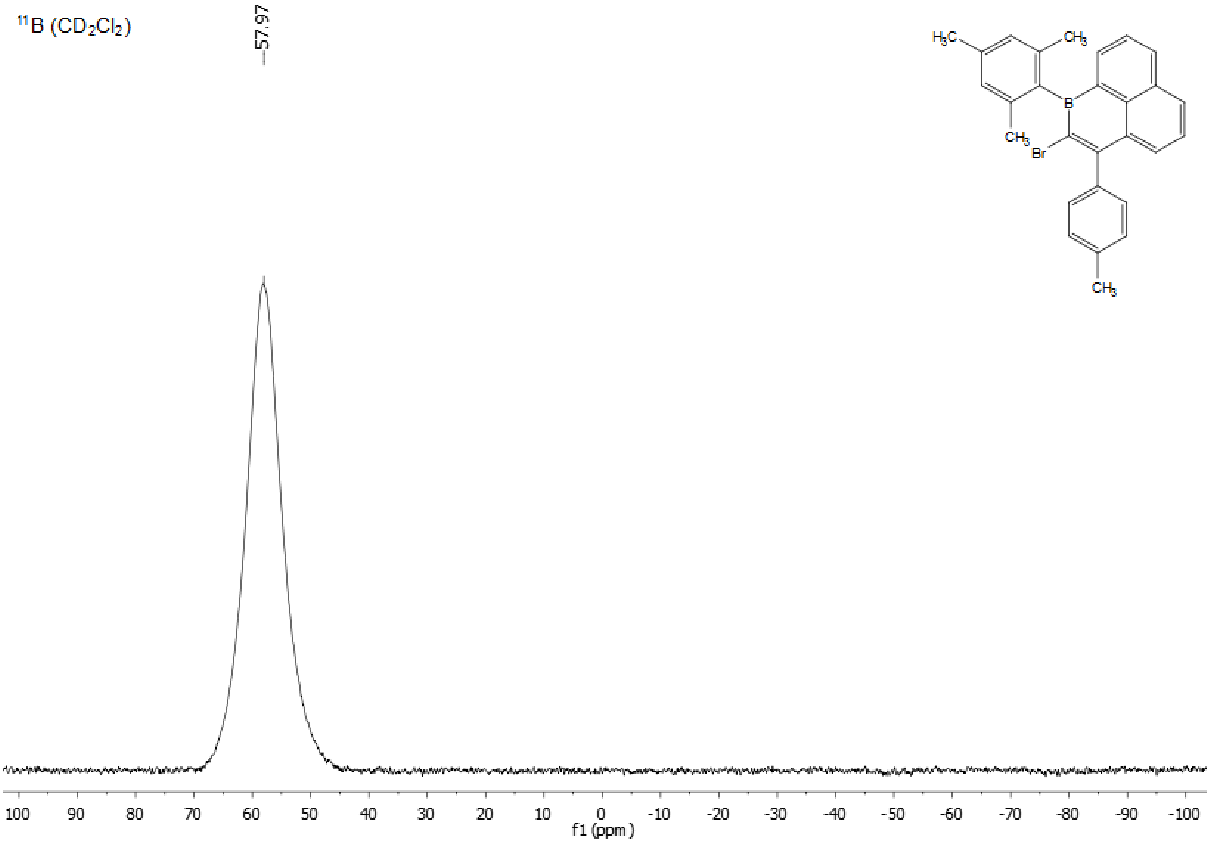

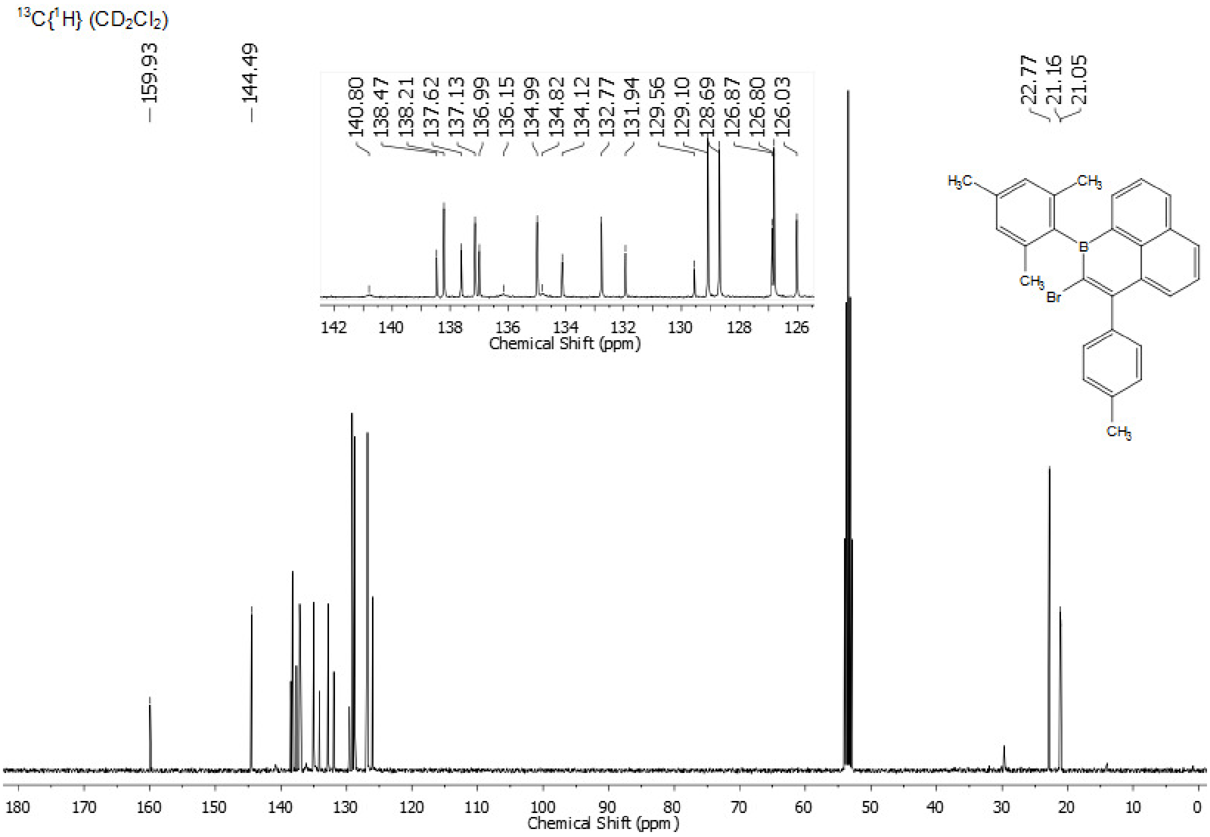

1,2-dimesityl-3-(p-tolyl)-1H-naphtho[1,8-*bc*]borinine (**13**)

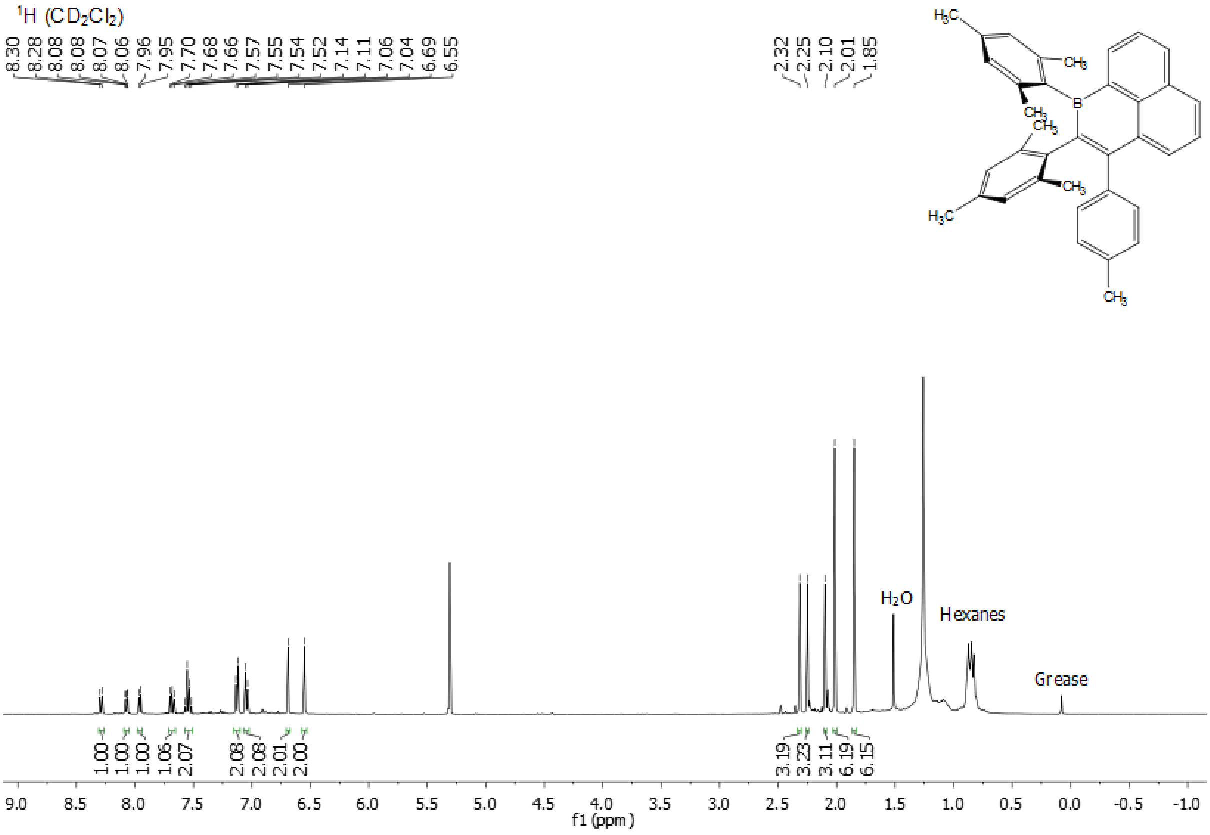

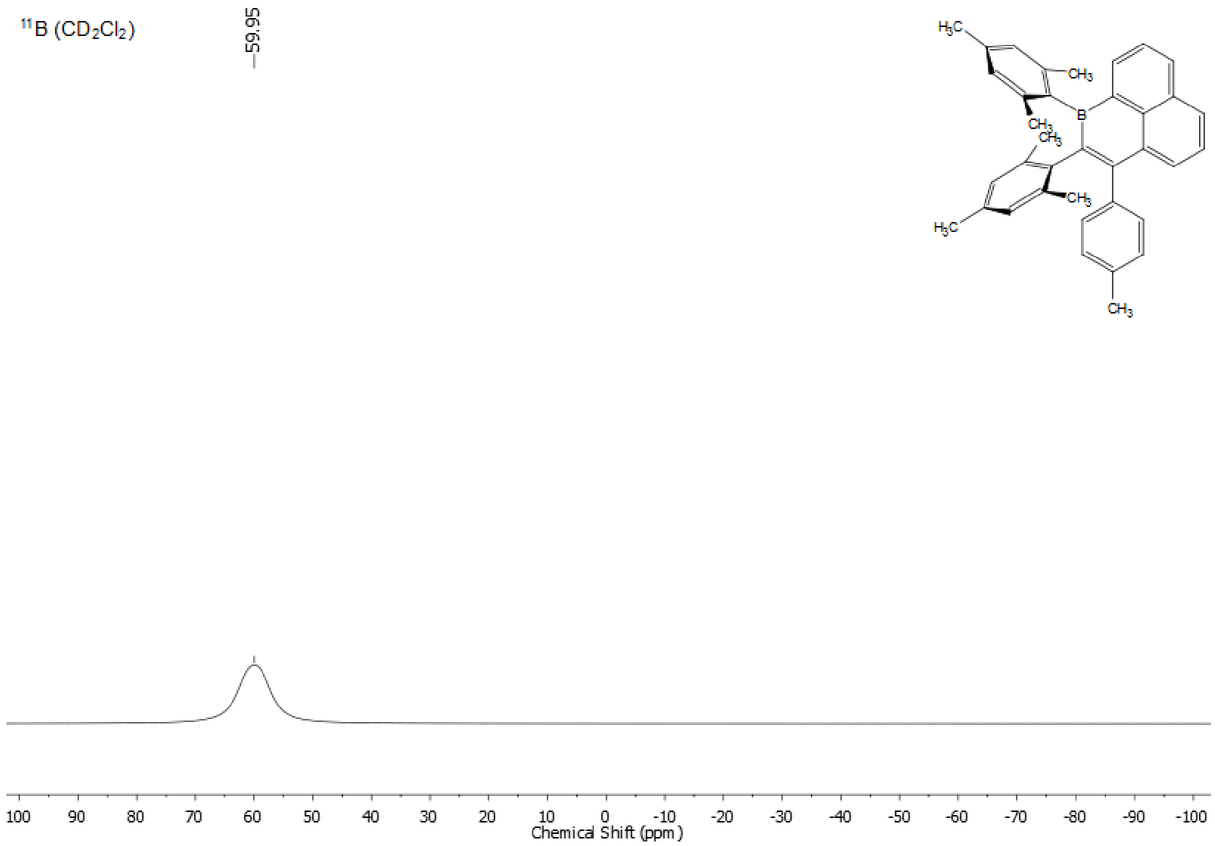

11B (CD2Cl2)
59.95
H3C
CH3
CH3
CH3
B
CH3
H3C
CH3
100 90 80 70 60 50 40 30 20 10 0 -10 -20 -30 -40 -50 -60 -70 -80 -90 -100
Chemical Shift (ppm)

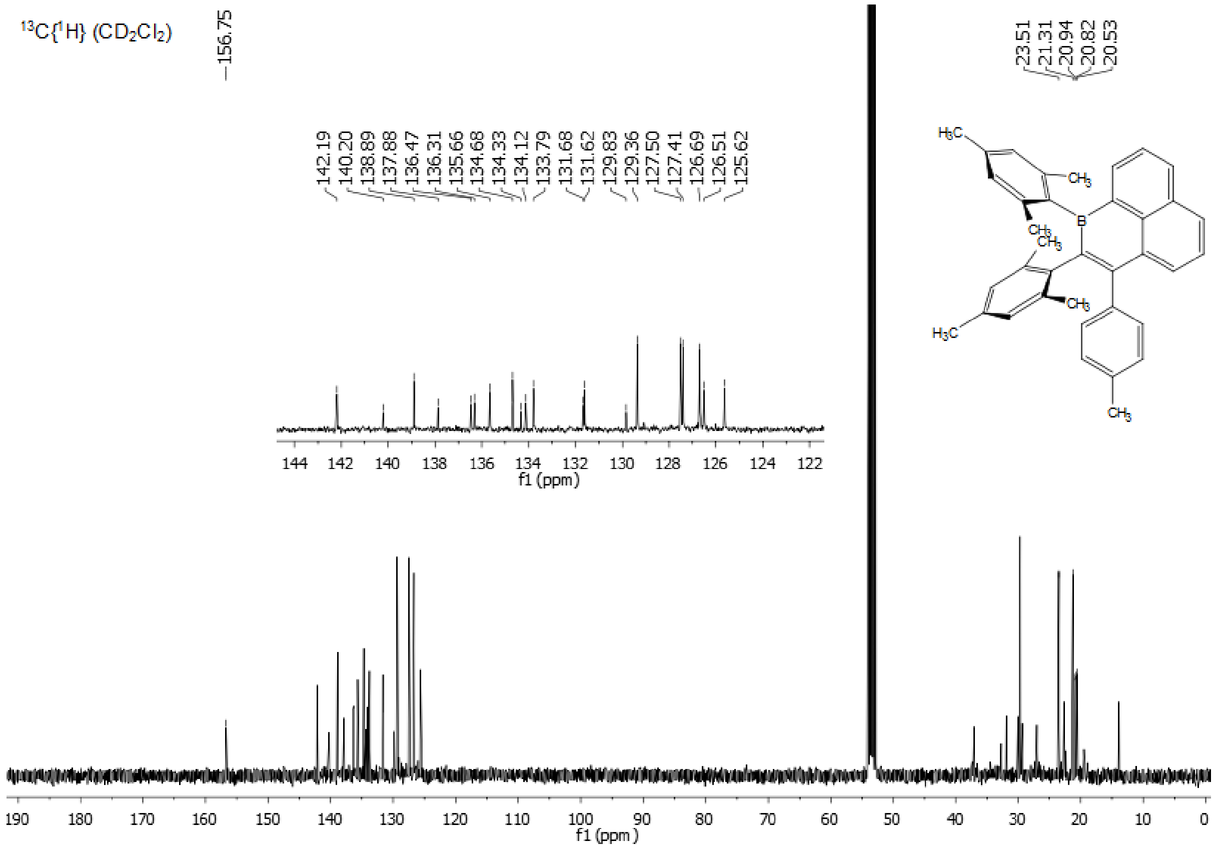

13C{1H} (CD2Cl2)
156.75
142.19
140.20
138.89
137.88
136.47
136.31
135.66
134.68
134.33
134.12
133.79
131.68
131.62
129.83
129.36
127.50
127.41
126.69
126.51
125.62
23.51
21.31
20.94
20.82
20.53
144 142 140 138 136 134 132 130 128 126 124 122
f1 (ppm)
H3C
CH3
CH3
CH3
B
CH3
H3C
CH3
190 180 170 160 150 140 130 120 110 100 90 80 70 60 50 40 30 20 10 0
f1 (ppm)

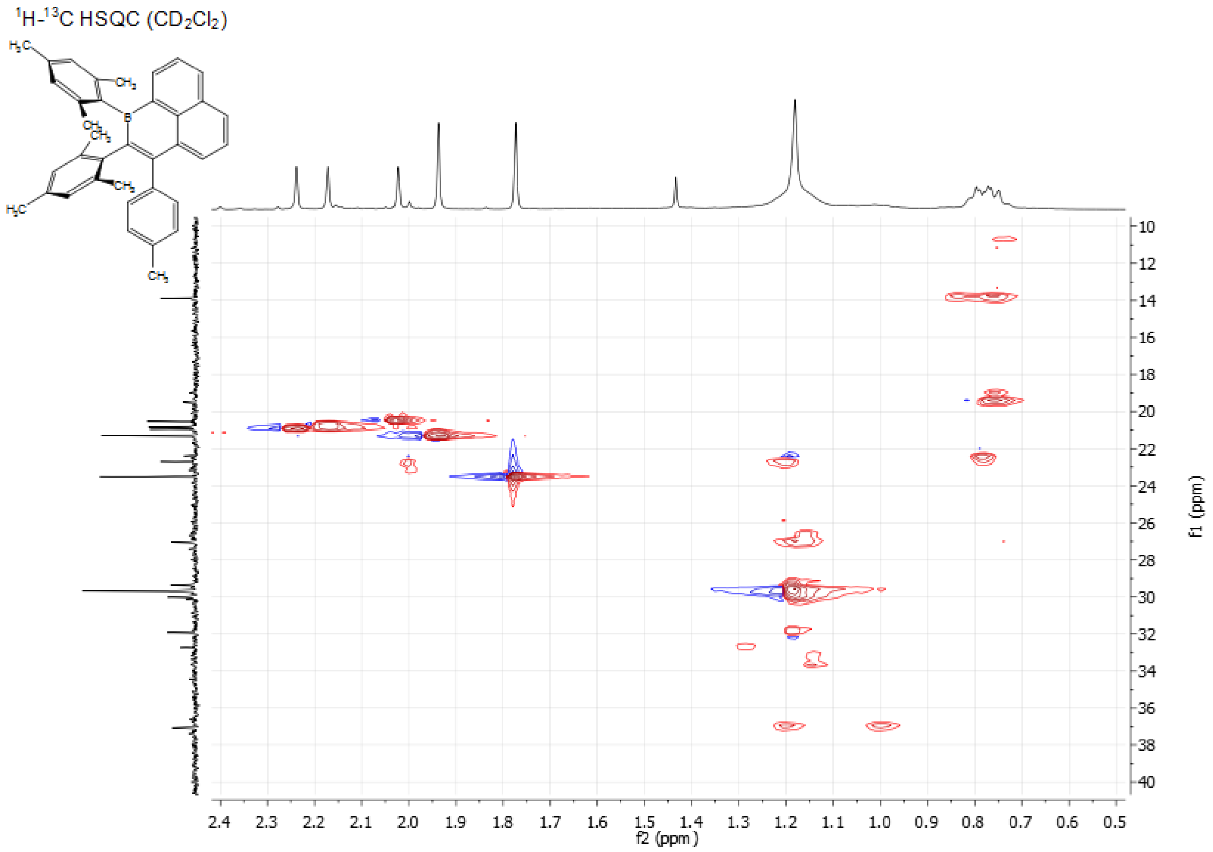

1-mesityl-3-(p-tolyl)-1H-naphtho[1,8-bc]borinine (**14**)

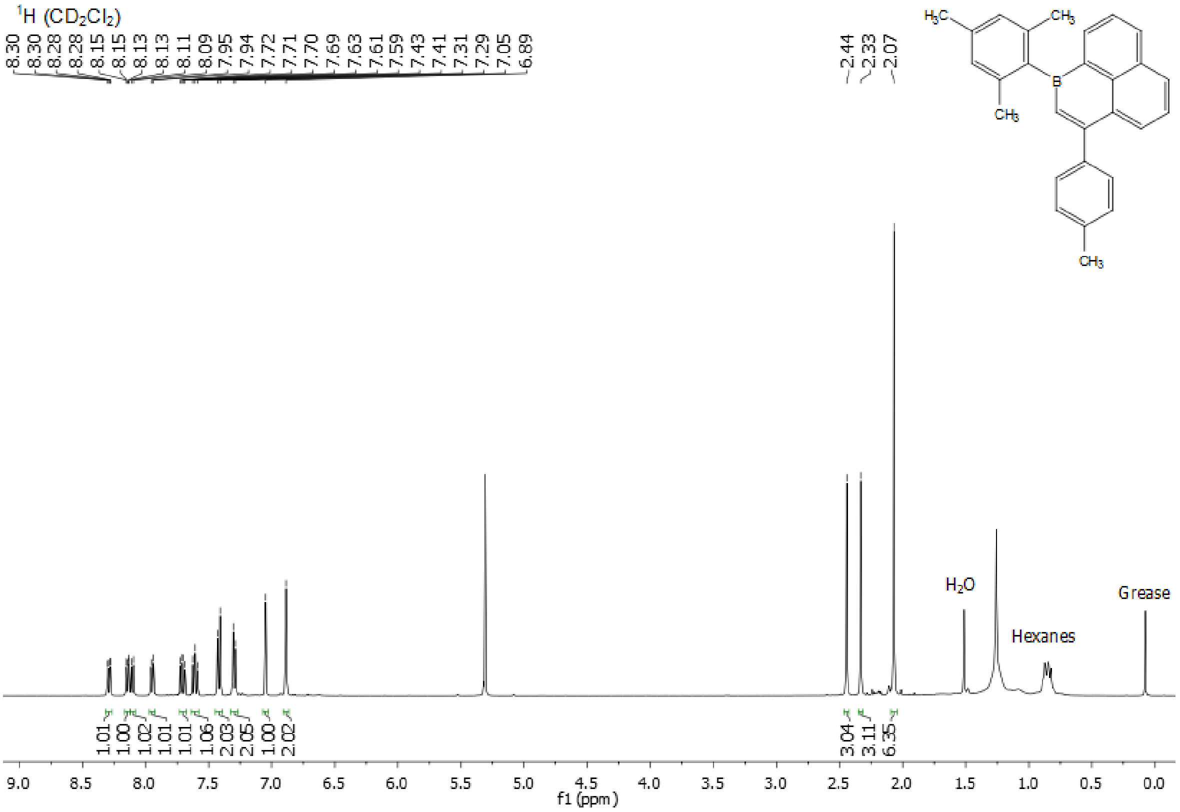

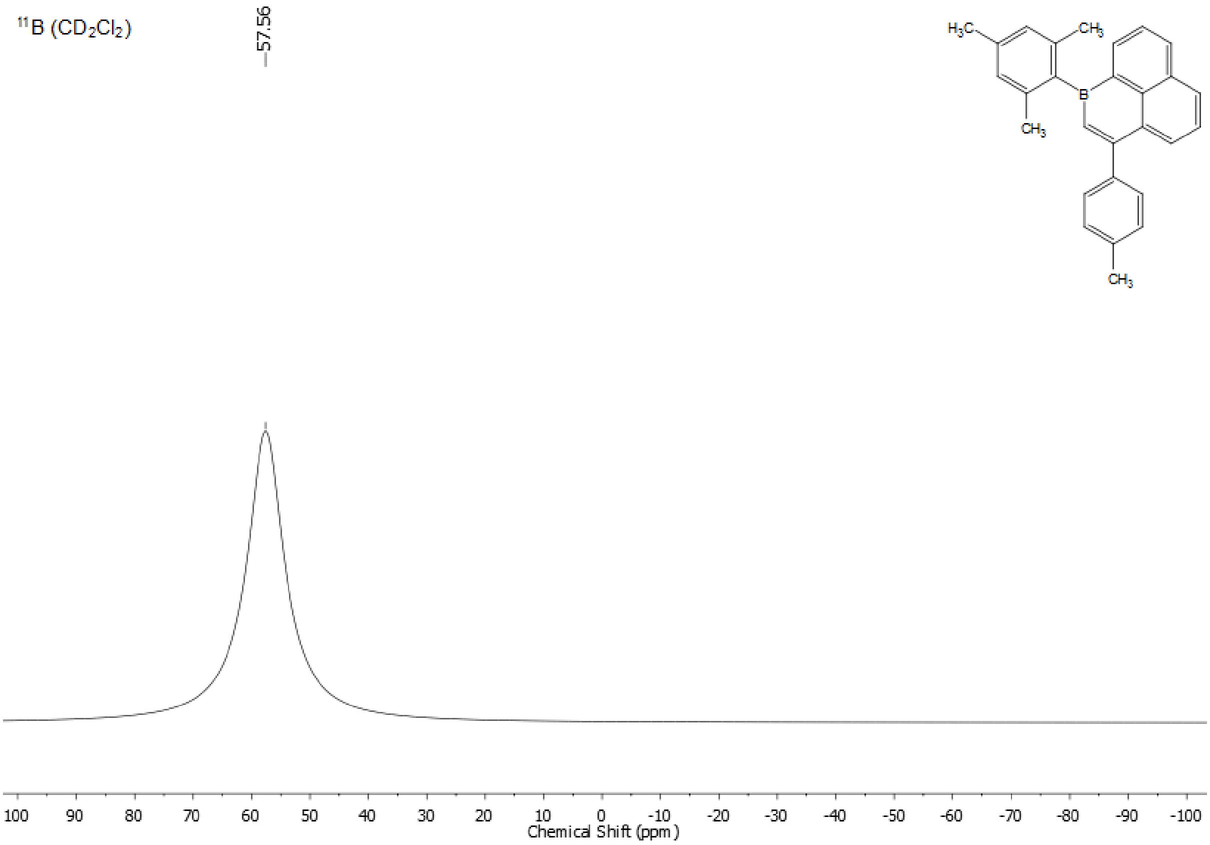
11B (CD2Cl2)
57.56
H3C
CH3
CH3
B
CH3
Chemical Shift (ppm)

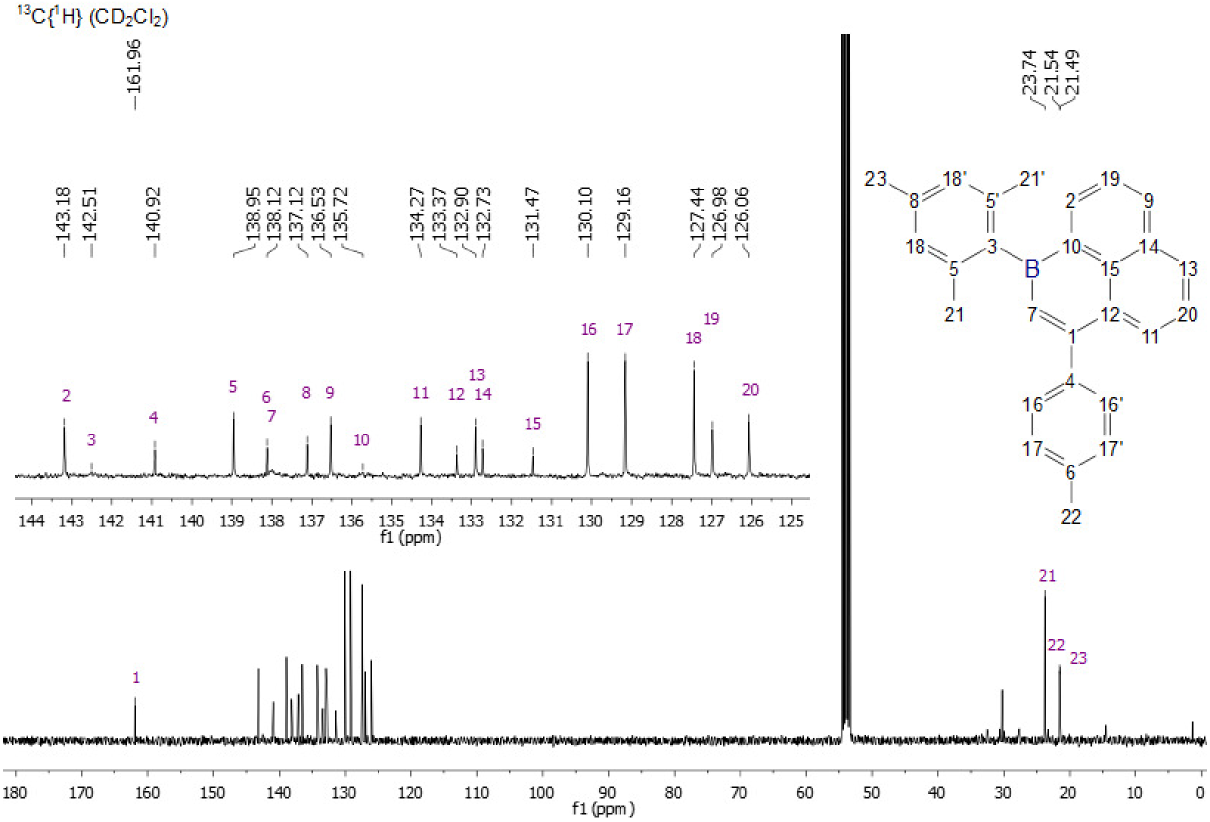
13C{1H} (CD2Cl2)
161.96
143.18
142.51
140.92
138.95
138.12
137.12
136.53
135.72
134.27
133.37
132.90
132.73
131.47
130.10
129.16
127.44
126.98
126.06
23.74
21.54
21.49
f1 (ppm)
f1 (ppm)

^1H-^{13}C HSQC (CD_2Cl_2)

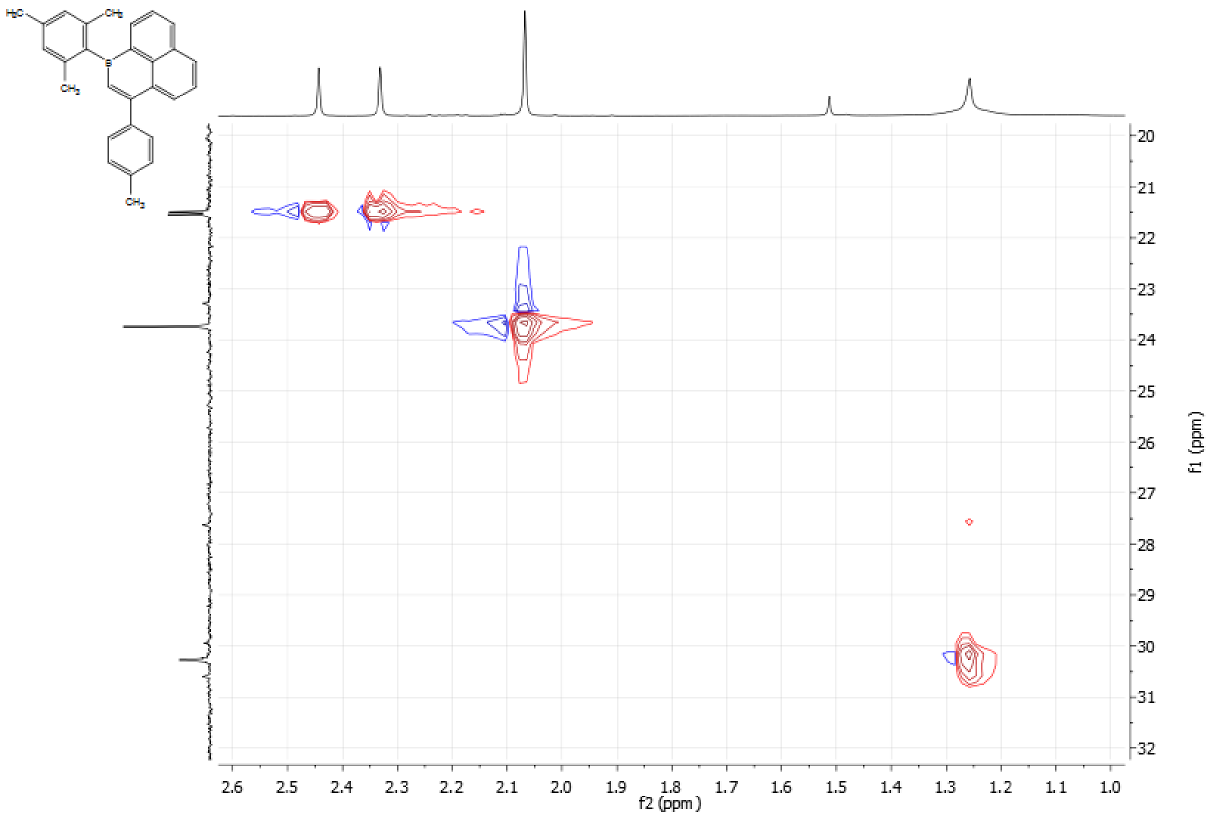

3-bromo-1-mesityl-1H-naphtho[1,8-bc]borinine (**15**)

^1H ($CDCl_3$)

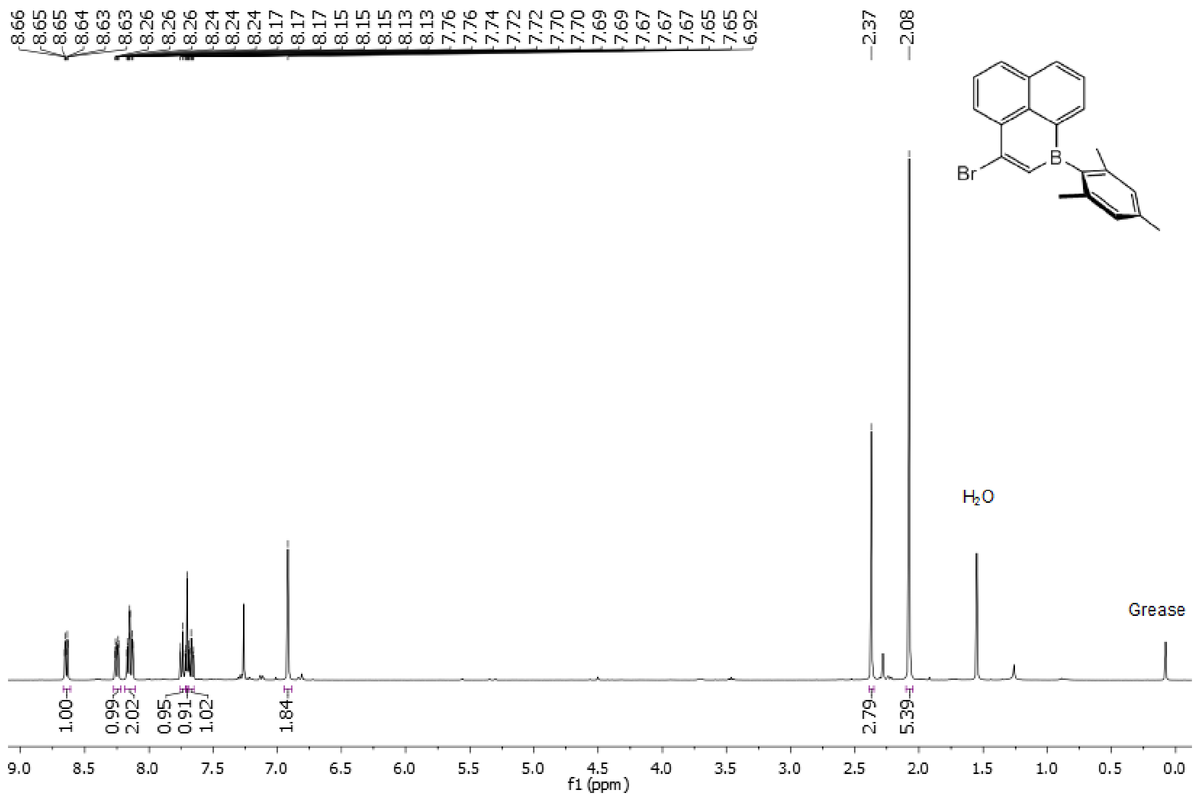

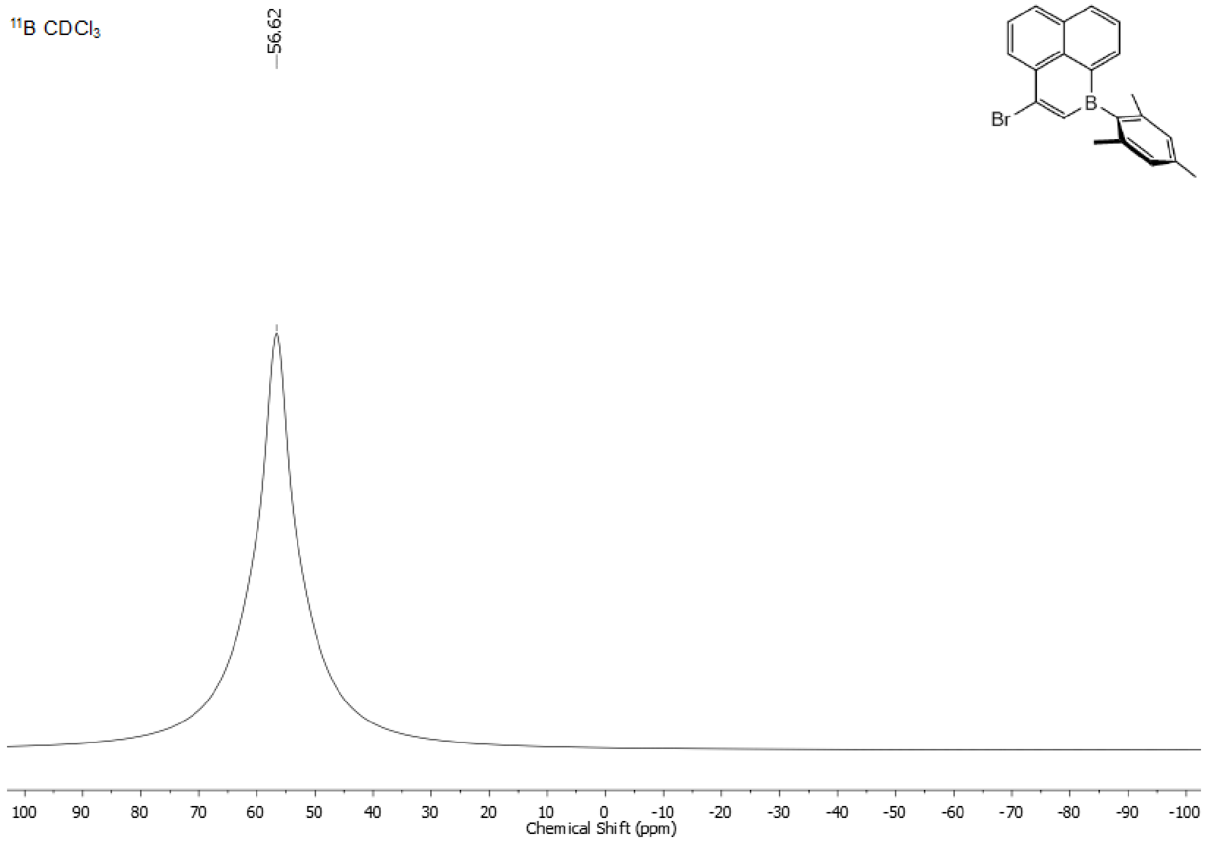

^{11}B CDCl$_3$
56.62
Br
B
Chemical Shift (ppm)

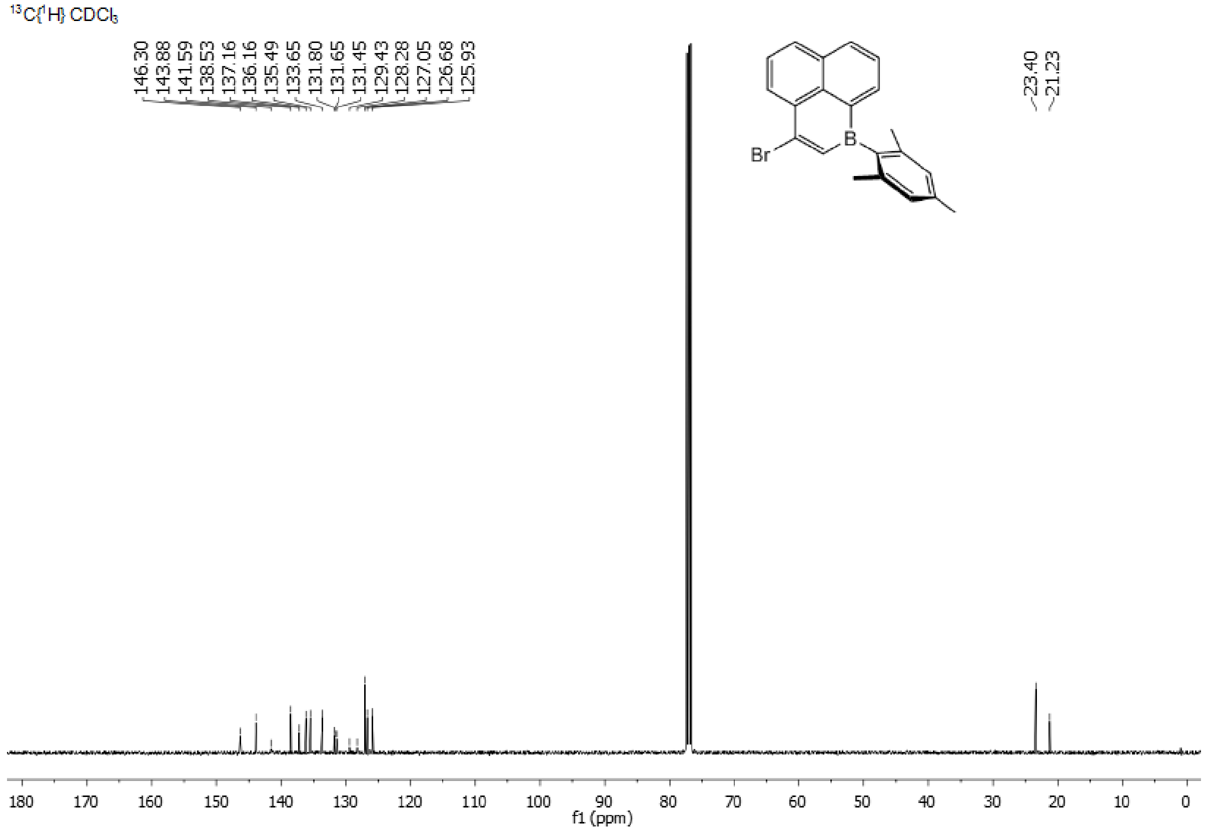

^{13}C{^{1}H} CDCl$_3$
146.30
143.88
141.59
138.53
137.16
136.16
135.49
133.65
131.80
131.65
131.45
129.43
128.28
127.05
126.68
125.93
23.40
21.23
Br
B
f1 (ppm)

Crystallographic Details and Extended Packing Structures

Data for compounds **6a** and **6c** were recorded on an Agilent Supernova diffractometer, with Mo Kα radiation (mirror monochromator, λ = 0.7107). Data for **8-BPin**, **9**, and **10a** were recorded on an Oxford Xcalibur2 diffractometer, with Mo Kα radiation (mirror monochromator, λ = 0.7107). Data for **12** were recorded on a dual source Rigaku FR-X rotating anode diffractometer equipped with a HyPix 6000 HE detector and an Oxford Cryostream 700 plus, with Cu Kα radiation (mirror monochromator, λ = 1.54184). The CrysAlisPro[8] software package was used for data collection, cell refinement, data reduction and absorption correction, which were applied using spherical harmonics, implemented in the SCALE3 ABSPACK scaling algorithm. Structure **6a** was solved using Olex2.solve;[9] structure **10a** was solved using Superflip;[10] and structures **6c**, **8-BPin**, **9** and **12** was solved using ShelXT.[11] All structures were solved and refined using ShelXL.[12] Non-hydrogen atoms were refined anisotropically. Hydrogen atoms were all located in a difference map and repositioned geometrically. The data for **9** showed substitutional disorder of the carbon and boron atoms with bromine substituents (B1 and C2). These atoms were constrained to have the same positional parameters using EXYZ and EADP commands. Crystallographic data have been deposited with the Cambridge Crystallographic Data Centre under references 1535809 – 1535813 and 1827898. These data can be obtained free of charge from The Cambridge Crystallographic Data Centre via www.ccdc.cam.ac.uk/data_request/cif.

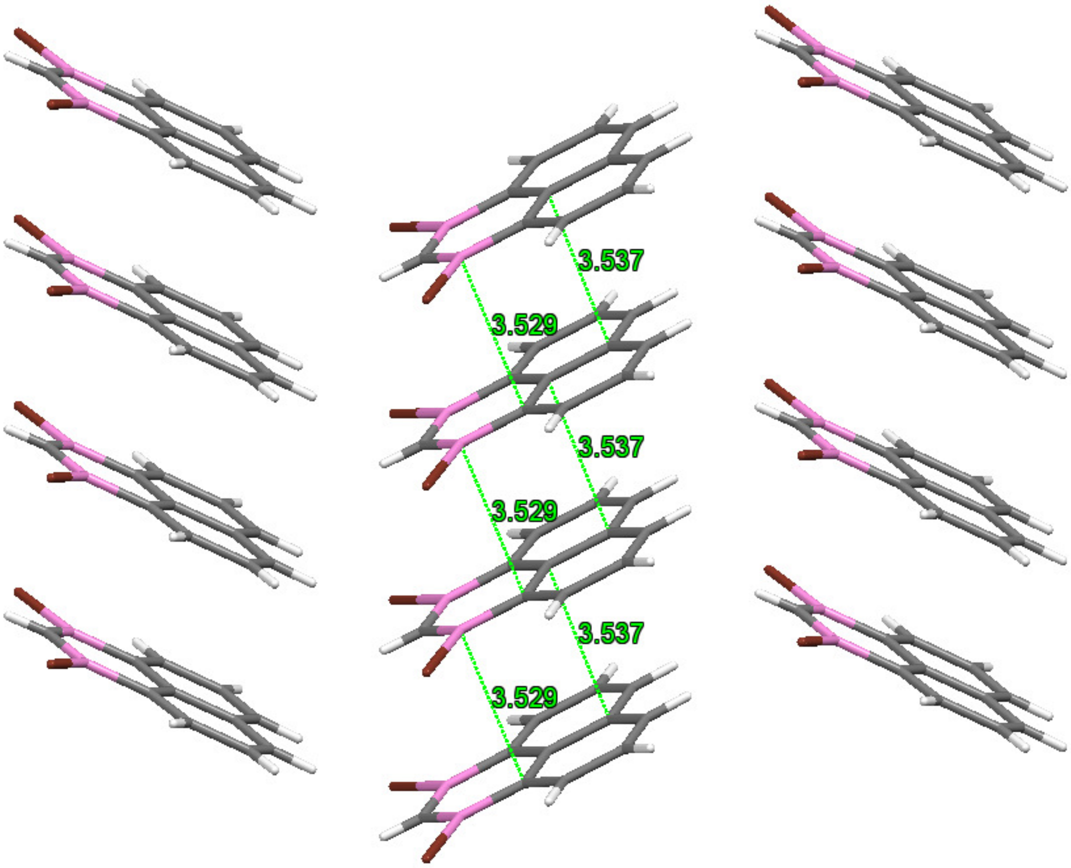

Figure S1: Extended packing structure for **9** showing the close C---C and C---B intermolecular contacts in adjacent molecules.

Table S1. Crystal Structure Refinement Data

	**6a**	**6c**	**8-BPin**	**9**	**10a**	**12**
Empirical Formula	$C_{30}H_{20}BBrO$	$C_{19}H_{14}BBrO$	$C_{18}H_{20}BBrO_2$	$C_{12}H_7BBr_2$	$C_{18}H_{19}BO_2$	$C_{28}H_{24}BBr\cdot(CD_2Cl_2)_{0.25}$
Fw/g mol^{-1}	487.18	349.02	359.06	321.99	278.17	471.92
Crystal system Space Group	Triclinic P^{-1}	Triclinic P^{-1}	Monoclinic P21/c	Orthorhombic Pbcm	Monoclinic P21/c	Monoclinic I2/a
a /Å	9.1994(7)	10.4683(4)	10.0160(18)	3.9426(2)	8.5432(5)	20.26940(10)
b /Å	10.5755(7)	15.9435(7)	13.3590(8)	15.4386(10)	13.6445(7)	13.45190(10)
c /Å	12.5406(9)	16.1439(7)	18.607(3)	16.9710(11)	14.2258(12)	34.6461(2)
α /°	96.044(6)	115.451(4)	90	90	90	90
β /°	90.236(6)	99.666(4)	137.88(3)	90	106.767(8)	93.0110(10)
γ /°	111.487(7)	98.366(4)	90	90	90	90
Vol /Å^3	1127.75(15)	2325.60(19)	1669.7(9)	1033.00(11)	1587.8(2)	9433.63(10)
Z, calc density (g.cm^{-3})	2, 1.435	6, 1.495	4, 1.428	4, 2.070	4, 1.1635	16, 1.329
Abs coeff (mm^{-1})	1.844	2.649	2.465	7.802	0.073	2.971
F(000)	496.0	1056.0	736.0	617.0	592.0	3872.0
Crystal, colour	Prism, yellow	Prism, yellow	Plate, colourless	Plate, green	Block, brown	Block, yellow
Crystal dimensions/mm^3	0.3 x0.2 x 0.2	0.5 x 0.5 x 0.4	0.6 × 0.6 × 0.2	0.4 x 0.2 x 0.05	0.3 x 0.2 x 0.1	0.1 x 0.1 x 0.07
θ range(deg)	7.342 to 58.486	6.99 to 58.312	6.79 to 58.734	7.136 to 58.196	6.68 to 58.48	5.108 to 142.094
Reflections collected/unique	8902	19054	8651	4356	8008	28952
Rint	0.0432	0.0332	0.0683	0.0428	0.0339	0.0613
Data/restraint/ parameters	5126/0/299	10510/0/60 1	3894/0/203	1255/36/71	3706/0/193	8942/0/564
Final R indices [$F^2 < 2θ(F^2)$]	0.0515, 0.0916	0.0394, 0.0848	0.0523, 0.1198	0.0343, 0.0610	0.0652, 0.1598	0.0562, 0.1579
R indices (all data): R1, wR2	0.0693, 0.1005	0.0601, 0.0848	0.1016, 0.1450	0.0573, 0.0683	0.1417, 0.2175	0.0592, 0.1608
Largest diff peak and hole/ e Å^{-3}	0.45/-0.47	0.40/-0.54	0.68/-0.69	0.74/-0.56	0.32/-0.38	1.46/-1.51

Electrochemical Properties

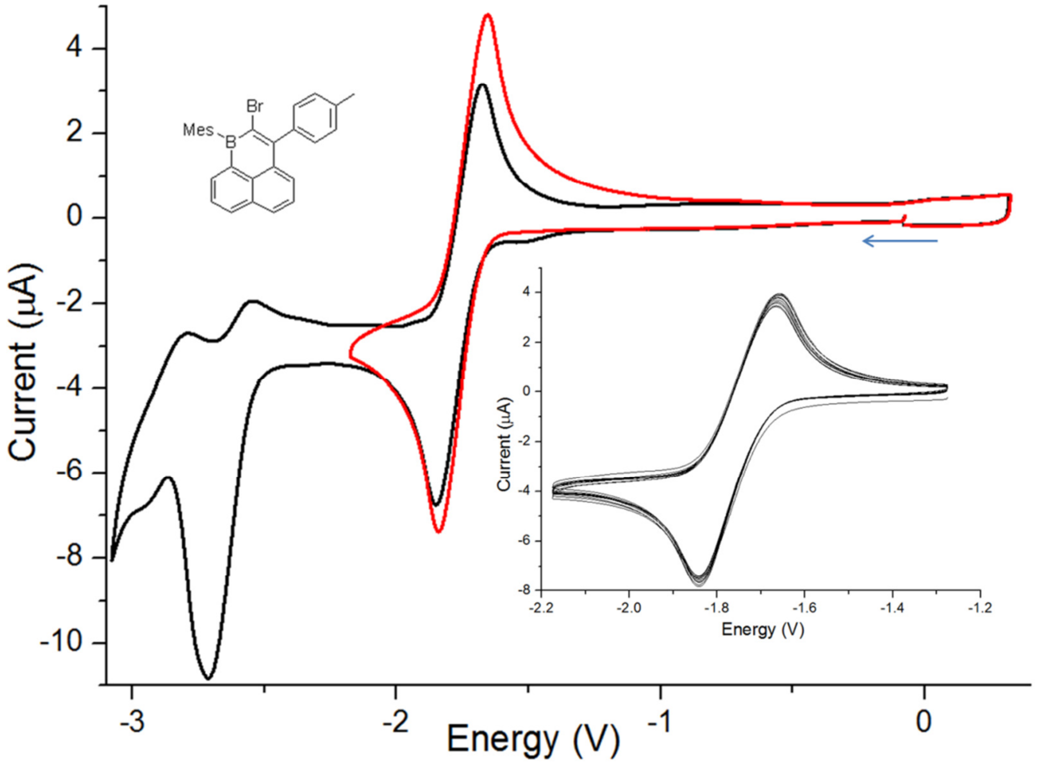

Figure S2: Cyclic voltammogram of **12** (1 mM) in THF with [$^{n}Bu_4N$][PF_6] (0.1 M) as the supporting electrolyte at a scan rate of 50 mV/s. Insert = 10 cycles of first reduction wave.

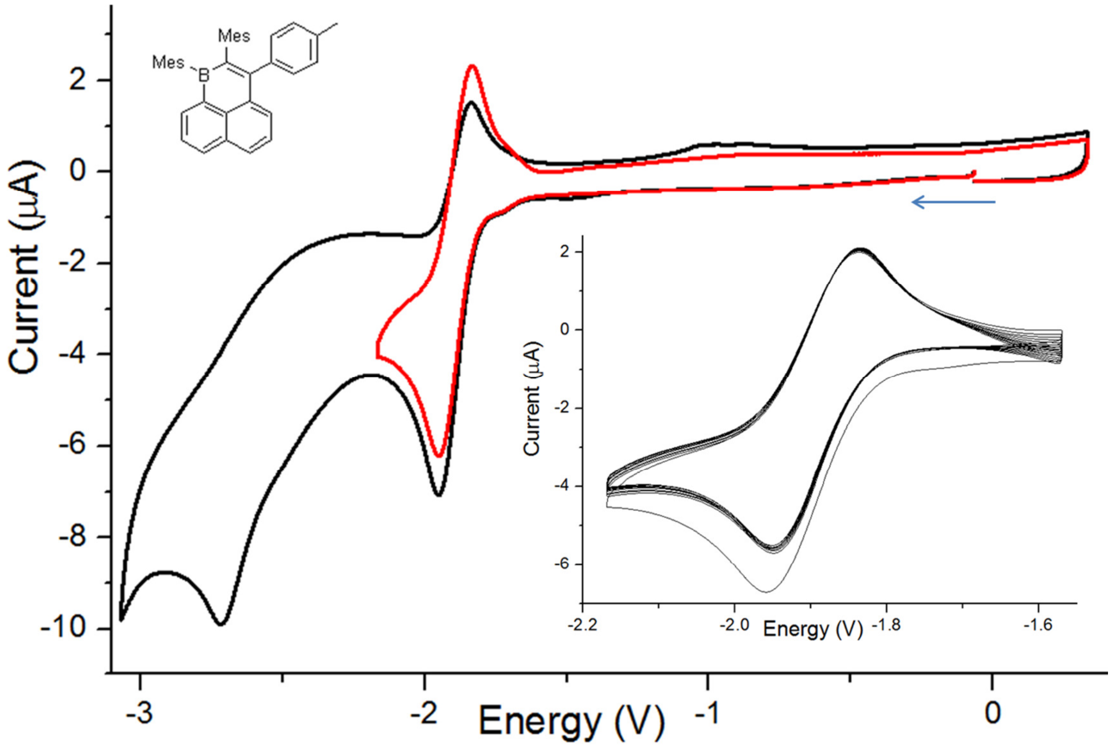

Figure S3: Cyclic voltammogram of **13** (1 mM) in THF with [$^{n}Bu_4N$][PF_6] (0.1 M) as the supporting electrolyte at a scan rate of 50 mV/s. Insert = 10 cycles of first reduction wave.

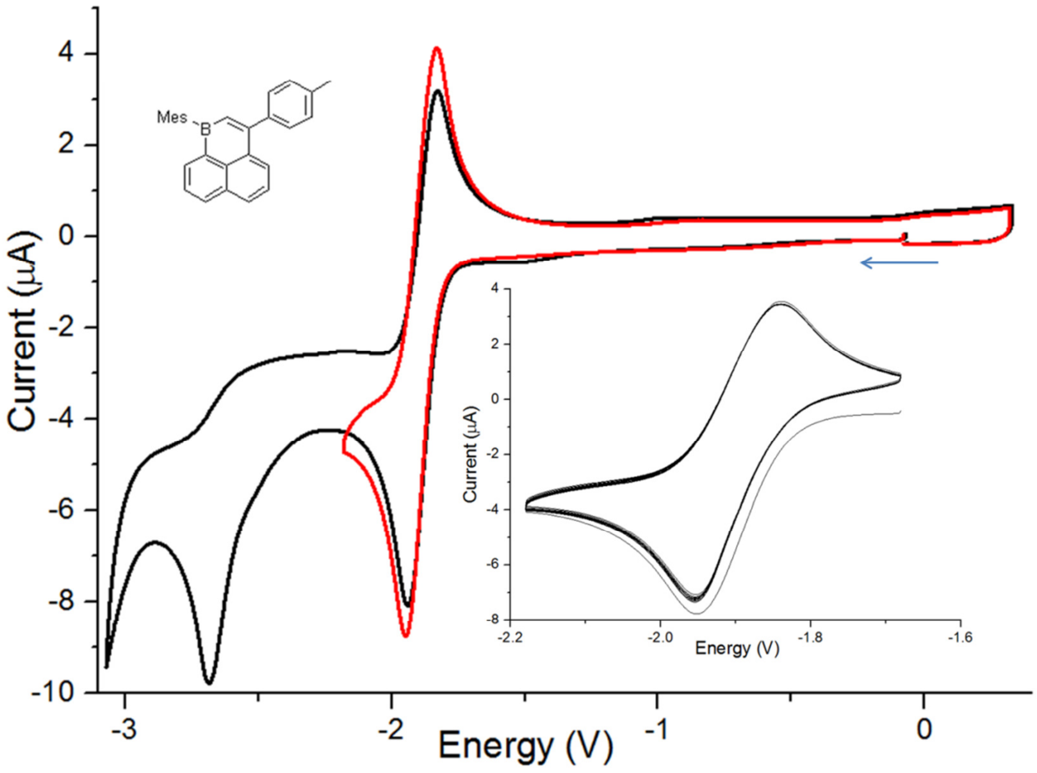

Figure S4: Cyclic voltammogram of **14** (1 mM) in THF with [^{n}Bu$_4$N][PF$_6$] (0.1 M) as the supporting electrolyte at a scan rate of 50 mV/s. Insert = 10 cycles of first reduction wave.

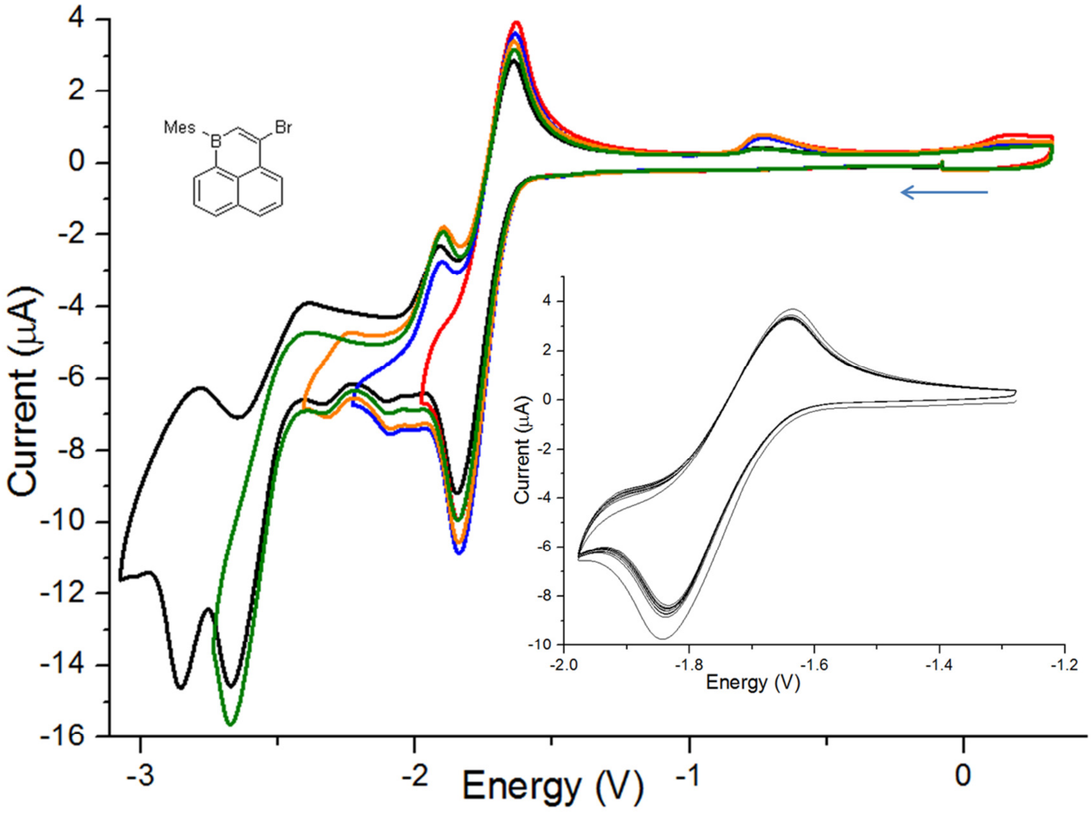

Figure S5: Cyclic voltammogram of **15** (1 mM) in THF with [^{n}Bu$_4$N][PF$_6$] (0.1 M) as the supporting electrolyte at a scan rate of 50 mV/s. Insert = 10 cycles of first reduction wave.

Table S2: Summary of cyclic voltammetry results

**Compound**	**First Reduction**			**Second Reduction**
	**E_{ap} (V)**	**$E_{1/2}$ (V)**	**E_{LUMO} (eV)**	**E_{ap} (V)**
**13**	-1.95	-1.89	-2.97	-2.72
**14**	-1.95	-1.89	-2.98	-2.68
**12**	-1.84	-1.75	-3.11	-2.71
**15**	-1.85	-1.74	-3.13	-2.11

Measured in THF (1 mM) with $[^nBu_4N][PF_6]$ (0.1 M) as the supporting electrolyte at a scan rate of 50 mV/s. Potentials are given relative to the Fc/Fc^+ redox couple. E_{LUMO} =−4.8 eV – (onset of first reduction) as Fc/Fc^+ is taken to be −4.8 eV vs vacuum level.[1] No oxidation waves are observed within the potential window on THF. The first reduction wave of each compound was reversible to at least 10 cycles.

DFT Calculations

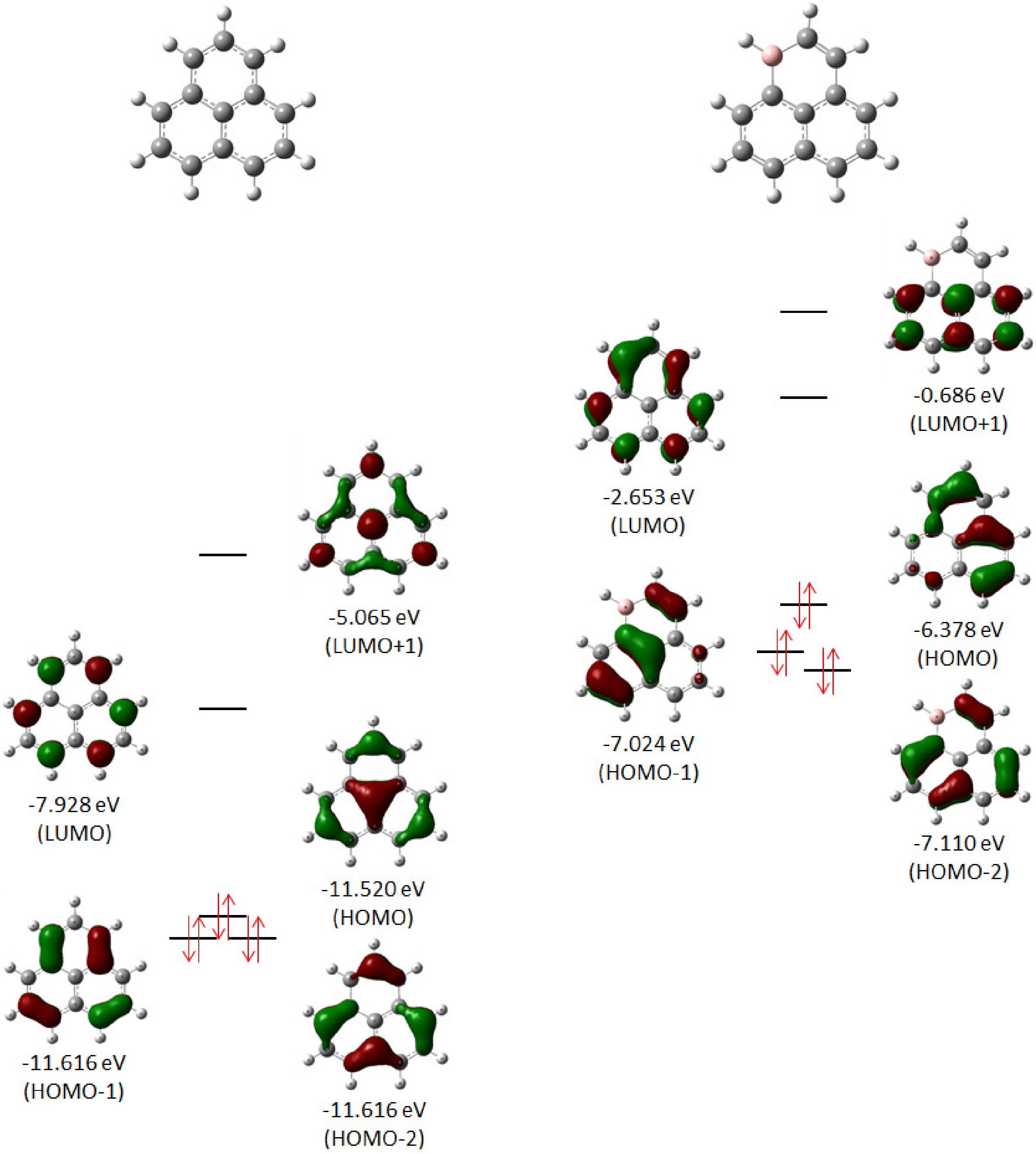

Figure S6: Molecular orbital energy levels and molecular orbital contours (isovalue = 0.05) of the LUMO+1 to HOMO-2 of **1**$^+$ (left) and the unsubstituted 1-boraphenalene analogue (right)

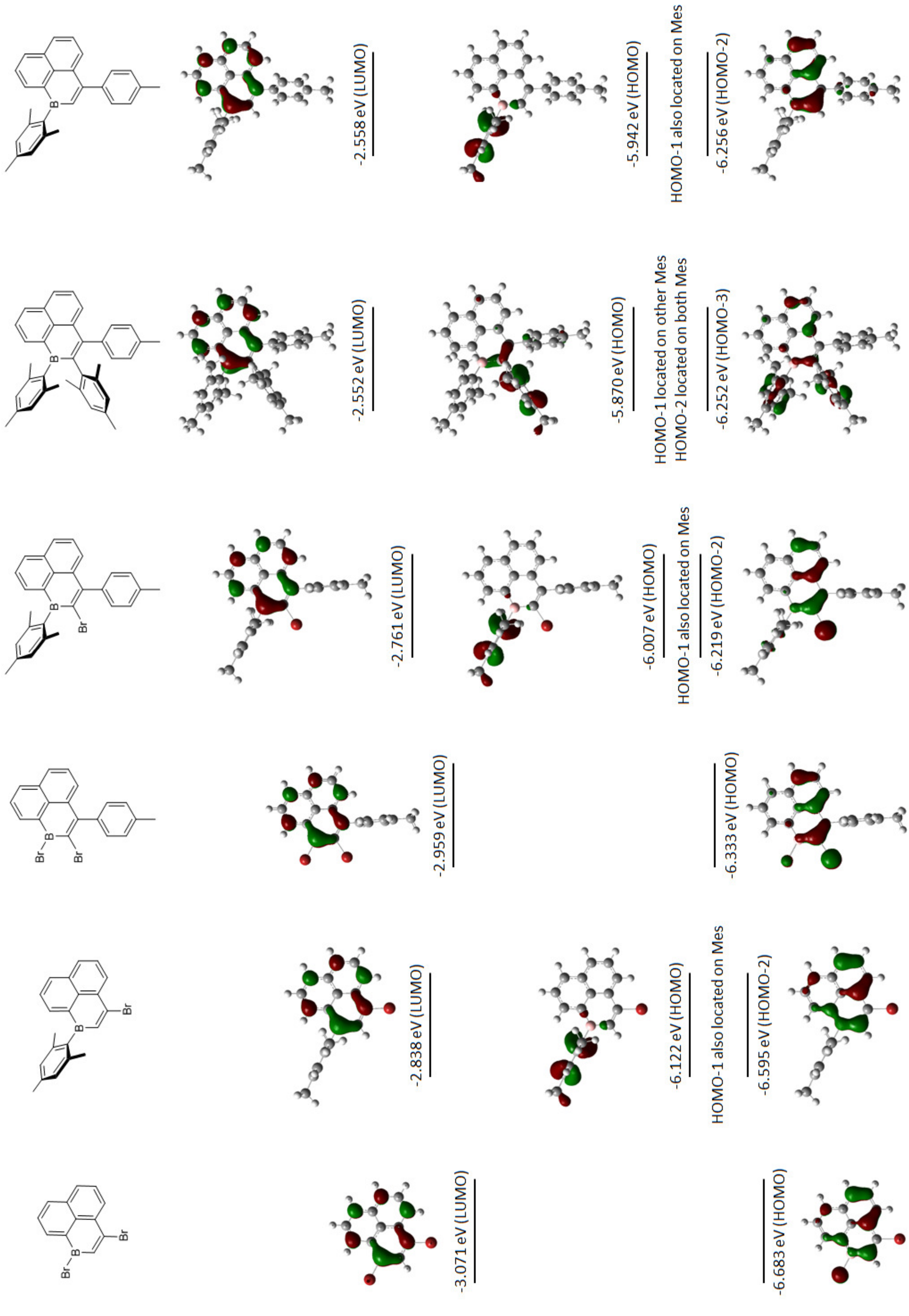

Figure S7: Molecular orbital energy levels and molecular orbital contours (isovalue = 0.05) of the LUMO, HOMO and HOMO-2 of (from left to right) **9**, **15**, **5c**, **12**, **13** and **14**.

Optimised Structure Coordinates

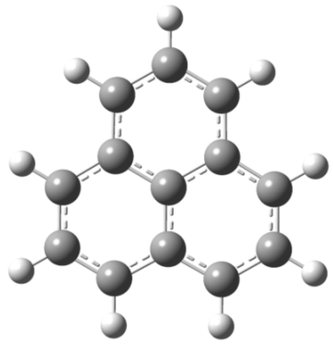

1$^+$ E(RB3LYP) = -500.694211607

C	-2.244385000	-3.001532000	-0.000519000
C	-2.979503000	-1.792559000	-0.000417000
C	-4.394281000	-1.773754000	-0.000468000
C	-0.853504000	-2.995735000	-0.000465000
C	-2.275328000	-0.559661000	-0.000259000
C	-0.855557000	-0.553032000	-0.000206000
C	-0.164327000	-1.787606000	-0.000310000
C	-0.876424000	1.889609000	0.000069000
C	-2.267292000	1.882413000	0.000016000
C	-2.991020000	0.666584000	-0.000151000
C	-4.405546000	0.634565000	-0.000206000
C	-5.096008000	-0.572851000	-0.000364000
H	-6.178469000	-0.577900000	-0.000403000
H	-4.954697000	1.569914000	-0.000121000
H	-0.307922000	-3.930672000	-0.000547000
H	0.920291000	-1.785105000	-0.000273000
H	-0.339585000	2.829592000	0.000202000
H	-2.779799000	-3.944797000	-0.000641000
H	-4.934680000	-2.714184000	-0.000590000
H	-2.811550000	2.820615000	0.000112000
C	-0.175933000	0.687984000	-0.000062000
H	0.908652000	0.695709000	-0.000060000

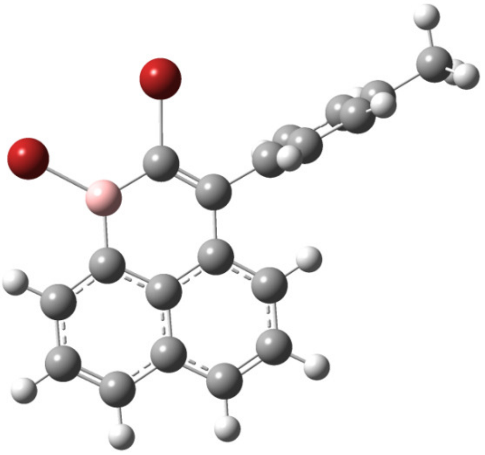

5c E(RB3LYP) = -5905.24748137

Br	1.773381000	-1.430468000	2.508664000
C	6.028010000	-0.753048000	1.558829000
C	7.181924000	-1.405264000	1.125920000
H	7.133632000	-2.467433000	0.916734000
C	8.398855000	-0.728638000	0.954066000
H	9.275234000	-1.269097000	0.615645000
C	8.471672000	0.621023000	1.216907000
H	9.406863000	1.156187000	1.087728000
C	7.334639000	1.338953000	1.659471000
C	7.413750000	2.729043000	1.928857000
H	8.361719000	3.238108000	1.790673000
C	6.304257000	3.414895000	2.357507000
H	6.359800000	4.477163000	2.564478000
C	5.082650000	2.741107000	2.531935000
H	4.222546000	3.302517000	2.870766000
C	4.948142000	1.377269000	2.282319000
C	6.097077000	0.647090000	1.833756000
C	3.646172000	0.720969000	2.479957000
C	3.498689000	-0.616228000	2.237047000
C	2.507356000	1.569557000	2.952657000
C	1.672585000	2.217040000	2.038396000
H	1.845734000	2.094726000	0.975053000
C	0.619765000	3.011678000	2.482364000
H	-0.016780000	3.504658000	1.754469000
C	0.367090000	3.183992000	3.846518000
C	-0.797586000	4.017428000	4.323715000
H	-1.049420000	4.799806000	3.604263000
H	-1.690986000	3.398535000	4.460901000
H	-0.581261000	4.494462000	5.282514000
C	1.210794000	2.537082000	4.754718000
H	1.040510000	2.655292000	5.820156000
C	2.265456000	1.741145000	4.318236000
H	2.903202000	1.245487000	5.041603000
B	4.678517000	-1.465985000	1.756296000
Br	4.502272000	-3.373158000	1.403762000

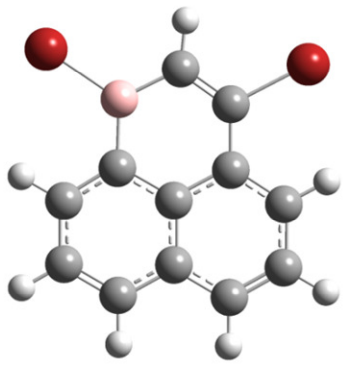

9 E(RB3LYP) = -5634.81856824

C	-2.272373000	-3.032267000	-0.000525000

C	-2.981723000	-1.805584000	-0.000416000
C	-4.399009000	-1.790279000	-0.000463000
C	-0.896538000	-3.056034000	-0.000481000
C	-2.252314000	-0.577348000	-0.000255000
C	-0.821680000	-0.610644000	-0.000208000
C	-0.181932000	-1.847646000	-0.000322000
C	-0.893449000	2.019057000	0.000077000
C	-2.242768000	1.919502000	0.000021000
C	-2.986221000	0.659796000	-0.000144000
C	-4.374819000	0.616185000	-0.000204000
C	-5.080960000	-0.598428000	-0.000363000
H	-6.164250000	-0.583306000	-0.000406000
H	-4.930159000	1.543985000	-0.000126000
H	-0.364636000	-4.000273000	-0.000568000
H	0.901848000	-1.873324000	-0.000290000
H	-0.437032000	3.001097000	0.000201000
H	-2.837917000	-3.958366000	-0.000647000
H	-4.933862000	-2.734029000	-0.000589000
B	-0.079308000	0.735277000	-0.000036000
Br	1.872972000	0.848994000	0.000048000
Br	-3.253153000	3.571907000	0.000157000

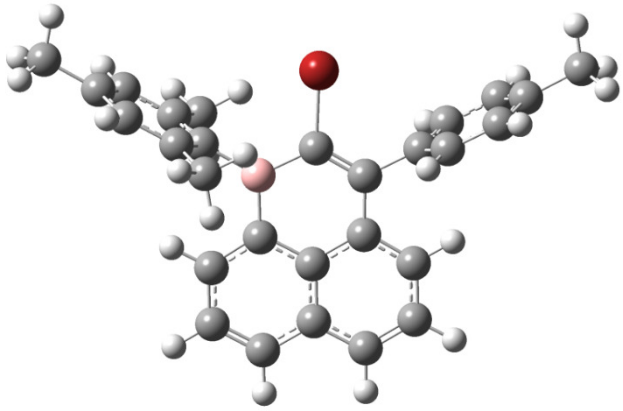

12 E(RB3LYP) = -3680.75090111

C	-2.220487000	-2.960866000	-0.009991000
C	-2.939531000	-1.739912000	-0.012413000
C	-4.357357000	-1.729355000	-0.020880000
C	-0.843912000	-2.972867000	-0.003412000
C	-2.217172000	-0.508011000	-0.007006000
C	-0.790325000	-0.524085000	-0.000985000
C	-0.138994000	-1.757295000	0.000553000
C	-0.884212000	2.068957000	0.003179000
C	-2.248527000	2.025342000	-0.001473000
C	-2.943750000	0.727173000	-0.008743000
C	-4.335536000	0.680035000	-0.018712000
C	-5.039730000	-0.537391000	-0.024553000
H	-6.123407000	-0.524389000	-0.031957000
H	-4.895609000	1.605574000	-0.022143000

H	-0.304693000	-3.913316000	-0.001737000
H	0.945752000	-1.772712000	0.004792000
H	-2.777816000	-3.892252000	-0.013742000
H	-4.891119000	-2.673932000	-0.025015000
B	0.002638000	0.808245000	0.002662000
C	1.577408000	0.835060000	0.005103000
C	2.297650000	0.838082000	-1.207473000
C	2.295110000	0.835082000	1.219038000
C	3.693650000	0.835712000	-1.187832000
C	3.691262000	0.832747000	1.202166000
C	4.411846000	0.834574000	0.007940000
H	4.233409000	0.836273000	-2.131042000
H	4.229131000	0.830956000	2.146437000
C	1.569553000	0.858335000	-2.534027000
H	0.895126000	0.002483000	-2.639766000
H	0.962141000	1.763695000	-2.636163000
H	2.270692000	0.834223000	-3.370848000
C	1.564336000	0.852220000	2.544139000
H	0.957561000	1.757861000	2.647496000
H	0.888943000	-0.003332000	2.646075000
H	2.263732000	0.825139000	3.382326000
C	5.921743000	0.867584000	0.009236000
H	6.292865000	1.898588000	0.000468000
H	6.331072000	0.381764000	0.898408000
H	6.333001000	0.366305000	-0.870492000
C	-3.089458000	3.263322000	0.000632000
C	-3.523227000	3.829598000	1.201942000
C	-3.467725000	3.871464000	-1.199261000
C	-4.308877000	4.978607000	1.200205000
H	-3.240186000	3.370771000	2.142726000
C	-4.253907000	5.019594000	-1.193984000
H	-3.141163000	3.444997000	-2.141146000
C	-4.685881000	5.596531000	0.004277000
H	-4.632578000	5.402073000	2.145780000
H	-4.534701000	5.475279000	-2.138330000
C	-5.507263000	6.863129000	0.006303000
H	-4.861544000	7.748102000	0.000531000
H	-6.150521000	6.923390000	-0.874800000
H	-6.140654000	6.926730000	0.894041000
Br	-0.003794000	3.796380000	0.015214000

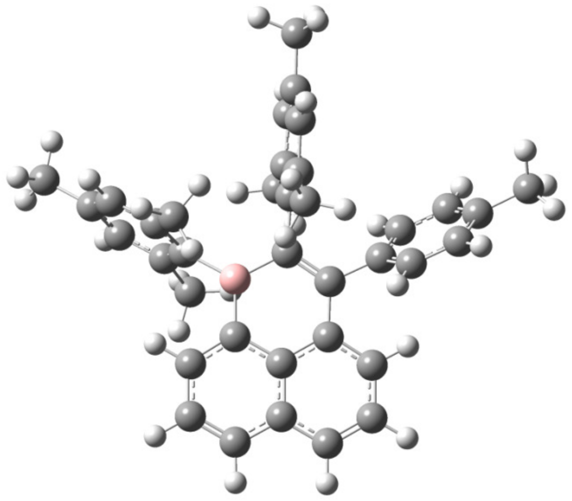

13 E(RB3LYP) = -1456.28103287

C	-2.170765000	-2.911544000	-0.424462000
C	-2.892128000	-1.714042000	-0.194289000
C	-4.297714000	-1.731100000	-0.010893000
C	-0.800795000	-2.897024000	-0.557838000
C	-2.181225000	-0.476548000	-0.129621000
C	-0.759220000	-0.470563000	-0.224637000
C	-0.104067000	-1.681889000	-0.438937000
C	-0.848023000	2.161441000	-0.045482000
C	-2.218709000	2.051276000	0.029260000
C	-2.906138000	0.744523000	0.060694000
C	-4.281222000	0.663682000	0.265846000
C	-4.973281000	-0.561391000	0.234900000
H	-6.044716000	-0.569630000	0.398949000
H	-4.844341000	1.567860000	0.451222000
H	-0.258593000	-3.818961000	-0.735676000
H	0.978451000	-1.683530000	-0.509284000
H	-2.719811000	-3.845751000	-0.488205000
H	-4.824198000	-2.678968000	-0.052536000
B	0.014437000	0.864590000	-0.051202000
C	1.585976000	0.819807000	0.162237000
C	2.508508000	1.117644000	-0.864047000
C	2.098083000	0.404443000	1.414141000
C	3.881723000	1.038578000	-0.620105000
C	3.477589000	0.356531000	1.629382000
C	4.390746000	0.677984000	0.626242000
H	4.571334000	1.265096000	-1.429106000
H	3.846988000	0.048900000	2.604262000
C	2.046178000	1.514021000	-2.248983000
H	1.134053000	0.987420000	-2.541216000
H	1.838320000	2.586239000	-2.301385000
H	2.811696000	1.288026000	-2.995307000
C	1.182489000	-0.041485000	2.537625000
H	0.250187000	0.525497000	2.569160000
H	0.908316000	-1.095720000	2.424337000

H	1.672212000	0.067495000	3.508416000
C	5.878220000	0.651095000	0.884222000
H	6.240353000	1.635427000	1.201783000
H	6.134554000	-0.060662000	1.672800000
H	6.434509000	0.375311000	-0.015241000
C	-3.094729000	3.269218000	0.065483000
C	-3.061863000	4.156476000	1.146285000
C	-3.988414000	3.546952000	-0.977866000
C	-3.882827000	5.279375000	1.177937000
H	-2.396581000	3.961326000	1.977771000
C	-4.798815000	4.677832000	-0.948532000
H	-4.049120000	2.869807000	-1.822568000
C	-4.759839000	5.567947000	0.128808000
H	-3.837180000	5.944584000	2.034490000
H	-5.474990000	4.868170000	-1.776286000
C	-5.616141000	6.810738000	0.146267000
H	-5.091719000	7.653925000	-0.316782000
H	-6.547208000	6.662727000	-0.405877000
H	-5.870050000	7.105930000	1.167030000
C	-0.203900000	3.518240000	-0.179751000
C	0.429816000	4.142191000	0.916265000
C	-0.200380000	4.176835000	-1.429358000
C	1.034319000	5.389219000	0.748312000
C	0.430330000	5.418006000	-1.554843000
C	1.055294000	6.045287000	-0.480705000
H	1.501887000	5.860354000	1.608762000
H	0.424463000	5.907992000	-2.524713000
C	-0.877482000	3.604143000	-2.655034000
H	-1.917921000	3.937480000	-2.714365000
H	-0.374407000	3.942758000	-3.563988000
H	-0.886933000	2.514315000	-2.656641000
C	0.476109000	3.511116000	2.288663000
H	1.362923000	2.880458000	2.397335000
H	0.521888000	4.279889000	3.064049000
H	-0.393659000	2.882391000	2.485061000
C	1.750298000	7.375919000	-0.644160000
H	1.651115000	7.990194000	0.254559000
H	2.821934000	7.242363000	-0.829896000
H	1.339995000	7.939261000	-1.485562000

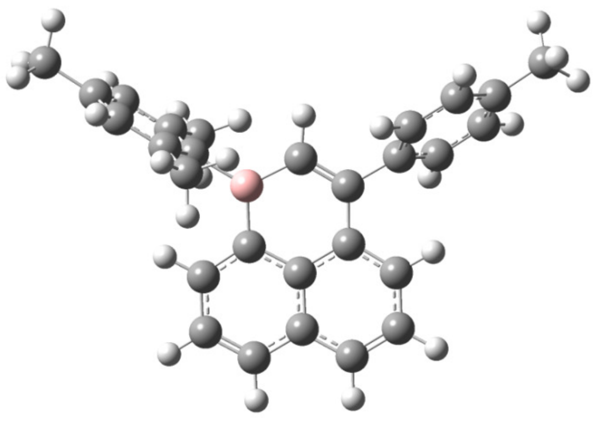

14 E(RB3LYP) = -1107.20940616

C	-2.246121000	-3.052043000	0.186963000
C	-2.973654000	-1.838199000	0.104241000
C	-4.390680000	-1.843375000	0.051990000
C	-0.870304000	-3.052915000	0.209895000
C	-2.261958000	-0.600171000	0.060165000
C	-0.832321000	-0.606935000	0.068531000
C	-0.174646000	-1.832715000	0.143302000
C	-0.939974000	1.992057000	-0.093741000
C	-2.303537000	1.924177000	-0.045092000
C	-3.002860000	0.627948000	-0.011269000
C	-4.390400000	0.561134000	-0.089080000
C	-5.083650000	-0.662713000	-0.054497000
H	-6.165935000	-0.663743000	-0.113625000
H	-4.959329000	1.476193000	-0.185317000
H	-0.324421000	-3.987673000	0.271507000
H	0.910672000	-1.840350000	0.146257000
H	-0.501403000	2.987146000	-0.114270000
H	-2.797258000	-3.986307000	0.227628000
H	-4.915107000	-2.792744000	0.086021000
B	-0.063006000	0.737424000	-0.033568000
C	1.515183000	0.816285000	-0.052017000
C	2.222130000	0.807436000	-1.273273000
C	2.249206000	0.917888000	1.148609000
C	3.615907000	0.896295000	-1.275537000
C	3.642847000	1.005870000	1.111134000
C	4.347661000	0.998500000	-0.092352000
H	4.143119000	0.886066000	-2.225959000
H	4.191105000	1.083562000	2.046340000
C	1.480862000	0.696188000	-2.588840000
H	0.946651000	-0.256249000	-2.670024000
H	0.733385000	1.488958000	-2.695541000
H	2.165524000	0.766485000	-3.436778000
C	1.538496000	0.932495000	2.485592000
H	0.798958000	1.738024000	2.537029000
H	1.000766000	-0.004180000	2.665461000
H	2.243639000	1.074346000	3.307351000
C	5.852220000	1.129353000	-0.115772000

H	6.156764000	2.179290000	-0.193078000
H	6.301934000	0.726283000	0.794984000
H	6.285646000	0.601620000	-0.969089000
C	-3.094641000	3.190311000	-0.020250000
C	-3.936387000	3.511961000	1.053652000
C	-2.965376000	4.126229000	-1.053355000
C	-4.616666000	4.724664000	1.089946000
H	-4.044927000	2.813281000	1.875782000
C	-3.658269000	5.332638000	-1.017677000
H	-2.325717000	3.897335000	-1.898257000
C	-4.493309000	5.657480000	0.054903000
H	-5.253161000	4.950767000	1.939821000
H	-3.547724000	6.032759000	-1.839795000
C	-5.214583000	6.982272000	0.110288000
H	-4.623566000	7.728715000	0.652466000
H	-5.400145000	7.377233000	-0.891064000
H	-6.175360000	6.893020000	0.622940000

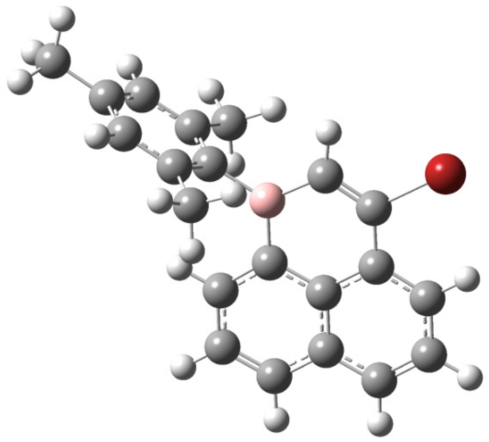

15 E(RB3LYP) = -3410.31947870

C	-2.270161000	-3.023797000	-0.000450000
C	-2.986184000	-1.799790000	-0.000284000
C	-4.403197000	-1.788167000	-0.000193000
C	-0.894616000	-3.040335000	-0.000525000
C	-2.260768000	-0.569520000	-0.000204000
C	-0.831849000	-0.590530000	-0.000280000
C	-0.186738000	-1.825496000	-0.000435000
C	-0.900381000	2.021140000	-0.000023000
C	-2.248105000	1.922724000	0.000043000
C	-2.996385000	0.665911000	-0.000037000
C	-4.384726000	0.619886000	0.000051000
C	-5.087422000	-0.597119000	-0.000026000
H	-6.170882000	-0.584754000	0.000048000
H	-4.942354000	1.546390000	0.000183000
H	-0.358232000	-3.982361000	-0.000651000
H	0.898330000	-1.843958000	-0.000486000
H	-0.455488000	3.010123000	0.000061000
H	-2.831231000	-3.952838000	-0.000515000

H	-4.936845000	-2.732794000	-0.000255000
B	-0.042927000	0.743946000	-0.000188000
Br	-3.265525000	3.579062000	0.000275000
C	1.533357000	0.813104000	-0.000158000
C	2.252720000	0.854487000	-1.213400000
C	2.252581000	0.854644000	1.213017000
C	3.646966000	0.931609000	-1.195139000
C	3.646946000	0.931801000	1.194802000
C	4.365311000	0.973090000	-0.000095000
H	4.185224000	0.961118000	-2.138754000
H	4.185157000	0.961483000	2.138420000
C	1.525486000	0.814179000	-2.540777000
H	0.965964000	-0.118751000	-2.665187000
H	0.803039000	1.632304000	-2.627481000
H	2.222787000	0.897129000	-3.376947000
C	1.525381000	0.814486000	2.540421000
H	0.802756000	1.632469000	2.626971000
H	0.966076000	-0.118544000	2.665068000
H	2.222685000	0.897778000	3.376555000
C	5.870968000	1.091191000	-0.000317000
H	6.184485000	2.141160000	-0.013376000
H	6.307294000	0.632212000	0.890202000
H	6.308896000	0.610054000	-0.878359000

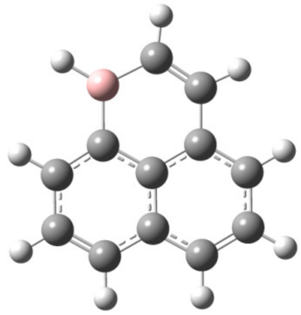

$C_{12}BH_9$ E(RB3LYP) = -487.680882313

C	-2.269383000	-3.037923000	-0.000525000
C	-2.985745000	-1.814233000	-0.000419000
C	-4.404411000	-1.783430000	-0.000467000
C	-0.891975000	-3.051115000	-0.000477000
C	-2.254163000	-0.590911000	-0.000261000
C	-0.825324000	-0.602219000	-0.000212000
C	-0.180098000	-1.837123000	-0.000321000
C	-0.900470000	2.014315000	0.000077000
C	-2.255925000	1.914503000	0.000017000
C	-2.969220000	0.647996000	-0.000152000
C	-4.358862000	0.626190000	-0.000204000
C	-5.077623000	-0.583197000	-0.000361000
H	-6.161264000	-0.563736000	-0.000398000
H	-4.900489000	1.566830000	-0.000119000
H	-0.356628000	-3.993929000	-0.000560000
H	0.905063000	-1.860062000	-0.000287000

H	-0.468396000	3.011774000	0.000209000
H	-2.826408000	-3.969585000	-0.000646000
H	-4.951063000	-2.720783000	-0.000589000
B	-0.055795000	0.737448000	-0.000047000
H	1.139183000	0.747206000	0.000013000
H	-2.882363000	2.805620000	0.000098000

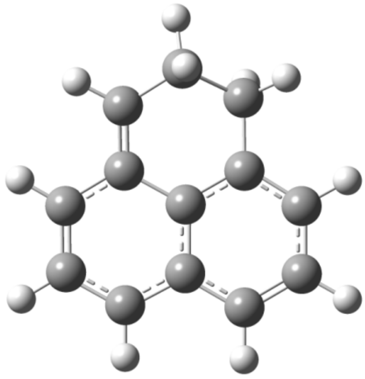

E(RB3LYP) = -501.880342524

C	-2.238357000	-2.970053000	0.001997000
C	-3.000338000	-1.783432000	-0.035862000
C	-4.416327000	-1.804505000	-0.093410000
C	-0.840060000	-2.971194000	0.069216000
C	-2.317207000	-0.530895000	0.012775000
C	-0.862524000	-0.533478000	0.022772000
C	-0.157072000	-1.772269000	0.073628000
C	-0.874371000	1.939421000	-0.359206000
C	-2.292664000	1.973233000	0.231926000
C	-3.040405000	0.670834000	0.044247000
C	-4.428947000	0.608795000	-0.018675000
C	-5.114068000	-0.617434000	-0.094642000
H	-6.195819000	-0.620672000	-0.143708000
H	-5.001135000	1.530184000	-0.000730000
H	-0.302690000	-3.910170000	0.100973000
H	0.927068000	-1.757290000	0.092528000
H	-0.264954000	2.764681000	0.019551000
H	-2.763494000	-3.919963000	-0.016231000
H	-4.935099000	-2.754905000	-0.134193000
H	-2.220660000	2.170819000	1.308549000
C	-0.180906000	0.648122000	-0.149396000
H	-2.853437000	2.804610000	-0.197590000
H	-0.896390000	2.089313000	-1.454101000
H	0.904229000	0.625373000	-0.210606000

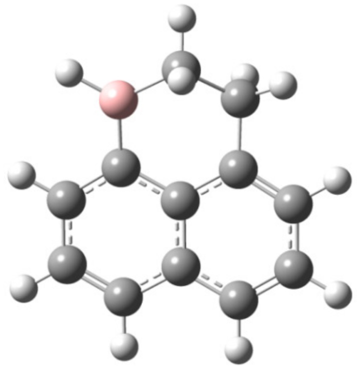

E(RB3LYP) = -488.890979900

C	-2.266023000	-3.022699000	-0.002517000
C	-2.994447000	-1.804992000	-0.035866000
C	-4.409365000	-1.790517000	-0.129759000
C	-0.892762000	-3.035011000	0.075985000
C	-2.274552000	-0.569957000	0.031100000
C	-0.835509000	-0.590435000	0.065438000
C	-0.184384000	-1.818281000	0.095678000
C	-0.868981000	2.041080000	-0.317525000
C	-2.287237000	1.974865000	0.278340000
C	-3.003390000	0.658698000	0.052429000
C	-4.379032000	0.621372000	-0.056013000
C	-5.085395000	-0.596582000	-0.153502000
H	-6.166541000	-0.581486000	-0.235314000
H	-4.934986000	1.553780000	-0.049725000
H	-0.356605000	-3.977054000	0.102948000
H	0.900539000	-1.834919000	0.114533000
H	-0.346668000	2.941790000	0.020468000
H	-2.818654000	-3.956433000	-0.040857000
H	-4.944206000	-2.732851000	-0.184670000
B	-0.052032000	0.725267000	-0.090406000
H	-2.206967000	2.123224000	1.364364000
H	-2.913864000	2.793706000	-0.087955000
H	-0.932355000	2.146743000	-1.415666000
H	1.143690000	0.706918000	-0.120984000

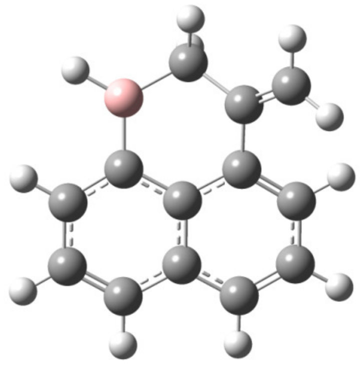

E(RB3LYP) = -526.987464982

C	-2.340157000	-2.893781000	-0.139505000
C	-3.070462000	-1.686750000	0.019260000
C	-4.481485000	-1.690500000	0.152352000
C	-0.970228000	-2.893286000	-0.259027000
C	-2.357282000	-0.446566000	0.041177000
C	-0.921638000	-0.458552000	-0.046219000
C	-0.267288000	-1.674898000	-0.200272000
C	-0.964822000	2.125834000	0.549191000
C	-2.388114000	2.083565000	0.044945000
C	-3.094097000	0.779216000	0.141727000
C	-4.469650000	0.718148000	0.297303000
C	-5.162754000	-0.507809000	0.303497000
H	-6.239977000	-0.509392000	0.426643000
H	-5.026178000	1.638890000	0.425739000
H	-0.432603000	-3.826647000	-0.382252000
H	0.816234000	-1.682216000	-0.259295000
H	-0.969771000	2.093136000	1.656892000
H	-2.889906000	-3.829512000	-0.165623000
H	-5.009712000	-2.638027000	0.141643000
B	-0.142425000	0.847743000	0.169546000
H	1.052217000	0.855416000	0.141179000
C	-2.957715000	3.146362000	-0.537417000
H	-0.478088000	3.069380000	0.289872000
H	-2.431647000	4.091844000	-0.609521000
H	-3.946546000	3.101802000	-0.978269000

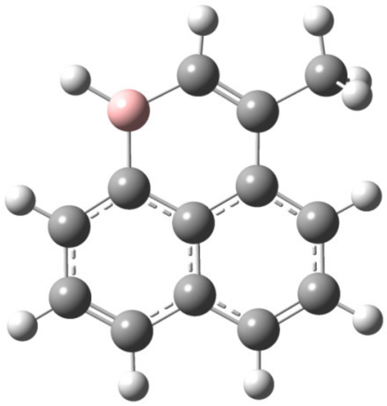

E(RB3LYP) = -527.007160797

C	-2.262513000	-3.044741000	-0.000534000
C	-2.984224000	-1.823020000	-0.000417000
C	-4.401597000	-1.812753000	-0.000468000
C	-0.886639000	-3.056451000	-0.000487000
C	-2.263424000	-0.591279000	-0.000248000
C	-0.835068000	-0.608124000	-0.000198000
C	-0.182814000	-1.837913000	-0.000321000
C	-0.917534000	1.997928000	0.000081000
C	-2.281124000	1.940746000	0.000027000
C	-2.988094000	0.648979000	-0.000136000
C	-4.378269000	0.596800000	-0.000196000
C	-5.083958000	-0.620448000	-0.000359000
H	-6.167837000	-0.608487000	-0.000400000
H	-4.949968000	1.515944000	-0.000117000
H	-0.347708000	-3.997198000	-0.000578000
H	0.902497000	-1.852150000	-0.000281000
H	-0.470327000	2.989833000	0.000194000
H	-2.819324000	-3.976539000	-0.000663000
H	-4.936774000	-2.756678000	-0.000597000
B	-0.071605000	0.730401000	0.000003000
H	1.123708000	0.746886000	-0.000015000
C	-3.098099000	3.212341000	0.000127000
H	-3.744824000	3.277564000	-0.881140000
H	-3.744903000	3.277375000	0.881349000
H	-2.440231000	4.081763000	0.000249000

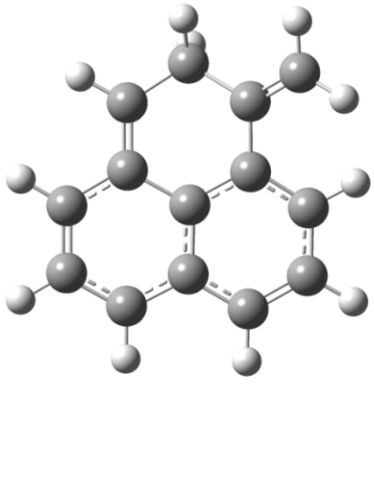

E(RB3LYP) = -539.973825845

C	-2.338947000	-2.795467000	-0.121799000
C	-3.096008000	-1.613381000	0.021049000
C	-4.508679000	-1.643654000	0.121179000
C	-0.943990000	-2.794516000	-0.230853000
C	-2.412867000	-0.360056000	0.044087000
C	-0.960928000	-0.365532000	-0.021972000
C	-0.259743000	-1.597840000	-0.175827000
C	-0.948500000	2.088725000	0.451645000
C	-2.402998000	2.127129000	0.024683000
C	-3.138181000	0.845369000	0.118685000
C	-4.528208000	0.767040000	0.238710000
C	-5.206378000	-0.461401000	0.239975000
H	-6.284928000	-0.472431000	0.336045000
H	-5.101005000	1.679998000	0.346607000
H	-0.410402000	-3.729419000	-0.343127000
H	0.823133000	-1.580145000	-0.230096000
H	-0.863445000	2.223121000	1.548598000
H	-2.867249000	-3.743251000	-0.152058000
H	-5.024930000	-2.596154000	0.109421000
C	-0.269678000	0.806969000	0.165063000
H	0.817056000	0.787866000	0.166941000
C	-2.947110000	3.249058000	-0.452322000
H	-0.381496000	2.924214000	0.031234000
H	-2.371593000	4.163034000	-0.540616000
H	-3.977622000	3.295722000	-0.782325000

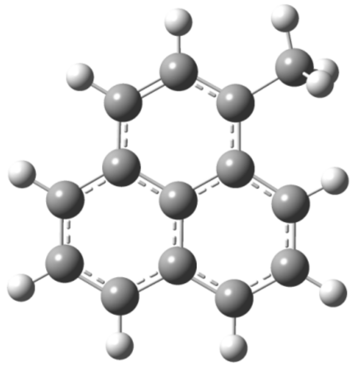

E(RB3LYP) = -540.026152902

C	-2.239492000	-3.006442000	-0.000569000
C	-2.979795000	-1.798490000	-0.000456000
C	-4.392683000	-1.796108000	-0.000570000
C	-0.851602000	-2.999394000	-0.000478000
C	-2.284135000	-0.558796000	-0.000239000
C	-0.864660000	-0.558488000	-0.000154000
C	-0.168464000	-1.786131000	-0.000275000
C	-0.889837000	1.879463000	0.000176000
C	-2.290009000	1.910676000	0.000100000

C	-3.005674000	0.668503000	-0.000118000
C	-4.417554000	0.611643000	-0.000242000
C	-5.099777000	-0.601375000	-0.000473000
H	-6.182287000	-0.611004000	-0.000570000
H	-4.989710000	1.530873000	-0.000160000
H	-0.302530000	-3.932217000	-0.000567000
H	0.916127000	-1.776735000	-0.000210000
H	-0.343474000	2.814633000	0.000351000
H	-2.775064000	-3.949561000	-0.000733000
H	-4.924354000	-2.741365000	-0.000735000
C	-0.189712000	0.684508000	0.000050000
H	0.894945000	0.697230000	0.000116000
C	-2.998908000	3.232931000	0.000188000
H	-3.637607000	3.339616000	-0.881926000
H	-3.638733000	3.339038000	0.881533000
H	-2.287421000	4.057510000	0.000888000

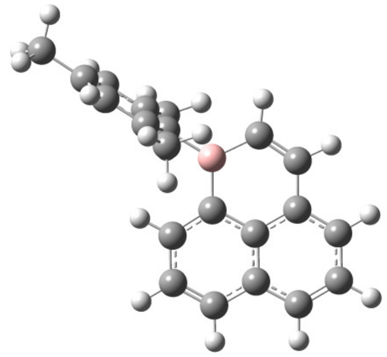

E(RB3LYP) = -836.776590311

C	-2.319700000	-3.052111000	0.002574000
C	-3.010843000	-1.814542000	0.000997000
C	-4.429050000	-1.759226000	0.000390000
C	-0.943420000	-3.090964000	0.003148000
C	-2.256221000	-0.604127000	0.000016000
C	-0.826992000	-0.642679000	0.000569000
C	-0.208053000	-1.891036000	0.002132000
C	-0.863994000	1.975028000	-0.002091000
C	-2.218970000	1.898846000	-0.002505000
C	-2.953273000	0.645082000	-0.001521000
C	-4.342459000	0.647883000	-0.002057000
C	-5.082460000	-0.548699000	-0.001117000
H	-6.165611000	-0.510846000	-0.001575000
H	-4.866892000	1.598218000	-0.003240000
H	-0.425032000	-4.043265000	0.004374000
H	0.876412000	-1.935913000	0.002572000
H	-0.412589000	2.964468000	-0.002926000
H	-2.895289000	-3.972414000	0.003337000
H	-4.990647000	-2.687685000	0.001141000
B	-0.018153000	0.688615000	-0.000573000
H	-2.828783000	2.801466000	-0.003637000

C	1.560927000	0.718312000	-0.000260000
C	2.281936000	0.743205000	1.212387000
C	2.282390000	0.740361000	-1.212591000
C	3.677781000	0.786438000	1.194894000
C	3.678313000	0.783638000	-1.194602000
C	4.397488000	0.808945000	0.000227000
H	4.216283000	0.803645000	2.138811000
H	4.217214000	0.798618000	-2.138317000
C	1.553647000	0.720142000	2.539688000
H	0.840940000	1.547333000	2.620108000
H	0.982107000	-0.204869000	2.668754000
H	2.251446000	0.799106000	3.376007000
C	1.554727000	0.714250000	-2.540180000
H	0.982629000	-0.210712000	-2.667079000
H	0.842622000	1.541710000	-2.623163000
H	2.252988000	0.790532000	-3.376365000
C	5.905714000	0.890556000	0.000630000
H	6.245200000	1.932528000	0.008466000
H	6.331548000	0.403230000	0.881278000
H	6.331215000	0.416412000	-0.887280000

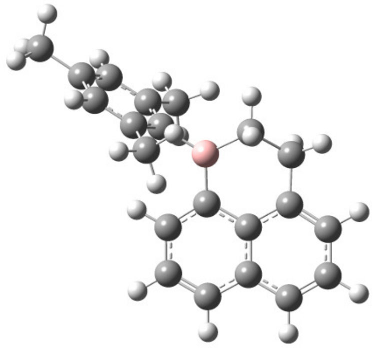

E(RB3LYP) = -837.986739243

C	-2.294604000	-3.033596000	-0.191809000
C	-3.006119000	-1.807580000	-0.133532000
C	-4.421570000	-1.772771000	-0.216433000
C	-0.922228000	-3.066209000	-0.116613000
C	-2.271365000	-0.587781000	0.019620000
C	-0.831735000	-0.625640000	0.041359000
C	-0.199052000	-1.862311000	-0.013817000
C	-0.859279000	2.026235000	-0.148277000
C	-2.262624000	1.924718000	0.471220000
C	-2.992366000	0.639372000	0.146044000
C	-4.368831000	0.623670000	0.046856000
C	-5.087733000	-0.575742000	-0.142640000
H	-6.169211000	-0.545072000	-0.215129000
H	-4.915083000	1.557748000	0.134279000
H	-0.395539000	-4.013216000	-0.156531000
H	0.885346000	-1.895896000	0.000920000
H	-0.328115000	2.905862000	0.228760000

H	-2.859219000	-3.955035000	-0.295748000
H	-4.964464000	-2.704019000	-0.339420000
B	-0.019706000	0.698782000	-0.018760000
H	-2.160768000	1.980674000	1.564186000
C	1.557002000	0.722009000	-0.033055000
C	2.262528000	0.875377000	1.180251000
C	2.292026000	0.614370000	-1.231244000
C	3.657282000	0.911179000	1.177788000
C	3.688685000	0.654160000	-1.197436000
C	4.392693000	0.803976000	-0.003640000
H	4.182931000	1.021680000	2.122596000
H	4.239707000	0.562731000	-2.129617000
C	1.513155000	0.988020000	2.491216000
H	0.825656000	1.841038000	2.488868000
H	0.914445000	0.093163000	2.690519000
H	2.198604000	1.122081000	3.330439000
C	1.587682000	0.477644000	-2.564815000
H	0.794397000	-0.274539000	-2.531367000
H	1.125375000	1.423322000	-2.869026000
H	2.286561000	0.189892000	-3.353040000
C	5.901221000	0.874288000	0.010838000
H	6.248030000	1.913709000	0.001655000
H	6.314399000	0.402337000	0.905991000
H	6.331885000	0.378486000	-0.862257000
H	-2.886941000	2.777195000	0.186425000
H	-0.951879000	2.194678000	-1.235413000

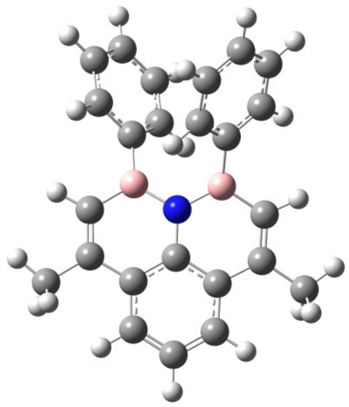

Compound 3 E(RB3LYP) = -1032.02509968

C	-0.538600000	2.487600000	-0.720300000
H	-0.729500000	3.440900000	-0.234100000
C	-0.674100000	2.424900000	-2.069300000
C	-0.332900000	1.187600000	-2.766700000
C	-0.338200000	1.150400000	-4.169400000
H	-0.607800000	2.042400000	-4.718600000
C	-0.002200000	0.004500000	-4.871900000
H	-0.003600000	0.005800000	-5.955500000
C	0.335500000	-1.142800000	-4.172800000

H	0.603900000	-2.033400000	-4.724900000
C	0.332800000	-1.183400000	-2.770200000
C	0.676100000	-2.422200000	-2.076300000
C	0.541600000	-2.488600000	-0.727400000
H	0.734600000	-3.442900000	-0.243800000
C	0.000100000	0.001100000	-2.043600000
C	1.125900000	-3.622800000	-2.874300000
H	0.348600000	-3.965300000	-3.564900000
H	1.367900000	-4.449000000	-2.205500000
H	2.013700000	-3.397400000	-3.473300000
C	-1.122400000	3.628300000	-2.864000000
H	-0.343500000	3.973400000	-3.551500000
H	-1.365800000	4.452500000	-2.192900000
H	-2.008900000	3.405700000	-3.465900000
C	0.550000000	1.481500000	1.507500000
C	1.759200000	0.874400000	1.889600000
H	2.250100000	0.186500000	1.208500000
C	2.357700000	1.147900000	3.116400000
H	3.295300000	0.670300000	3.379700000
C	1.751400000	2.032100000	4.006000000
H	2.210900000	2.241500000	4.965800000
C	0.553200000	2.650900000	3.652100000
H	0.075700000	3.341300000	4.339200000
C	-0.025600000	2.389700000	2.414300000
H	-0.953900000	2.888500000	2.153900000
C	-0.550200000	-1.488100000	1.502100000
C	0.023900000	-2.396100000	2.410100000
H	0.951900000	-2.896100000	2.151100000
C	-0.555700000	-2.654100000	3.648300000
H	-0.079100000	-3.343700000	4.336800000
C	-1.753000000	-2.033000000	4.001000000
H	-2.212700000	-2.240100000	4.961200000
C	-2.358200000	-1.149900000	3.109700000
H	-3.295400000	-0.670700000	3.371800000
C	-1.758800000	-0.879200000	1.882800000
H	-2.248200000	-0.191600000	1.200400000
B	-0.041100000	1.285200000	0.068100000
B	0.042600000	-1.288900000	0.064100000
N	0.000400000	-0.000800000	-0.640200000

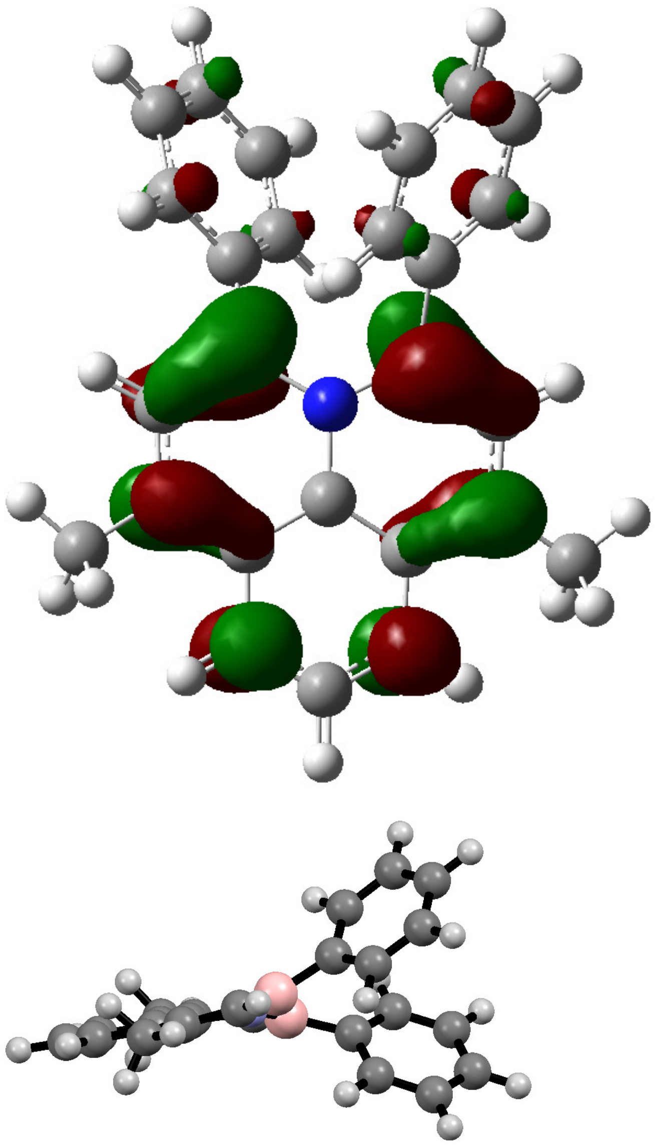

Figure S8: Top, LUMO of compound **3** (calculated at the B3LYP/6-311G(d,p) level), the different nature of this orbital relative to the SOMO of phenalenyl includes character on the exocyclic rings and greater bonding character in the main tricyclic core. This is in contrast to the essentially non-bonding nature of the LUMO in the 1-boraphenalenes reported herein and the phenalenyl cation. Bottom, the "side" view of the calculated structure, showing the deviation from planarity in the tricyclic core induced by the two exocyclic phenyl rings that are in the peri positions. This deviation can be quantified by the displacement from the mean plane of the C6 ring, which for the two boron attoms in the calculated structure above is 0.32 Å (in the previously reported solid state structure the displacement of these atoms is 0.27 Å from the mean plane of the C6 ring).[13] In contrast the boraphenalenes reported in this work are siginificantly more planar, e.g. in the solid state structure of compound **12** the maximum displacement on any atom in the tricyclic ring from the mean plane is 0.05 Å, this is due to the fact that the exocyclic substituents are not occupying the peri positions.

Table S3: Calculated LUMO energy and NICS(1) and NICS(1)$_{zz}$ values (B3LYP/6-311G(d,p)).

	Energy (eV)		NICS(1)/NICS(1)$_{zz}$		
	**HOMO**	**LUMO**	**Ring A**	**Ring B**	**Ring C**
**5c**	-6.33	-2.96	-9.9/-23.0	-10.4/-24.9	-1.1/2.6
**9**	-6.68	-3.07	-10.1/-23.5	-10.3/-24.5	-1.2/3.1
**12**	-6.01	-2.76	-9.9/-23.6	-10.3/-25.2	-0.9/1.9
**13**	-5.87	-2.55	-9.6/-23.4	-10.1/-25.3	-0.3/2.5
**14**	-5.94	-2.56	-10.2/-24.5	-10.4/-25.7	-0.6/1.9
**15**	-6.12	-2.84	-10.2/-24.1	-10.2/-24.8	-0.9/2.9
**1**$^{+}$	-11.52	-7.93	-7.8/-17.2	-7.8/-17.2	-7.8/-17.2
$C_{12}BH_9$	-6.38	-2.65	-10.1/-24.6	-10.4/-25.6	-1.6/-0.3

References

1 C. M. Cardona, W. Li, A. E. Kaifer, D. Stockdale and G. C. Bazan, *Adv. Mater.*, 2011, **23**, 2367–2371.

2 Gaussian 09, Revision C1, Frisch, M. J.; Trucks, G. W.; Schlegel, H. B.; Scuseria, G. E.; Robb, M. A.; Cheeseman, J. R.; Scalmani, G.; Barone, V.; Mennucci, B.; Petersson, G. A.; Nakatsuji, H.; Caricato, M.; Li, X.; Hratchian, H. P.; Izmaylov, A. F.; Bloino, J.; Zheng, G.; Sonnenberg, J. L.; Hada, M.; Ehara, M.; Toyota, K.; Fukuda, R.; Hasegawa, J.; Ishida, M.; Nakajima, T.; Honda, Y.; Kitao, O.; Nakai, H.; Vreven, T.; Montgomery, Jr., J. A.; Peralta, J. E.; Ogliaro, F.; Bearpark, M.; Heyd, J. J.; Brothers, E.; Kudin, K. N.; Staroverov, V. N.; Kobayashi, R.; Normand, J.; Raghavachari, K.; Rendell, A.; Burant, J. C.; Iyengar, S. S.; Tomasi, J.; Cossi, M.; Rega, N.; Millam, J. M.; Klene, M.; Knox, J. E.; Cross, J. B.; Bakken, V.; Adamo, C.; Jaramillo, J.; Gomperts, R.; Stratmann, R. E.; Yazyev, O.; Austin, A. J.; Cammi, R.; Pomelli, C.; Ochterski, J. W.; Martin, R. L.; Morokuma, K.; Zakrzewski, V. G.; Voth, G. A.; Salvador, P.; Dannenberg, J. J.; Dapprich, S.; Daniels, A. D.; Farkas, Ö.; Foresman, J. B.; Ortiz, J. V.; Cioslowski, J.; Fox, D. J. Gaussian, Inc., Wallingford CT, 2009.

3 http://comp.chem.umn.edu/info/DFT.htm, .

4 K. Pati, C. Michas, D. Allenger, I. Piskun, P. S. Coutros, G. dos Passos Gomes and I. V Alabugin, *J. Org. Chem.*, 2015, **80**, 11706–11717.

5 H. Yoshida, T. Morishita, H. Nakata and J. Ohshita, *Org. Lett.*, 2009, **11**, 373–376.

6 L. Meng, T. Fujikawa, M. Kuwayama, Y. Segawa and K. Itami, *J. Am. Chem. Soc.*, 2016, **138**, 10351–10355.

7 R. Gelin and D. Pigasse, *Bull. Soc. Chim. Fr.*, 1971, 1840–1847.

8 *CrysAlisPro, Agilent Technologies, Version 1.171.35.19 (release 27-10-2011 CrysAlis171 .NET) (compiled Oct 27 2011,15:02:11)*, .

9 O. V Dolomanov, L. J. Bourhis, R. J. Gildea, J. A. K. Howard and H. Puschmann, *J. Appl. Cryst.*, 2009, **42**, 339–341.

10 L. Palatinus and G. Chapuis, *J. Appl. Cryst.*, 2007, **40**, 786.

11 G. M. Sheldrick, *Acta Cryst. Sect. A*, 2015, **A71**, 3–8.

12 G. M. Sheldrick, *Acta Cryst. Sect. C*, 2015, **C71**, 3–8.

13 H. Wei, Y. Liu, T. Y. Gopalakrishna, H. Phan, X. Huang, L. Bao, J. Guo, J. Zhou, S. Luo, J. Wu, Z. Zeng, *J. Am. Chem. Soc*. 2017, **139**, 15760-15767.
